# *Salmonella enterica* serovar Braenderup shows clade-specific source associations and a high proportion of molecular epidemiological clustering

**DOI:** 10.1128/aem.02594-24

**Published:** 2025-03-21

**Authors:** Harleen K. Chaggar, Lauren K. Hudson, Kelly Orejuela, Linda Thomas, Maya Spann, Katie N. Garman, John R. Dunn, Thomas G. Denes

**Affiliations:** 1 Department of Food Science, University of Tennessee 4292 , Knoxville, Tennessee, USA; 2 Tennessee Department of Health 5719 , Nashville, Tennessee, USA; 3 Division of Laboratory Services, Tennessee Department of Health 5719 , Nashville, Tennessee, USA; The Pennsylvania State University, University Park, Pennsylvania, USA

**Keywords:** *Salmonella*, Braenderup, genomics, antimicrobial resistance, molecular epidemiology

## Abstract

*Salmonella enterica* serovar Braenderup (*S. enterica* ser. Braenderup) is an important clinical serovar in the United States. This serovar was reported by the CDC in 2017 as the fifth most common *Salmonella enterica* serovar associated with outbreaks in the United States, which have been linked to both fresh produce and food animal products. The goals of this study were to compare the relatedness of human clinical isolates from southeastern USA (Tennessee (*n* = 106), Kentucky (*n* = 48), Virginia (*n* = 252), South Carolina (*n* = 109), Georgia (*n* = 159), Alabama (*n* = 8), Arkansas (*n* = 26), and Louisiana (*n* = 91)) and global clinical (*n* = 5,153) and nonclinical (*n* = 1,053) isolates obtained from the NCBI. Additionally, we also examined the population structure of *S. enterica* ser. Braenderup strains (*n* = 3,131) on EnteroBase and found that all the strains of this serovar are associated with a single cgMLST eBurst group (ceBG 185), confirming that this serovar is monophyletic. We divided the *S. enterica* ser. Braenderup population into two clades (Clade I and Clade II) and one clade group (Clade Group III). The composition of distinct environmental isolates in the clades differed: Clade I was significantly associated with produce (90.7%; *P* < 0.0001) and water, soil, and sediment (76.9%; *P* < 0.0001), and Clade II was significantly associated with poultry environments (62.8%; *P* < 0.0001). The clade-specific gene associations (e.g., Clade I-associated competence proteins and cytochrome_c_asm protein and Clade II-associated heme-exporter protein and dimethyl sulfoxide [DMSO] reductase-encoding genes) provide potential insights into possible mechanisms driving environmental adaptation and host–pathogen interaction. Phylogenetic analyses identified 218 molecular epidemiological clusters in the current study, which represented a greater proportion of potentially outbreak-related isolates than previously estimated.

**IMPORTANCE:**

This study provides insights into the genomic diversity of *S. enterica* ser. Braenderup by revealing distinct clade-specific source attribution patterns and showing that a greater proportion of isolates were associated with epidemiological clusters based on the genomic relatedness than previously estimated. Specifically, we analyzed the diversity of human clinical isolates from southeastern USA and compared them with the global clinical and nonclinical isolates. Our analysis showed different clades of *S. enterica* ser. Braenderup linked to different environments, providing insights on the potential source of human sporadic infection and outbreaks. These findings can enhance public health surveillance and response strategies targeting *S. enterica* serovar Braenderup by expanding our understanding of potential transmission pathways and the genomic diversity of clinical and environmental isolates.

## INTRODUCTION

*Salmonella,* a Gram-negative bacterium in the family Enterobacteriaceae (1, 2), consists of two species: *Salmonella enterica* and *Salmonella bongori* (3, 4). *Salmonella enterica* is further divided into six subspecies (3) and comprises over 2,700 serotypes based on the White–Kauffmann–Le Minor scheme (5, 6). Serovars of *S. enterica* have been characterized as monophyletic or polyphyletic (7, 8)) based on multi-locus enzyme electrophoresis (MLEE) (9, 10) and multi-locus sequence typing (MLST) schemes (11, 12). Previous studies on the phylogeny of *S. enterica* that have included *S. enterica* serovar Braenderup (*S. enterica* ser. Braenderup) show results that are suggestive of it being a monophyletic serovar (8, 10).

According to the 2016 National Enteric Disease Surveillance Salmonella Annual Report (13), *S. enterica* ser. Braenderup was the ninth ranked serovar among culture-confirmed human *Salmonella* infections reported to The Laboratory-based Enteric Disease Surveillance (LEDS) system, with 1,001 cases reported, an 82% increase from the 550 cases reported in 2006. In 2017, the Centers of Disease Control and Prevention (CDC) ranked this serovar fifth among all confirmed *Salmonella enterica* outbreaks, accounting for 5% of the total 112 outbreaks (14). Additionally, *S. enterica* ser. Braenderup is notably more prevalent in the southeastern region of the USA, with particularly higher incidence rates in states such as Georgia, North Carolina, and Virginia, according to the 2016 National Enteric Disease Surveillance Report (13).

*Salmonella enterica* ser. Braenderup has been associated with a diversity of sources. These sources included fresh produce (iceberg lettuce (15), coriander, lettuce, tomato, and cucumber [16–18]) and numerous animal exposures (turtles [19, 20], hatcheries [21], cattle and deer [22], snakes [23], equines [24], feces of rapaces and cockroaches [25, 26], wild and domesticated animal feces [27], turkey vultures [25], and pigeons [28]). Food animals and food animal products (poultry [29], beef, mutton, pork [19, 30, 31]); animal feeds; and feed supplements (32) have also been implicated as well as environmental facilities/farms (open environmental spaces [33], farms [34, 35], animal houses [36], and processing facilities [37]). Other sources include water and soil (rivers, run-off water, surface waters, soil sediments on farms, and associated supply chains [33, 38, 39]).

Outbreaks caused by *S. enterica* ser. Braenderup have involved diverse geographic areas, from multi-continent outbreaks to local-level outbreaks. Notably, a large multicountry outbreak of *S. enterica* ser. Braenderup was linked to contaminated melons from Honduras, which affected hundreds across multiple countries (40, 41). In 2004, another multicountry outbreak linked to contaminated Roma tomatoes also led to numerous illnesses in North America (42, 43). This serovar has been involved in international outbreaks since the early 1990 s, including airline foods (44), contaminated jelly in meat pies (26), and boxed lunches (45). In the United States, this serovar has caused multistate foodborne outbreaks linked to a range of food commodities such as mangoes (46), nut butter (47), shell eggs (48), mung bean sprouts (49), fresh smoothies (50) (51), and chicken salad (52). Additionally, *S. enterica* ser. Braenderup has also caused local foodborne outbreaks linked to dairy (53), juice products (54), and potato salad (55).

Salmonellosis is typically a self-limiting disease, but people with weakened immune systems and severe illnesses may require hospitalization, fluids, or antibiotics (56). The CDC lists the following antibiotics for nontyphoidal *Salmonella*: ciprofloxacin, azithromycin, ceftriaxone, ampicillin, and trimethoprim-sulfamethoxazole (57). In 2019, the CDC reported a concerning trend of nontyphoidal *Salmonella* becoming less susceptible to key antibiotics and a rise in drug-resistant nontyphoidal *Salmonella* infections, causing an estimated 212,500 illnesses and 70 deaths annually in the United States (57).

In this study, we leveraged publicly available global *S. enterica* ser. Braenderup genomic surveillance data to (i) evaluate the population structure of *S. enterica* ser. Braenderup in the context of other *Salmonella* serovars, (ii) perform phylogenetic analyses of clinical and nonclinical *S. enterica* ser. Braenderup isolates from southeastern USA and other global locations to better understand source attribution and geographical trends, (iii) identify molecular epidemiological clusters using hqSNP thresholds relevant to public health surveillance, and (iv) determine predicted antibiotic resistance patterns.

## MATERIALS AND METHODS

### Data collection, read processing, trimming, and genome assembly

We obtained identifiers (PNUSA or BioSample IDs) and the associated metadata of sequenced clinical *S. enterica* ser. Braenderup isolates provided by participating southeastern United States public health departments (*n* = 799). These southeastern USA states are as follows: Tennessee (*n* = 106), Kentucky (*n* = 48), Virginia (*n* = 252), South Carolina (*n* = 109), Georgia (*n* = 159), Alabama (*n* = 8), Arkansas (*n* = 26), and Louisiana (*n* = 91). These isolates were collected from 2015 to 2022 (704 did not have collection dates on the NCBI). The raw reads for all the clinical southeastern USA isolates were downloaded from the NCBI Sequence Read Archive (SRA) database, and adapter sequences were trimmed using Trimmomatic (v0.39; parameters: ILLUMINACLIP: NexteraPE-PE.fa:2:30:10 LEADING:3 TRAILING:3 SLIDINGWINDOW:4:15 MINLEN:36) (58). Next, FASTQC (v0.11.9) (59) was used to check trimmed reads to determine read quality and combined into a single report using MultiQC (v1.11). SPAdes (60) was used to assemble quality-checked paired-end reads, and assembly statistics were determined using QUAST (v5.0.2) (61) (Table S1).

To better understand the global *S. enterica* ser. Braenderup population structure, we downloaded information of clinical and nonclinical genomic assemblies from the NCBI Pathogen Detection Isolates Browser, queried using the following filters: isolation type “clinical” (for clinical isolates) or “environmental/other and empty” (for nonclinical isolates) and serovar “Braenderup/*Salmonella enterica* subsp. *enterica* serovar Braenderup” and selected all the available isolation source types. We downloaded an extensive data set (*n* = 6,206; excluding clinical southeastern USA) comprising clinical (*n* = 5,153) and nonclinical (*n* = 1,053) isolates (as of 15 July 2022) from global locations including regions other than the United States (excluding clinical southeastern USA), North America, South America, Europe, Africa, and Asia. These isolates were collected from 1987 to 2022 (3,494 did not have collection dates on the NCBI). Isolates were excluded from the study if they did not meet all of the following inclusion criteria: total genome length of 4.5 to 5.1 Mb, ≤150 contigs, ≥10 × coverage (62–65), and 51.9%–52.5% G + C content, or (for the NCBI-obtained isolates) if their genomic assemblies were not available on the NCBI. Assembly statistics were determined using QUAST (v5.0.2) (61) (Table S2). SeqSero2 (62) and The *Salmonella In Silico* Typing Resource (SISTR) (66) were used to predict serovars. Both clinical and nonclinical isolates obtained from the NCBI that were sourced from the southeastern United States, including Alabama (AL), Arkansas (AR), Georgia (GA), Kentucky (KY), Louisiana (LA), South Carolina (SC), Tennessee (TN), and Virginia (VA), were classified as southeastern USA isolates.

### Phylogenetic analyses of *S. enterica* ser. Braenderup

**Fig 1:**
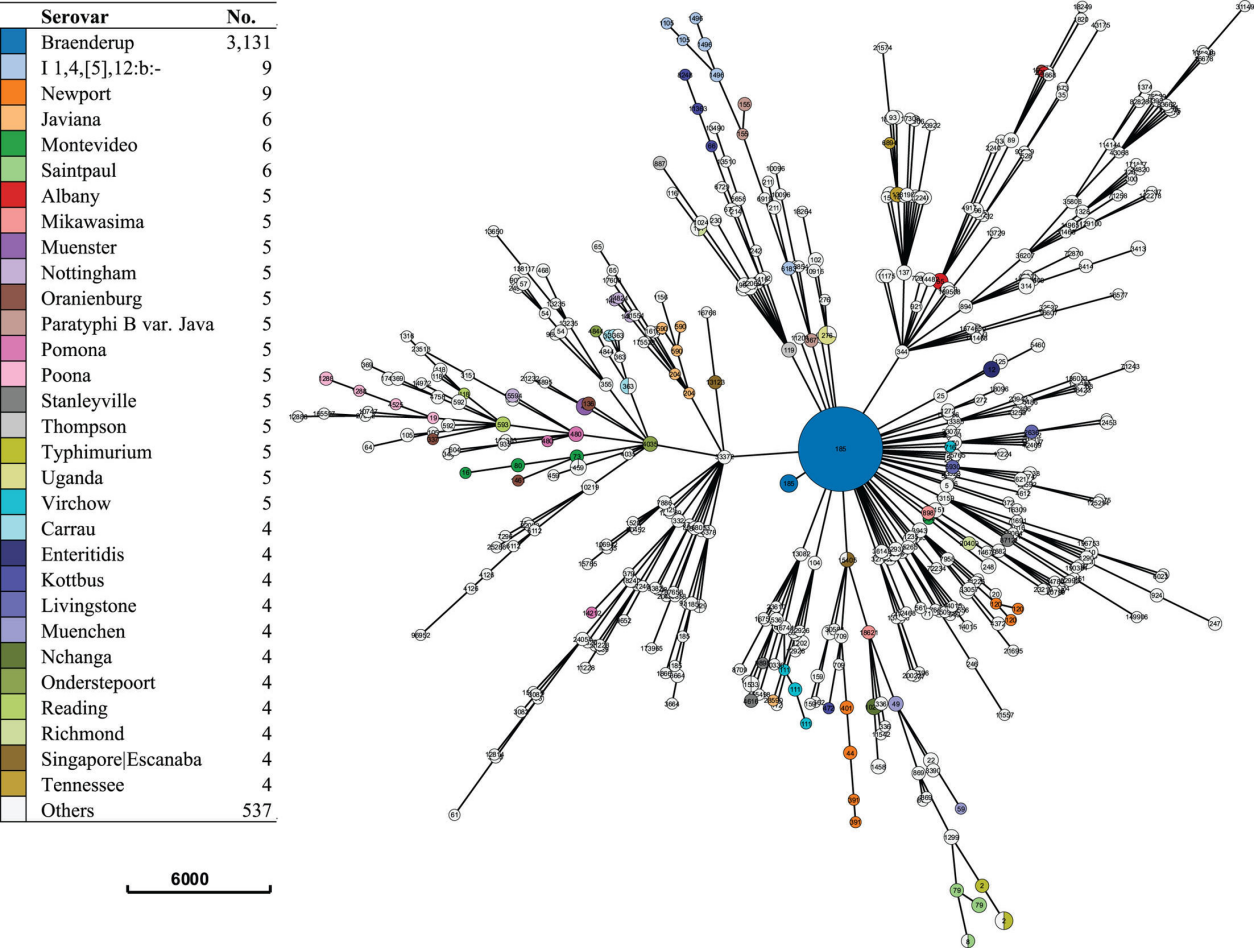
Population structure of *Salmonella enterica* serovar Braenderup and other *Salmonella enterica* serovars. The figure depicts cgMLST +HierCC minimal spanning tree (MSTree V2 algorithm). This tree consists of *Salmonella enterica* serovars using the SISTR1 Serovar (Serotype(SISTR1 +SeqSero2)) algorithm that was created with GrapeTree on EnteroBase using 3,813 isolates.

A data set was created by identifying all available *S. enterica* ser. Braenderup isolates (*n* = 3,131) in the *Salmonella* database on EnteroBase, classified based on serovar identification by SISTR1 and/or SeqSero2. A reference data set containing reference strains (*n* = 682) that represented 407 distinct *Salmonella enterica* serovars (described in 67, 68) was also compiled for further phylogeny comparison. A cgMLST +HierCC minimal spanning tree (Fig. 1) (MSTree V2 algorithm) was created with GrapeTree on EnteroBase using all isolates (*n* = 3,813; EnteroBase workspace: https://enterobase.warwick.ac.uk/species/senterica/search_strains?query=workspace:98928) (Table S3). The branch lengths between the cgMLST eBurstGroups (ceBGs) of the other polyphyletic serovars were used for comparison to the branch length between the two *S*. *enterica* ser. Braenderup ceBGs. ceBG (cgMLST eBurstGroup) designations associated with each *Salmonella* serovar were obtained from EnteroBase documentation (69).

### SNP detection and global phylogenetic analyses

kSNP3.1 (70), a reference-free single-nucleotide polymorphism (SNP)-based method, was used to analyze all the clinical and nonclinical genomic assemblies. In the final phylogenetic analysis, we included a total of the clinical (*n* = 801) and nonclinical (*n* = 170) southeastern USA isolates and global clinical (*n* = 6,513) and nonclinical (*n* = 948) genomic assemblies from different continents (Table 1). After removing the isolates that did not meet the inclusion criteria, the first kSNP analysis contained a total of 6,940 isolates. This phylogenetic tree contained isolates (*n* = 387) that were more divergent on the tree (>1,000 SNPs) than all the remaining isolates. These putative non-*S*. *enterica* ser. Braenderup genomes were further evaluated to determine if they should be excluded from further analyses. The “Similar Genome Finder” tool on PATRIC (available through The Bacterial and Viral Bioinformatics Resource Center) (71), which uses the Mash/MinHash algorithm, was used to identify similar publicly available genomes. PYANI (v0.2.12) (72) was used to calculate the average nucleotide identity (ANI) between the more divergent genomes, *S. enterica* ser. Braenderup reference strains (RefSeq complete genomes were obtained from NCBI), and the similar genomes identified in PATRIC. BactaxR (73) was then used to construct an ANI dendrogram. Based on the ANI results, the divergent isolates likely belonged to various other *S. enterica* serovars, including Claibornei, Sandiego, Typhimurium-var5, Typhimurium, Typhi, and Cerro. Since these isolates were determined to be putative non-*S*. *enterica* ser. Braenderup (based on ANI), we removed these genomes from the SNP analysis and re-ran kSNP3. The output phylogenetic tree contained additional, more divergent *S. enterica* ser. Braenderup isolates (*n* = 12; ≥800 SNPs). After evaluating and removing these 12 isolates, kSNP3 was re-run with the remaining genomes (*n* = 6,541). Next, five additional isolates with ≥90 branch lengths were evaluated and removed from the analysis, and kSNP3 phylogenetic analysis was re-run with 6,436 isolates.

**TABLE 1:** Total isolates from clinical and nonclinical sources and isolation locations

Source location	No. of isolates		
	Clinical	Nonclinical	Unknown
**Global**	**980**	**132**	**1**
Africa	1	18	
Asia	11	11	
Europe	557	17	
Other North America	161	41	
South America	2	28	
Unknown	248	17	1
**USA, other states**	**3,788**	**687**	
**USA, Southeastern states**	**790**	**158**	
Alabama	8	7	
Arkansas	24	9	
Georgia	157	59	
Kentucky	43	7	
Louisiana	90	4	
South Carolina	116	8	
Tennessee	104	5	
Virginia	248	59	
**Total**	**5,558**	**977**	**1**

**Fig 2:**
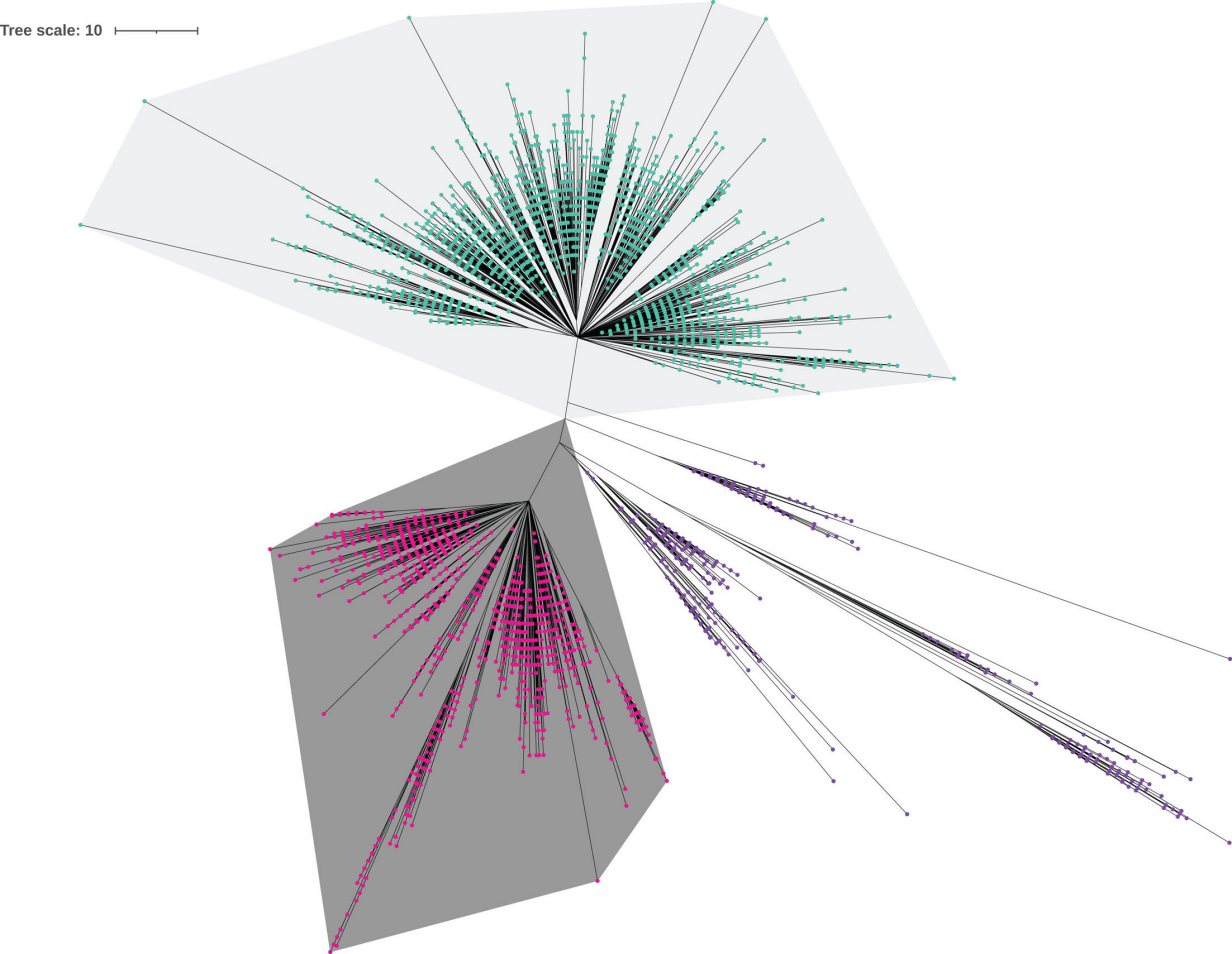
Global population structure of *Salmonella enterica* serovar Braenderup. Global population structure based on core SNPs, with clades shaded as follows: Clade I: n=3,593 (light gray), Clade II: n=2,261 (dark gray), Clade Group III: n=682 (no shading). Tree is based on core SNPs, as determined by KSNP3. The evolutionary history was inferred using the neighbor-joining method. The optimal tree is shown. The tree is drawn to scale, with branch lengths in the same units as those of the evolutionary distances used to infer the phylogenetic tree. The evolutionary distances were computed using the number of differences method and are expressed in the units of the number of base differences per sequence. This analysis involved 6,536 nucleotide sequences. All positions containing gaps and missing data were eliminated (complete deletion option). There were a total of 29,314 positions in the final data set. Evolutionary analyses were conducted in MEGA X. The population was divided into clades using FastBaps.

**Fig 3:**
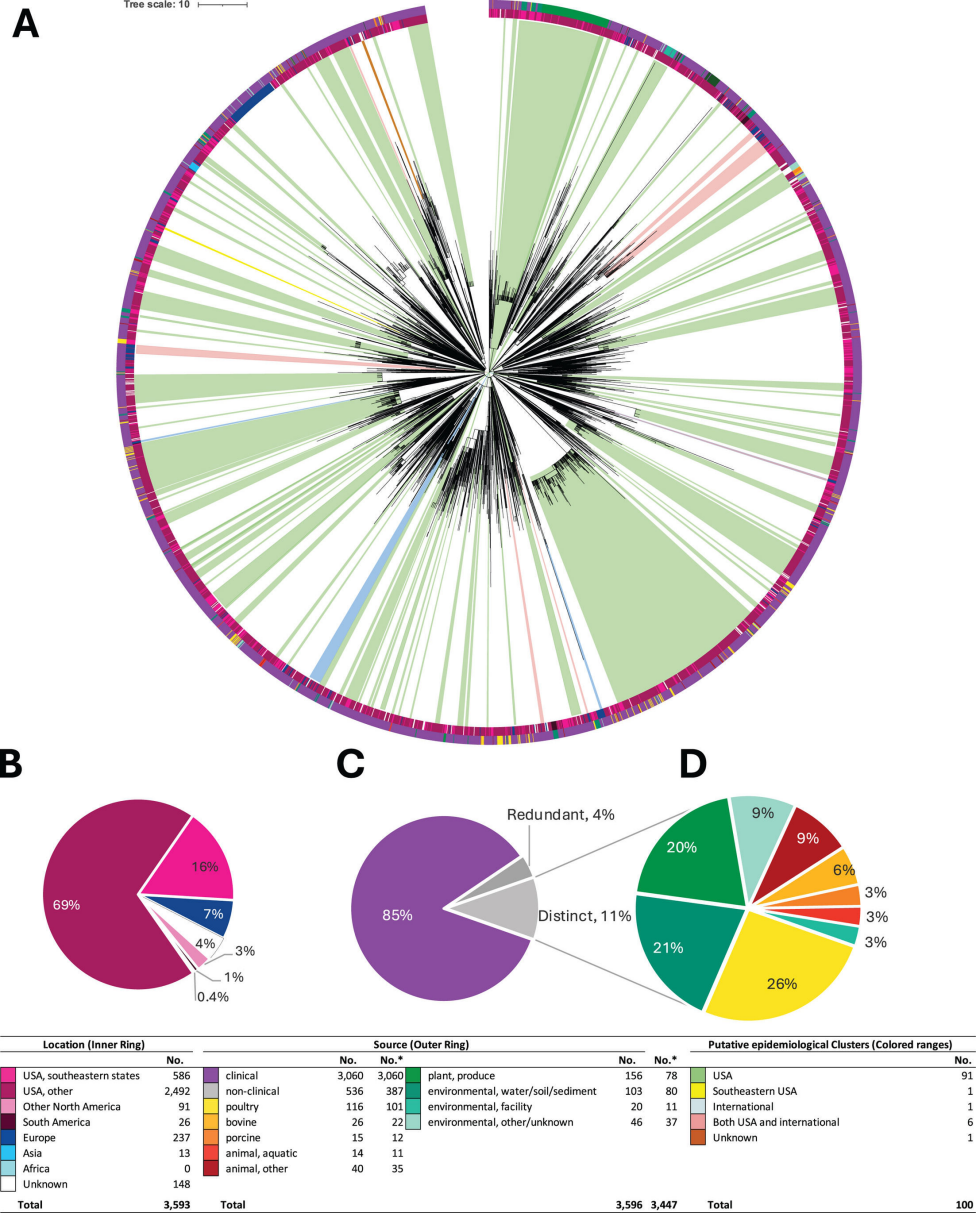
Phylogeny, source, location, and potential epidemiological clusters for *Salmonella enterica* serovar Braenderup isolates in clade I. (**A**) Neighbor-joining phylogenetic trees. Each tree is marked with labeled geography (inner ring) and source attribution (outer ring) of all the isolates on the trees. The colored ranges depict molecular epidemiological clusters in each clade. See the legend at the bottom for colors. The trees were created in Mega X, annotated, and edited with iTOL. (**B**) Percentage of Clade I isolates from each location. (**C**) Percentage of Clade I isolates from clinical and nonclinical sources. (**D**) Percentage of Clade I nonclinical isolates from each source. No.* represents the number of isolates after the redundant environmental isolates were removed for source attribution analysis.

**Fig 4:**
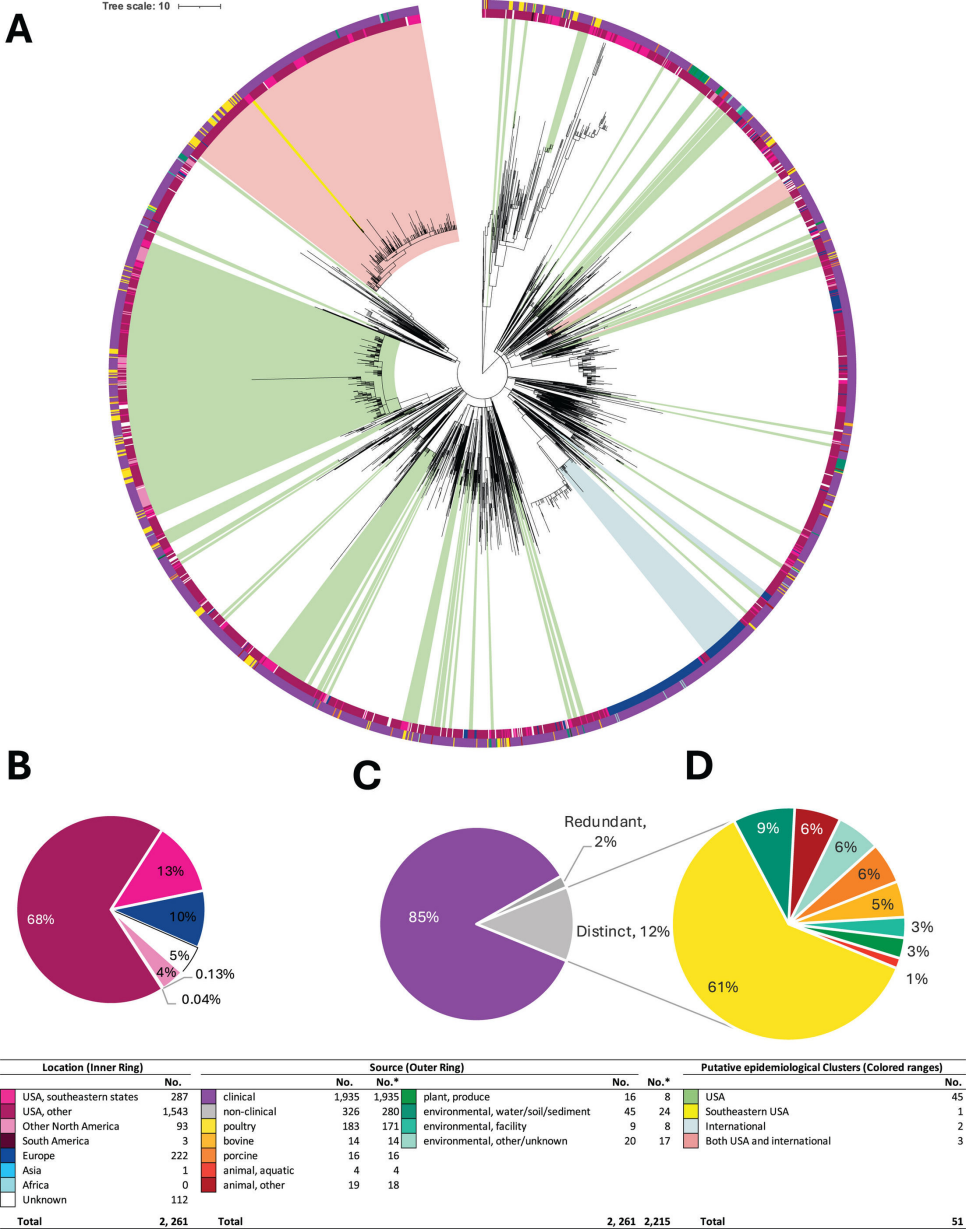
Phylogeny, source, location, and potential epidemiological clusters for *Salmonella enterica* serovar Braenderup isolates in Clade II. (**A**) Neighbor-joining phylogenetic trees. The tree is marked with labeled geography (inner ring) and source attribution (outer ring) of all the isolates on the trees. The colored ranges depict molecular epidemiological clusters in each clade. See the legend at the bottom for colors. The trees were created in Mega X, annotated, and edited with iTOL. (**B**) Percentage of Clade II isolates from each location. (**C**) Percentage of Clade II isolates from clinical and nonclinical sources. (**D**) Percentage of Clade II nonclinical isolates from each source. No.* represents the number of isolates after the redundant environmental isolates were removed for source attribution analysis.

**Fig 5:**
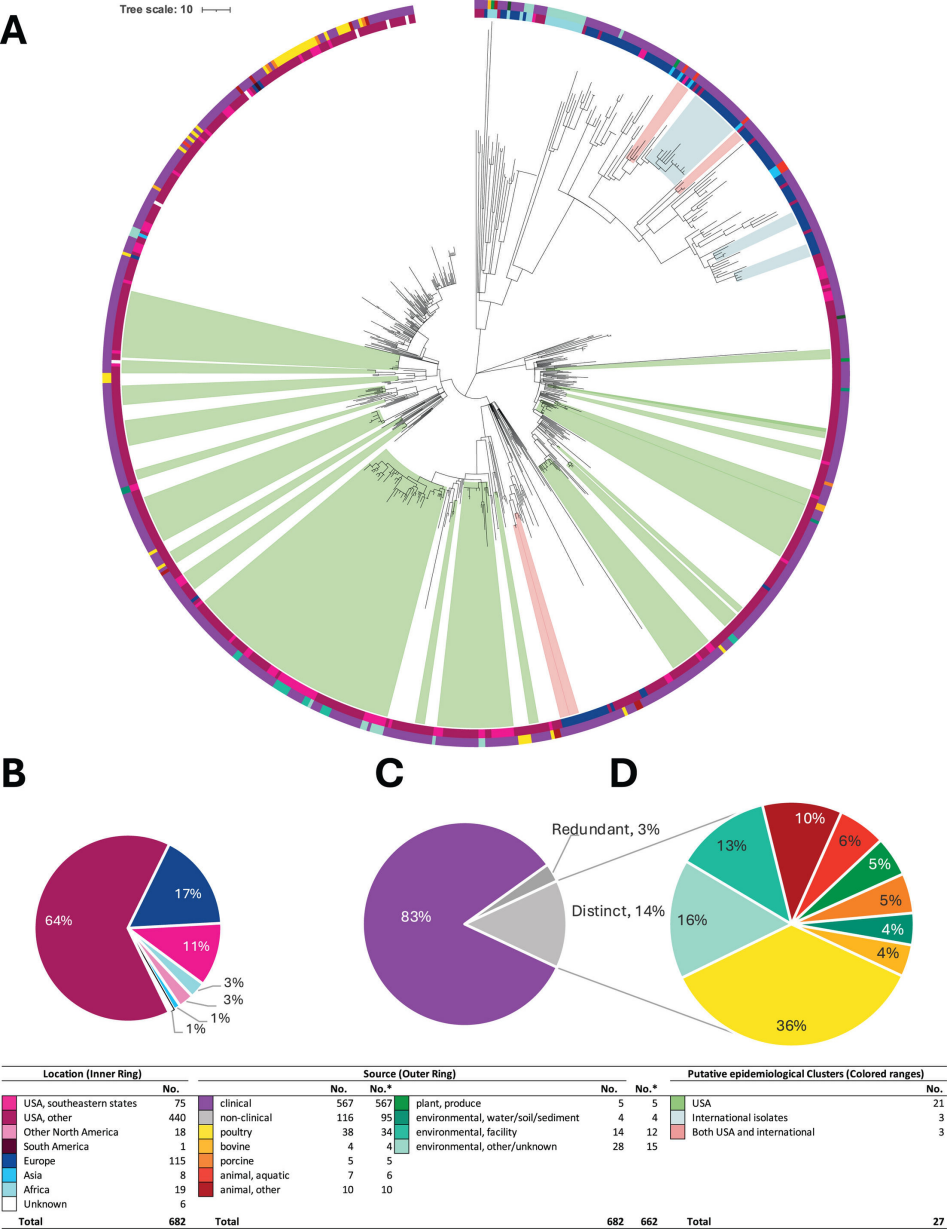
Phylogeny, source, location, and potential epidemiological clusters for *Salmonella enterica* serovar Braenderup isolates in Clade group III. (**A**) Neighbor-joining phylogenetic trees. The tree is marked with labeled geography (inner ring) and source attribution (outer ring) of all the isolates on the trees. The colored ranges depict molecular epidemiological clusters in each clade. See the legend at the bottom for colors. The trees were created in Mega X, annotated, and edited with iTOL. (**B**) Percentage of Clade group III isolates from each location. (**C**) Percentage of Clade II isolates from clinical and nonclinical sources. (**D**) Percentage of Clade group III nonclinical isolates from each source. No.* represents the number of isolates after the redundant environmental isolates were removed for source attribution analysis.

Next, FastBAPS (v1.0.8) (74) was used to divide the population (*n* = 6,436) into clades or subclades. The population was divided into two clades (Clade I with 3,593 isolates and Clade II with 2,261 isolates) and one clade group (682 isolates) (Fig. 2). The clade group consisted of multiple distinct clades; therefore, it was named Clade Group III. KSNP3 was run on each clade/clade group individually, using the optimum Kmer size for each determined using the Kchooser utility (Clade I: k = 19, Clade II: k = 31, and Clade group III: k = 31). The final kSNP phylogenetic trees for individual clades were calculated using MEGA X (v10.2.6) (75), then edited, annotated, and visualized using iTOL (76) (Fig. 3 to 5).

### Environmental source analyses

To reduce sampling bias from the repetitive inclusion of closely related isolates, we removed redundant environmental isolates with zero core genome SNP differences that were isolated from the same environmental source, location, and year. For each group of such isolates, we retained a representative isolate with the fewest number of contigs. A total of 212 redundant isolates were removed, leading to a final data set of 762 environmental isolates for source analysis (Table S4).

A one-proportion test for each isolation source category in Clades I and II was conducted in SAS 9.4 (for Windows 64 x, SAS Institute Inc., Cary, NC). Clade Group III was excluded as it is not a single phylogenetic clade and would not be expected to have a group-specific phenotype. Significance was identified at *P* < 0.05 (Table 2).

**TABLE 2:** One proportion statistical test for each isolation source category in Clades I and II

Isolation source	Clade I		Clade II		*P*-value^*c*^
	No. of isolates (% of clade total)^*a*^	Proportion of source within clade (%)^*b*^	No. of isolates (% of clade total)	Proportion of source within clade (%)	
Animal, aquatic	11 (3%)	73.3	4 (1%)	26.6	0.0354
Animal, other	35 (9%)	66	18 (6%)	33.9	0.0098
Bovine	22 (6%)	61.1	14 (5%)	38.8	0.0912
Environmental, facility	11 (3%)	57.8	8 (3%)	42.1	0.2456
Environmental, other/unknown	37 (9%)	68.5	17 (6%)	31.4	0.0032
Environmental, water/soil/sediment	80 (21%)	76.9	24 (9%)	23	<0.0001
Plant, produce	78 (20%)	90.7	8 (3%)	9.3	<0.0001
Porcine	12 (3%)	42.8	16 (6%)	57.1	0.2248
Poultry	101 (26%)	37.1	171 (61%)	62.8	<0.0001
**Total**	**387**		**280**		

^*a*^ Represents the number of isolates and the percentage from each source assigned to the respective clade.

^*b*^ Represents the percentage from a one-proportion statistical test comparing the isolate proportions between Clades I and II.

^*c*^ Represents the significance of the one-proportion test, where *P*-values less than 0.05 indicate significant differences between the clades for a specific source.

### Genome annotation and pan-genome-wide association study (pan-GWAS)

All the genomic assemblies were annotated with Prokka v1.14.6 (77). Treemmer v0.3 (78) was used to select 100 genomes representing the total diversity from each clade/clade group phylogenetic tree (parameters: pruned down to 100 tips). Gene content among the 300 genomes was compared using Orthofinder v2.2.0 (79) with the annotated FASTA files from Prokka (Table S7). Scoary2 v0.015 (80) was used to identify genes associated with Clades I and II based on the odds ratio (>1 or <1) (Table S8). Functional annotation of the orthologs was performed using eggNOG-mapper v2.1.12 (81, 82). Orthologs that were not assigned eggNOG-mapper functions were further searched using Protein Blast (83) and InterProScan (84) (Table S8).

### High-quality single-nucleotide polymorphism analyses and molecular epidemiological cluster identification

First, kSNP3 outputs and ClustFinder v1.0 (85) were used to identify molecular epidemiological clusters that contained ≥3 clinical isolates within 15 SNPs. In the present study, we defined “molecular epidemiological clusters” solely based on the genetic similarity of the isolates, i.e., SNP distance thresholds, without considering their isolation dates or any other additional epidemiological data. Next, for each potential cluster, a high-quality single-nucleotide polymorphism (hqSNP) analysis was performed using the CFSAN SNP pipeline (v2.2.1) (86). A custom Bash script was used to choose an internal reference genome for each cluster based on the following parameter(s): (a) lowest average SNP distance and (b) if two or more genomes have the same lowest average SNP distance, it chooses the genome that has the lowest number of contigs among them. In this study, the hqSNP distance used to define a molecular epidemiological cluster was ≤10 hqSNPs, and a cluster was confirmed only if all the clinical isolates were within that hqSNP distance from another clinical isolate. Conversely, any cluster in which all the isolates exceeded the hqSNP threshold (≤10 SNPs) was not considered a cluster and was not evaluated any further. Additionally, if some isolates in a cluster exceeded the hqSNP distance threshold of ≤10 hqSNPs, then that cluster was further re-clustered with ClustFinder v1.0 (85) using the same parameters (≤10 SNPs and ≥3 clinical isolates) and potentially divided into sub-clusters. This process was reiterated for these potential clusters until they were confirmed as molecular epidemiological clusters or eliminated.

### Antimicrobial resistance patterns of southeastern United States isolates

Antibiotic resistance (ABR) determinants were identified in the assemblies (*n* = 6,536) using ResFinder (87) and PointFinder (88) (v4.2.0; with an identity threshold of 0.8 and minimum coverage of 0.6). Based on the ResFinder outputs, the original prediction for amikacin resistance was 100% due to all isolates containing the *aac(6′)-Iaa* gene. However, this gene is reported to be cryptic, with studies suggesting that it may have reached its evolutionary limits and therefore no longer confers phenotypic resistance to aminoglycosides (24, 89–91). Therefore, any isolate that contained this cryptic gene and no other aminoglycoside resistance determinants was removed from the final reporting of the predicted aminoglycoside resistance. Statistical analyses were conducted in SAS (for Windows 64 x, SAS Institute Inc., Cary, NC, USA). Chi-square tests were completed to determine whether there were significant differences among the antibiotic classes across different sources, locations, and clades. The non-antibiotic classes (aldehydes, heat, peroxide, and quaternary ammonium compounds) were not included in the statistical analysis. We used the GLIMMIX procedure to analyze the effect of clades, isolation sources, and isolation location on the antibiotic resistance patterns. Differences between these three categories were evaluated using the Tukey–Kramer multiple comparison procedure. Additionally, the NARMS human clinical ABR data were downloaded from NARMS Now (92) for comparison.

## RESULTS

### Phylogenetic analyses confirm that *S. enterica* ser. Braenderup is monophyletic

The phylogenetic analysis included all the available *S. enterica* ser. Braenderup strains (*n* = 3,131) and reference strains representing 407 other *S. enterica* serovars (*n* = 682) on EnteroBase (Table S3). A cgMLST +HierCC minimal spanning tree (MSTree V2 algorithm) was created using this diverse data set of strains (*n* = 3,813). EnteroBase determines population groups, such as cgMLST eBurst groups (ceBGs) that are associated with *S. enterica* serovars (69). Based on our analyses, we found that all *S. enterica* ser. Braenderup isolates were classified under a single ceBG: 185 (Fig. 1). Next, based on the cgMLST minimal spanning tree, we found that all *S. enterica* ser. Braenderup isolates clustered together and that the longest branch length between two *S*. *enterica* ser. Braenderup isolates was 460 allelic differences. Additionally, no non-Braenderup isolates in the data set belonged to the 185 ceBG nor clustered with the Braenderup isolates in the cgMLST minimal spanning tree.

### Phylogeny consists of two clades and one clade group

In the present study, we analyzed a population of 6,536 *S. enterica* ser. Braenderup isolates, comprising both clinical (*n* = 5,558) and nonclinical (*n* = 978) isolates. We divided this population from our phylogenetic analysis using FastBAPS. This resulted in two main clades (Clade I with 3,593 isolates and Clade II with 2,261 isolates) and an additional clade group that contained multiple smaller distinct clades (referred to as Clade Group III, with 682 isolates) (Fig. 2 to 5; detailed phylogenetic trees with branch lengths, bootstrap values, and isolate IDs are available in Fig. S1 to S3). All clades primarily contained isolates from the United States (Clade I 69.3% non-southeastern USA and 16.3% southeastern USA; Clade II 68.2% non-southeastern USA and 12.7% southeastern USA; Clade Group III 64.5% non-southeastern USA and 10.9% southeastern USA), followed by Europe (Clade I 6.6%, Clade II 9.8%, and Clade Group III 16.9%). However, Clade Group III exhibited the broadest geographical diversity among the clades as it also contained 2.8% isolates from Africa and 1.2% from Asia (compared to 0.4% Asian isolates in Clade I, 0.04% Asian isolates in Clade II, and no African isolates in either clade). Other geographical locations, such as Other North America, South America, Asia, and Africa, had a smaller proportional representation (each less than 3%) in both the clades and the clade group.

### Environmental isolate sources differed between major clades

Notably, we found that the composition of environmental isolates in each major clade differed from one another (Table 2). After excluding redundant genomes, the environmental isolates in Clade I were primarily sourced from poultry (26%), water, soil, and sediment (21%), and produce (20%) (Fig. 3D). A higher proportion of isolates sourced from water, soil, and sediment (76.9%; *P* < 0.0001) and produce (90.7%; *P* < 0.0001) were associated with Clade I over Clade II. Additionally, environmental isolates in Clade II were primarily sourced from poultry (61%) and water, soil, and sediment (9%) (Fig. 4D). A higher proportion of isolates sourced from poultry (62.8%; *P* < 0.0001) were associated with Clade II over Clade I. These findings emphasize a strong association of Clade I with produce environments and environmental isolates from water, soil, and sediment, whereas Clade II was associated with environmental isolates from poultry.

### Clade-enriched genes

Ortholog analysis of the representative genomes for Clade I (*n* = 100) and Clade II (*n* = 100) identified 6,187 orthogroups, representing 99.9% of the 886,169 total genes (Table S7). Out of 6,187 orthogroups, 3,805 were present in all the genomes (core orthogroups). Among these, 3,326 orthogroups were single-copy orthogroups, indicating each genome contributed exactly one gene copy to these orthogroups. A total of 32 orthogroups had significant associations to specific clades, including 22 significantly associated with Clade I and 10 with Clade II (Table S8). A total of those 12 orthogroups have functions associated with bacteriophages, including genes encoding phage structural proteins and enzymes (e.g., phage integrase, tyrosine recombinase, phage holin proteins, phage lysis proteins, phage replication genes, tail fiber assembly proteins, and regulatory phage proteins). These phage orthogroups were enriched in both Clade I (*n* = 8) and Clade II (*n* = 4). In addition to phage-related genes, Clade I was enriched with orthogroups encoding Competence Protein EC (ComEC), Cytochrome_c_Asm, and membrane-bound proteins. Clade II was enriched with orthogroups encoding heme exporter proteins, dimethyl sulfoxide (DMSO) reductase genes, biotin-dependent carboxyltransferase genes, and membrane-bound proteins.

### Composition and distribution of molecular epidemiological clusters

From our initial phylogenetic analyses employing a threshold of 15 SNPs, followed by high-quality SNP (hqSNP) analyses using a more stringent threshold of 10 hqSNPs, we identified a total of 218 molecular epidemiological clusters (Fig. 3 to 5; detailed phylogenetic trees with cluster IDs are available in Fig. S4 to S6). The molecular epidemiological clusters contained a total of 2,417 (85%) clinical isolates and 437 environmental isolates (15%). We identified 139 molecular epidemiological clusters in Clade I, 52 in Clade II, and 27 in Clade Group III. Furthermore, of the 218 identified molecular epidemiological clusters, 179 contained only clinical isolates (ranging from 3 to 48 clinical isolates).

Additionally, we found that of the total southeastern USA clinical isolates (*n* = 790) in the present study, 37.8% were found to be in molecular clusters (*n* = 299), whereas of the other (non-southeastern) USA clinical isolates (*n* = 3,788), 45.2% isolates were found to be in molecular clusters (*n* = 1,713). We identified that only two molecular clusters had clinical isolates exclusively from southeastern USA (both only contained TN isolates), and 34 molecular clusters had isolates exclusively from the USA and southeastern USA. Additionally, 39 molecular clusters also contained environmental isolates in addition to clinical isolates. Of those, 14 molecular clusters were found to contain environmental isolates sourced from only poultry environments. One molecular cluster (II_CL012-a) contained 54 environmental isolates, of which 50 were from poultry environments. Four molecular clusters (III_CL004-a-b, I_CL097, I_CL093-a, and II_CL079-a) contained only environmental isolates from unknown sources, three (III_CL006-a, I_CL024-a, and I_CL105-a) contained only environmental isolates from bovine sources, two (I_CL066-b and II_CL032-a) contained only environmental isolates from water, soil, and sediment samples, and two (I_CL008-p and I_CL021-n) contained only environmental isolates from produce environments. One molecular cluster (I_CL008-d) predominantly comprised environmental isolates (133 of the 143 isolates); notably, most (*n* = 109) of these nonclinical isolates were from produce environments (Table S5).

Moreover, our SNP-based analysis identified one molecular epidemiological cluster containing an isolate that could be linked to a known national outbreak (the isolate had an Outbreak ID available in its metadata on NCBI). Cluster III_CL006_CL011_CL019-f contained isolate GCA_010645465.1, which was isolated from fresh jalapeno pepper from Mexico and had an Outbreak ID listed as 0808CAJBP-1c. This cluster contained 24 total isolates, but this was the only one with that outbreak ID listed. Additionally, it was the only isolate on the pathogen browser with that outbreak ID. Other isolates contained in this molecular cluster were clinical isolates from the United States, and one other environmental isolate is from sediment from West Virginia, USA. Further investigation into that isolate GCA_010645465.1 on the NCBI Pathogen Browser showed that it belonged to SNP cluster PDS000004004.172.

### Predicted resistance to some antibiotics differs between sources, locations, and clades

We performed antibiotic resistance analysis to identify the presence of genes that may confer resistance to 86 antibiotics, belonging to a total of 20 antibiotic classes, in all *S. enterica* ser. Braenderup genomes (*n* = 6,536) used in the present study. Furthermore, our analysis indicated that the isolates from southeastern USA and the United States showed the highest predicted antimicrobial resistance levels toward beta-lactams (2.43% and 2.44%, respectively), tetracyclines (2.32% and 2.17%, respectively), folate pathway antagonists (2.22% and 1.43%, respectively), aminoglycosides (2.00% and 2.15%, respectively), and quinolones (2.00% and 2.06%, respectively) (Table S6).

Statistical analyses revealed that clade had a significant effect on predicted antimicrobial resistance for five of the antibiotic classes. We also found that predicted resistance toward beta-lactam antibiotics (including amoxicillin and ampicillin, which are commonly used to treat *Salmonella*) was significantly higher in Clade II (3.67%) than in both Clade I (1.86%; *P* < 0.0001) and Clade Group III (1.03%; *P* = 0.0029). The predicted resistance to ciprofloxacin, a quinolone antibiotic used to treat *Salmonella*, was significantly higher in Clade Group III (9.97%) compared to Clade I (4.34%) (*P* < 0.0001) and Clade II (0.93%) (*P* < 0.0001). We also found that Clade I had a significantly higher resistance than Clade II (*P* < 0.0001, indicating a notable variation in quinolone resistance patterns across both the clades and clade group. Additionally, predicted resistance to folate pathway antagonists was significantly higher (*P* = 0.0110) in Clade II (2.21%) than in Clade I (1.22%). The effect of clade on predicted resistance was also significant for aminocyclitols (*P* = 0.0036) and tetracyclines (*P* < 0.0001), although antibiotics from these classes are not typically used to treat salmonellosis (Table S6).

Our findings also showed that isolation sources had a significant effect on predicted resistance to three antibiotic classes: quinolones (*P* = 0.0167), aminoglycosides (*P* = 0.0065), and fosfomycin (*P* = 0.0034). Predicted resistance to quinolones, which includes an antibiotic used to treat salmonellosis, was significantly higher (*P* = 0.0139) in clinical isolates (4.03%) than in environmental/other isolates (2.15%).

## DISCUSSION

In this study, we analyzed a large data set of clinical and nonclinical *S. enterica* ser. Braenderup isolate genomes sourced from southeastern USA, other USA regions, other North American countries, South America, Europe, Africa, and Asia. There is an observed disparity in the geographical distribution among the clades, which could be attributed to the geographical bias in the data set. The data set contained of a larger number of isolates from regions with more established public health surveillance networks (i.e., North America and Europe), which may result in an underrepresentation of isolates from other regions (93). Additionally, participating states in the current study provided isolate identifiers, so the specific state of isolation was known for each isolate. In contrast, our data set from other USA regions, excluding southeastern USA, lacked state-specific information. The global data set included available NCBI metadata, so many USA isolates lacked state of isolation information and other important metadata, which limited our ability to conduct a comparative analysis at the same level of detail as with the southeastern USA data set. Our analyses enabled (i) extensive examination of the population structure of *S. enterica* ser. Braenderup, confirming that it is monophyletic, (ii) identification of genomic clades and potential source attribution trends for *S. enterica* ser. Braenderup, (iii) determination of the fact that a large portion (43.9%) of clinical isolates from the United States were part of molecular epidemiological clusters, and (iv) identification of pronounced predicted resistance patterns among USA isolates compared to isolates from global locations to important antibiotic classes, including beta-lactams, and folate pathway antagonists.

### The globally diverse population structure of *S. enterica* ser. Braenderup confirms it is monophyletic

Previous studies have found that *S*. *enterica* serovars can be predicted from the legacy multilocus sequence typing (MLST) eBurst groups (eBGs) (94, 95). Previous studies have found that cgMLST eBurst groups (ceBGs) are equivalent to eBGs from 7-gene MLST and that these ceBGs correspond to serovar designations (69, 90, 94). Moreover, it is known that the monophyletic serovars belong to a single eBG, while polyphyletic serovars will belong to two or more (7, 89, 95, 96). Hudson et al. (89) reported the branch lengths between ceBG clusters for some established polyphyletic serovars ranging from 929 to 2,850 allelic differences, which is longer than the longest branch length between two *S*. *enterica* ser. Braenderup isolates; therefore, it is reasonable to infer that *S. enterica* ser. Braenderup exhibits a monophyletic population structure. There has been limited research published on the population structure of this serovar. In our study, the global population structure of *S. enterica* ser. Braenderup, along with reference strains representing 407 other S. enterica serovars, revealed that all the *S. enterica* ser. Braenderup strains examined belonged to a single ceBG (ceBG 185). Previous studies on the phylogeny of *S. enterica* that have included *S. enterica* ser. Braenderup show results consistent with it being a monophyletic serovar (5–7, 97). Yin et al. (5) analyzed 4,498 *Salmonella* strains using MLST phylogeny and found that all included *S. enterica* ser. Braenderup strains (*n* = 8) were associated with a single eBurst group (eBG 24), which indicates that Braenderup is monophyletic. Alikhan et al*.* (7) evaluated over 100,000 *Salmonella* strains and found only one rMLST eBurst group (reBG 24) associated with the included *S. enterica* ser. Braenderup strains (*n* = 1,582), which is indicative of monophylogeny. Additionally, Elnekave et al*.* (97) examined the phylogeny of 37 *Salmonella* serovars (10 representative genomes for each) using core-genome SNPs and found that all ten *S. enterica* ser. Braenderup strains formed a monophyletic clade. Liu et al. (6) evaluated over 180,098 *Salmonella* genomes and found all the *S. enterica* ser. Braenderup isolates (*n* = 3,298) were mapped to one specific genomic cluster (cluster 53), which is indicative of single lineage, i.e., monophylogeny. Our results from the population structure analysis support the previous studies’ findings. However, in those previous studies, some only included a limited number of *S. enterica* ser. Braenderup strains and were not focused specifically on this serovar. By contrast, our study has included an extensively large data set of all *S. enterica* ser. Braenderup genomes available on EnteroBase from global sources for inferring evolutionary history.

### Transmission pathways may be clade-specific

Other studies have found specific clades of *S. enterica* serovars (98–101) and other foodborne pathogens (102) to be associated with different environments or reservoirs, which can be used to predict outbreak sources and transmission routes. Based on previous studies, *S. enterica* ser. Braenderup has been linked with numerous transmission routes, including sources like fresh produce, animal/animal foods, environmental facilities and farms, and water (15–18, 29, 31, 33, 34, 38, 39). Our results indicate that transmission pathways for *S. enterica* ser. Braenderup may be clade-specific: Clade I was associated primarily with produce and produce-associated environments, like water, soil, and sediment, and Clade II was primarily associated with poultry environments. The clade-specific gene associations identified in this study can provide valuable insights into potential mechanisms underlying environmental adaptation and host–pathogen interaction.

In our study, all three clades included isolates sourced from poultry environments, with Clade II showing a higher proportion of environmental isolates from poultry and poultry-related environments (62.8%; *P* < 0.0001) as compared to Clade I (37.1%; *P* < 0.0001%). This is consistent with previous work showing an association between *S. enterica* ser. Braenderup and poultry, poultry products, and poultry environments (29, 103, 104). This strong connection underscores the important role of poultry as a natural reservoir for *S. enterica* serovars, including Braenderup (34, 87). Numerous human salmonellosis outbreaks have been linked to poultry and eggs (29), including at least 27 caused by *S. enterica* ser. Braenderup (103, 105), which emphasizes the importance of understanding these environmental associations. Our findings support that poultry is a prominent source of *S. enterica* ser. Braenderup and that specific clades may be better adapted to poultry and poultry environments. Clade II was enriched with genes encoding heme-exporter proteins and dimethyl sulfoxide reductase, which can facilitate iron acquisition and anaerobic respiration, supporting *Salmonella* growth and persistence in iron-rich, low-oxygen conditions commonly found in poultry environments (106–109). Notably, isolates from food animal sources were likely overrepresented in this study compared to non-animal environmental sources, due to the more frequent sampling and monitoring required by regulatory agencies in poultry and other food animal environments (110, 111).

Additionally, Clade I was strongly associated with produce environments (90.7%; *P* < 0.0001%) and environments relevant to produce such as water, soil, and sediment (76.9%; *P* < 0.0001%). This aligns with the observations of previous studies that identified *S. enterica* ser. Braenderup among the top 15 *Salmonella* serotypes isolated from human patients who consumed contaminated produce (112) and found that this serovar was predominantly linked with vegetable outbreaks between 1998 and 2008 (113). Produce-related *S. enterica* ser. Braenderup outbreaks have been associated with Roma tomatoes (42), iceberg lettuce (15), melons (40, 41), and mangoes (16). However, we cannot conclude whether the isolates associated with fresh produce are adapted to these environments or whether the observed associations are more reflective of the origins of contamination or attenuated virulence.

Isolate sources categorized as produce and water, soil, and sediment have overlap in agricultural production systems and may serve as potential contamination sources through growing and harvesting practices such as irrigation, washing, and soil cultivation (114, 115). Previous studies have detected *S. enterica* ser. Braenderup in various aquatic environments, including watersheds and surface waters in the southeastern USA (33, 38), creek water (116), irrigation water, soil, and sediment (39, 117). In 2021, a *S. enterica* ser. Braenderup outbreak was linked to imported melons from Honduras, and the outbreak strain was isolated from the surface of a washing tank at one of the melon packing facilities (40, 41). Kumar et al. (117) showed that *S. enterica* ser. Braenderup was able to persist in soil and water and able to form biofilms and attach to surfaces, further supporting that this serovar can contaminate and survive in produce-related environments. Another source of produce contamination is through domesticated or wild animal fecal contamination of produce environments or water sources (22, 114, 118–120).

While Clade I shows an association with produce-related environments, it is unclear whether this association is more transient or permanent due to the complex nature of produce contamination (120). One possible explanation is that isolates in this clade are better adapted to survive and persist in produce-related environments. Certain environmental factors, such as soil moisture, temperature, and proximity to water bodies or urban areas, can influence the persistence of foodborne pathogens, including *Salmonella*, in produce fields (115, 121). Thus, it is possible that this clade is better adapted to some of these produce field-relevant stressors. Clade I was enriched with genes encoding Competence protein EC (ComEC) and Cytochrome_c_Asm, which may facilitate DNA uptake and transformation (122–128). These functions can support bacterial survival, adaptation, and virulence under anoxic or nutrient-limited conditions commonly found in agricultural environments, including soil, sediment, and water environments (122–126). These Clade I-enriched genes may play a role in adaptation to these environments, warranting further investigation. Alternatively, it is possible that produce-related environments are transient habitats for these isolates and that they are actually associated with other specific reservoirs (e.g., wild animals) that can contaminate produce-related environments (119, 129). Unfortunately, the amount of available isolates from wild animals (only 36 isolates in this study) is not large enough to draw any conclusions about potential original sources in this produce environment-related clade. While produce environments may indeed represent transient habitats for *Salmonella*, the risk of contamination remains significant due to multiple potential exposure routes, including contaminated agricultural water, soil, and manure. The clade-specific environmental associations identified in this study can be used by public health professionals to guide case questionnaires about specific *a priori* hypotheses and exposures, narrow down likely sources for outbreaks, and develop targeted prevention strategies.

### Over one-third of *S. enterica* ser. Braenderup clinical isolates from the United States may be outbreak-associated

In the present study, we identified 218 molecular epidemiological clusters using an initial distance threshold of 15 SNPs, followed by an individual clade distance threshold of ten hqSNPs. Notably, we found that 43.9% of clinical USA isolates (including southeastern USA) were a part of molecular epidemiological clusters. For comparison, FoodNet Fast was queried for culture-confirmed *S. enterica* ser. Braenderup infections between 2017 and 2022; of the 837 infections, only 15% were outbreak-associated (130), which is less than half the proportion found to belong to molecular epidemiological clusters in the present study. FoodNet defines an outbreak as an event where two or more cases of similar illness are associated with a common exposure, although some state public health jurisdictions in the United States also stipulate that these illnesses should be from more than one household. Further, it states that an unknown outbreak association status is classified as not outbreak-associated (131). The discrepancy between our study findings and FoodNet may stem from FoodNet’s data collection scope and surveillance area, which is limited to ten USA states, including some states not fully covered at the county level, and represents just 15% of the entire USA population (132). In contrast, our study encompasses isolates from all USA states available on the NCBI.

Our study identified molecular epidemiological clusters based on SNP thresholds, which may detect continuous common-source outbreaks over extended periods. In contrast, the current PulseNet guidelines for local cluster detection for *Salmonella* are more stringent, requiring three or more cases differing within ten alleles by cgMLST, of which two should differ within five alleles, and isolation dates for all cases should be within the past 60 days of detection (90). However, an important limitation in our study is that we did not incorporate isolation dates and additional epidemiological data for cluster confirmation, which public health departments typically use. We excluded these additional criteria due to challenges in aquiring the data from multiple sources and also because our aim was to capture molecular continuous common source outbreak clusters that spanned over a longer period of time to understand the serovar more holistically. Additionally, our study did not consider epidemiological or traceback data, including household cases. Since household clusters are not classified as outbreaks, our broader criteria may have contributed to the higher proportion of putative outbreak clusters identified. Nonetheless, these data indicate that some clusters and outbreaks may not be detected by public health professionals with current cluster definitions and approaches to outbreak detection.

Although we report here a higher percentage of isolates putatively associated with outbreaks than reported by FoodNet, if we were to reduce the SNP threshold (e.g., from 10 to 5 SNPs), these percentages may become closer aligned. This highlights the importance of previous work that described uncertainty and variability in using a strict SNP cut-off to determine closely related cases among foodborne pathogens as these can be influenced by analyses/pipelines and genomic coverage (133). Another study by Stimson et al. (134) concluded that while SNP thresholds are commonly used to identify outbreak clusters, there is little consensus on the exact threshold. Also, Bergholz et al. (135) stated the importance of employing micro-evolutionary analyses to determine the recent common ancestor of closely related isolates; epidemiological data can then be used to identify meaningful clusters. Even though different species or serovars may require different distance thresholds for accurate epidemiological cluster detection, it remains common to employ a singular SNP threshold for a particular group of pathogens (e.g., a singular threshold for all *Campylobacter* species or a singular threshold for all *S. enterica,* regardless of serovars) to identify clusters. This highlights the potential need to define *Salmonella enterica* serovar-specific thresholds to improve the accuracy of cluster detection.

### For some antibiotics, the proportion of clinical isolates from the United States with predicted resistance was lower than reported by NARMS

The global challenge of antibiotic resistance has led to the emergence of pathogens that can withstand important antibiotics, thus making the surveillance of resistance determinants in *Salmonella* isolates crucial. In the United States, such resistance leads to roughly 2 million infections and 23,000 fatalities each year, contributing an additional $20 billion to healthcare expenses and a $35 billion loss in productivity (136). The CDC lists the following as essential antibiotics for nontyphoidal *Salmonella*: ciprofloxacin, azithromycin, ceftriaxone, ampicillin, and trimethoprim-sulfamethoxazole (57). There are some antibiotics, including ciprofloxacin (quinolone), azithromycin (macrolide), ceftriaxone (cephalosporin/beta-lactam), ampicillin (beta-lactam), amoxicillin (beta-lactam), chloramphenicol (amphenicol), and trimethoprim-sulfamethoxazole (folate pathway antagonist) that are used to treat severe salmonellosis (137, 138). Isolates from the southeastern USA (2.0%), USA (2.06%), and global locations (10.49%) showed predicted resistance to ciprofloxacin (quinolone). This finding is notable as quinolones represent a critical class of antibiotics extensively used to treat invasive bacterial illnesses (137, 139) and quinolone-resistant strains of *Salmonella* have been classified as a high-priority pathogen by the World Health Organization (WHO) (137, 140). However, the extensive use of these antibiotics to treat *Salmonella* has led to an increase in the resistance rates (141, 142). Given this challenge, the World Health Organization has highlighted the critical need for developing new antibiotics to treat salmonellosis effectively (141, 143, 144).

Additionally, we compared the predicted antibiotic resistance patterns for the isolates analyzed in the present study (*n* = 6,536) with National Antimicrobial Resistance Monitoring System (NARMS) human clinical data, which included predicted resistance to 24 antibiotic classes and 88 *S*. *enterica* ser. Braenderup isolates (Table S6). The predicted resistance levels of clinical isolates from the United States were lower (by 5 to 10 percentage points) than those reported by NARMS for 29 antibiotics (Table S6). Hudson et al. (90) posited that these discrepancies might be due to the differences in the ResFinder and NARMS databases used to predict antibiotic resistance. This discrepancy carries a significant implication for local, state, and federal public health as enhanced collaboration and sharing of data between NARMS and other ABR databases (like ResFinder) could lead to a more accurate and comprehensive understanding of resistance trends, therefore facilitating more effective public health monitoring and response.

### Conclusions

This study advances our understanding of the diversity and transmission of the clinically significant *S. enterica* ser. Braenderup, as well as its genetic potential to be resistant to antimicrobial treatments. Our findings underscore the importance of subtyping isolates into distinct clades of *S. enterica* ser. Braenderup to potentially inform and enhance public health response. By identifying different clades of this serovar that show a higher association with specific environments, we can improve our knowledge of their potential origins and transmission pathways. For example, Clade I predominantly contained environmental isolates from produce environments, whereas Clades II and III predominantly contained environmental isolates from poultry environments.

Adopting a One-Health approach, which integrates data from human health, animal health, and environmental monitoring, could improve the management and prevention of *S. enterica* ser. Braenderup outbreaks. Recognizing the interconnectedness of these sectors and leveraging the findings in this study, such as the clade-specific associations with produce and poultry environments, we can better identify outbreak sources and transmission routes, facilitating faster responses and more effective mitigation strategies. This integrated and targeted approach would enhance surveillance, enable targeted interventions, and support more effective antimicrobial resistance (AMR) management by addressing the emergence and spread of resistant strains across human, animal, and environmental sources.

Future work should aim to expand the population structure with data sets that offer broader global representation, further investigate source attribution trends and their connection to genomic content, and assess how these findings can be incorporated into public health practice. Additionally, future work should also focus on integrating spatial and temporal data, which would further enhance our understanding of the epidemiological features of this serovar.

## Data Availability

All the isolate reads and/or assemblies are available from the NCBI SRA, and accession IDs are provided in Data Sets S1 and S2 in the supplemental material. EnteroBase genomes are available from EnteroBase, and the relevant IDs are provided in Data Set S3.

## SUPPLEMENTAL MATERIAL

Fig. S1 aem.02594-24-s0001.pdf Phylogenetic tree of Clade I isolates with labeled isolate IDs.

Fig. S2 aem.02594-24-s0002.pdf Phylogenetic tree of Clade II isolates with labeled isolate IDs.

Fig. S3 aem.02594-24-s0003.pdf Phylogenetic tree of Clade Group III isolates with labeled isolate IDs.

Fig. S4-S6 aem.02594-24-s0004.pdf Phylogeny, source, location, and potential epidemiological clusters.

Supplemental tables aem.02594-24-s0005.xlsx Tables S1 to S8.

ASM does not own the copyrights to Supplemental Material that may be linked to, or accessed through, an article. The authors have granted ASM a non-exclusive, world-wide license to publish the Supplemental Material files. Please contact the corresponding author directly for reuse.
