# Supplementary figures and images for: Salmonella enterica serovar Braenderup shows clade-specific source associations and a high proportion of molecular epidemiological clustering
Source: Appl Environ Microbiol. 2025 Mar 21;91(4):e02594-24. doi: 10.1128/aem.02594-24 (PMC12016519; doi:10.1128/aem.02594-24)

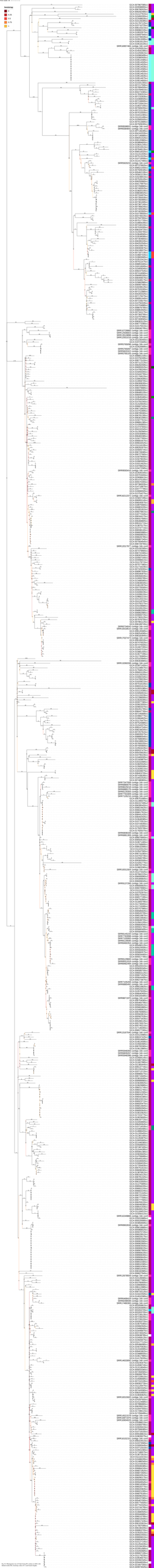

Supplement: Fig. S3 — Phylogenetic tree of Clade Group III isolates with labeled isolate IDs. [file aem.02594-24-s0003.pdf]
