# Supplementary material for: Salmonella enterica serovar Braenderup shows clade-specific source associations and a high proportion of molecular epidemiological clustering
Source: Appl Environ Microbiol. 2025 Mar 21;91(4):e02594-24. doi: 10.1128/aem.02594-24 (PMC12016519; doi:10.1128/aem.02594-24)
Supplement: Fig. S4-S6 — Phylogeny, source, location, and potential epidemiological clusters. [file aem.02594-24-s0004.pdf]

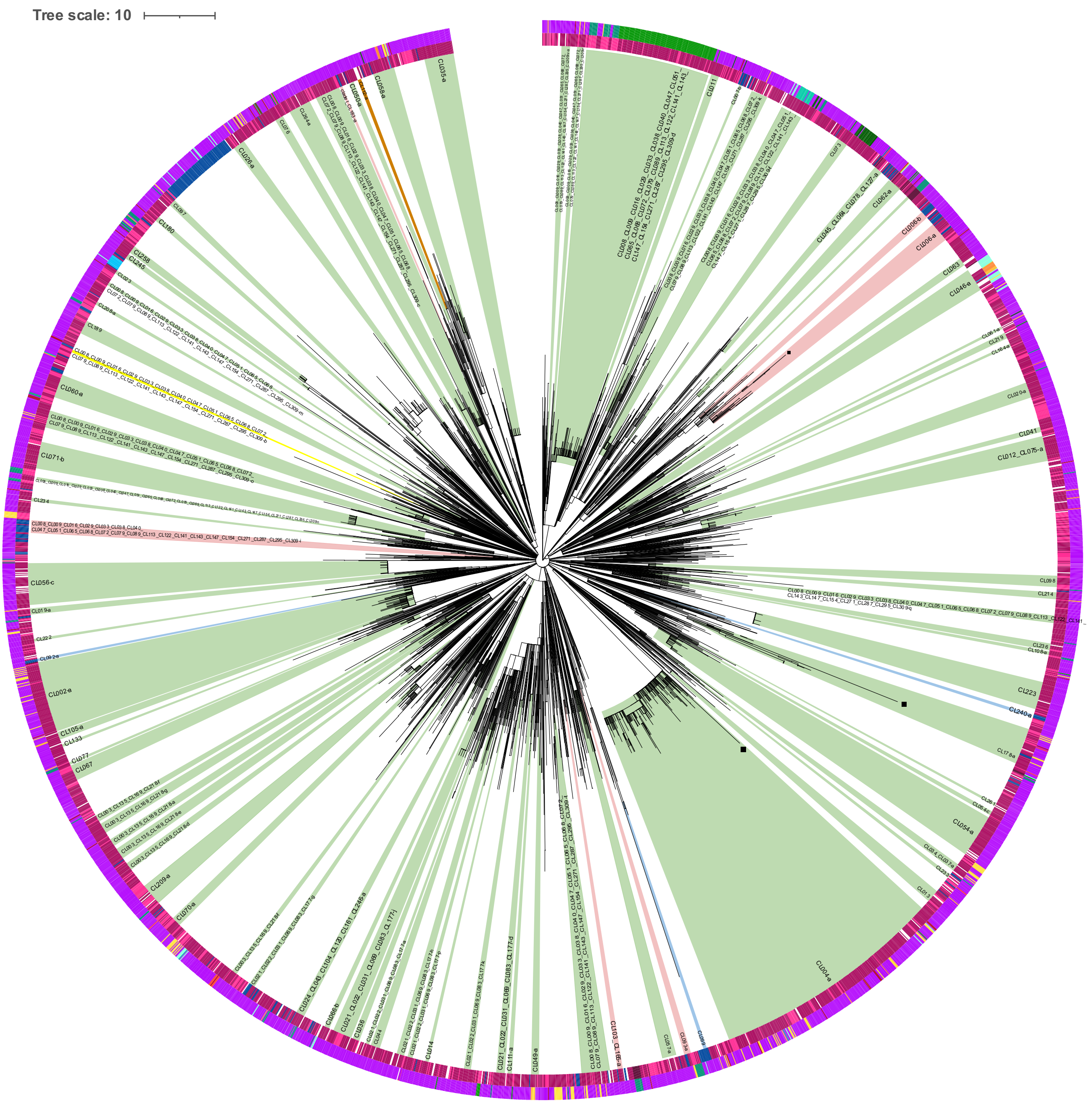

Fig. S4. Phylogeny, source, location, and potential epidemiological clusters for S. Braenderup isolates in Clade I. Neighbor-joining phylogenetic trees. Each tree is marked with a geography label (inner ring) and source attribution (outer ring) for all the isolates on the trees. The colored ranges depict labeled molecular epidemiological clusters in each clade. ■ Symbol denotes the isolate is not in the cluster. The trees were created in Mega X, annotated, and edited with iTOL.

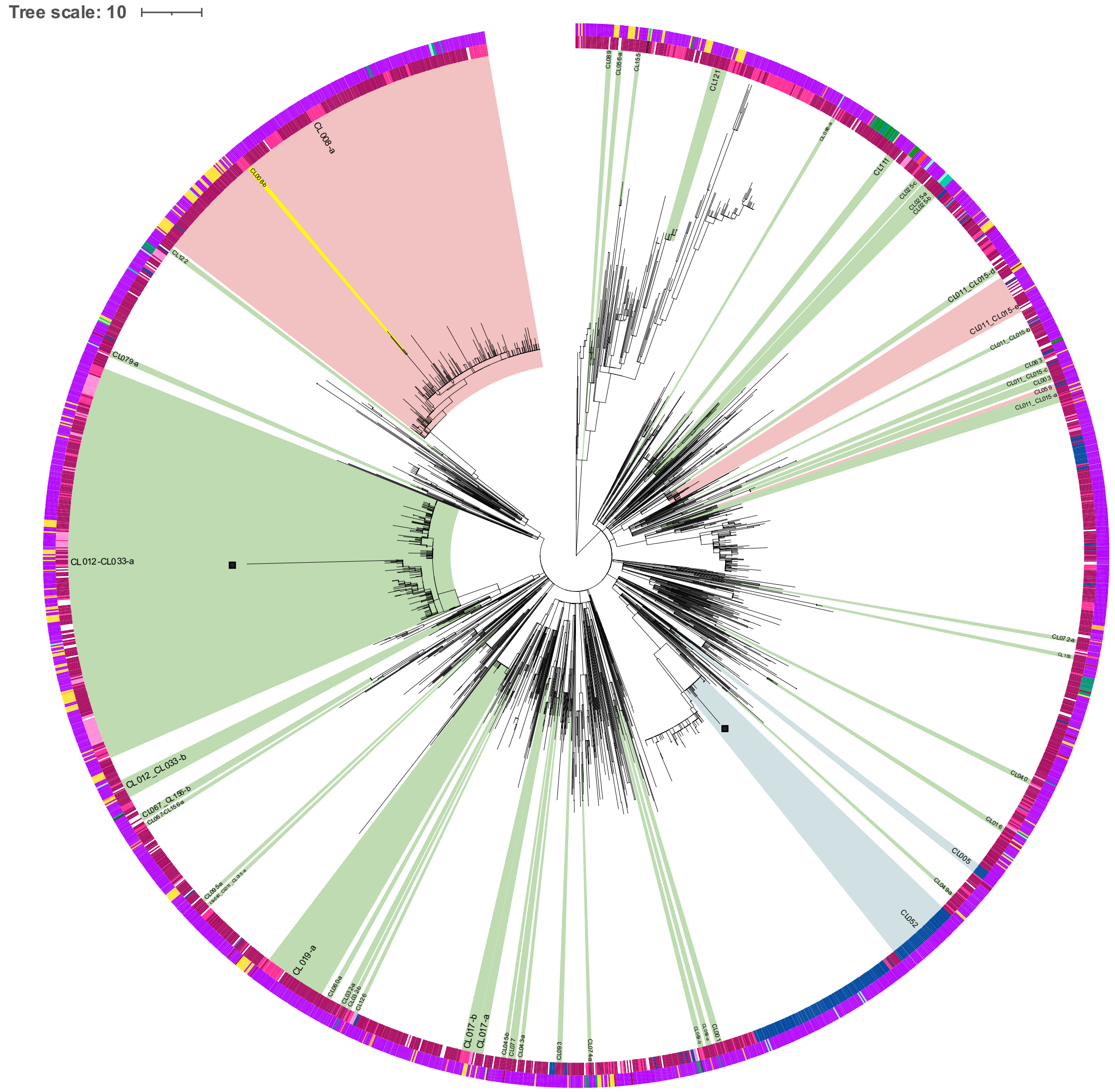

Fig. S5. Phylogeny, source, location, and potential epidemiological clusters for S. Braenderup isolates in Clade II. Neighbor-joining phylogenetic trees. Each tree is marked with a geography label (inner ring) and source attribution (outer ring) for all the isolates on the trees. The colored ranges depict labeled molecular epidemiological clusters in each clade. ■ Symbol denotes the isolate is not in the cluster. The trees were created in Mega X, annotated, and edited with iTOL.

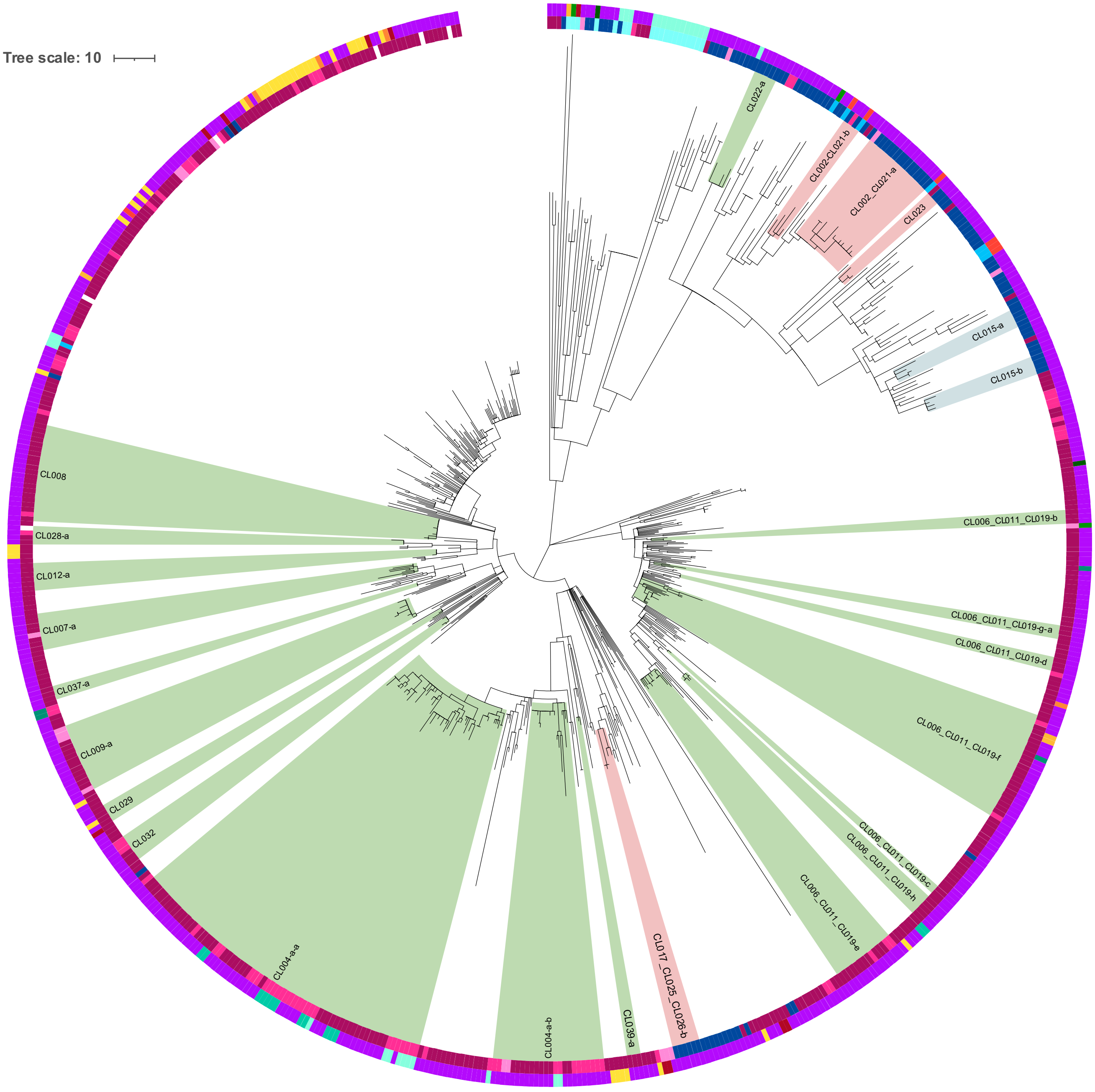

Fig. S6. Phylogeny, source, location, and potential epidemiological clusters for *S.* Braenderup isolates in Clade Group III. Neighbor-joining phylogenetic trees. Each tree is marked with a geography label (inner ring) and source attribution (outer ring) for all the isolates on the trees. The colored ranges depict labeled molecular epidemiological clusters in each clade. ■ Symbol denotes the isolate is not in the cluster. The trees were created in Mega X, annotated, and edited with iTOL.
