# Supplementary material for: Salmonella enterica serovar Braenderup shows clade-specific source associations and a high proportion of molecular epidemiological clustering
Source: Appl Environ Microbiol. 2025 Mar 21;91(4):e02594-24. doi: 10.1128/aem.02594-24 (PMC12016519; doi:10.1128/aem.02594-24)
Supplement: Fig. S1 — Phylogenetic tree of Clade I isolates with labeled isolate IDs. [file aem.02594-24-s0001.pdf]

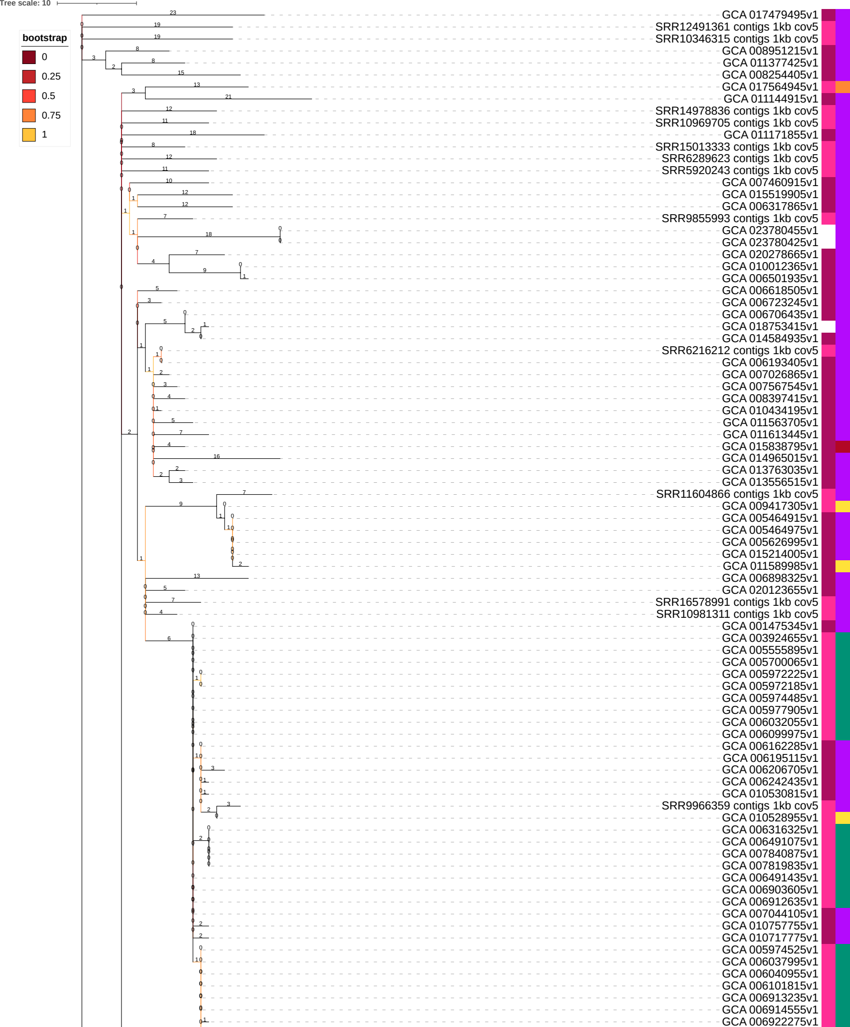
Tree scale: 10
bootstrap
0
0.25
0.5
0.75
1
GCA 017479495v1
SRR12491361 contigs 1kb cov5
SRR10346315 contigs 1kb cov5
GCA 008951215v1
GCA 011377425v1
GCA 008254405v1
GCA 017564945v1
GCA 011144915v1
SRR14978836 contigs 1kb cov5
SRR10969705 contigs 1kb cov5
GCA 011171855v1
SRR15013333 contigs 1kb cov5
SRR6289623 contigs 1kb cov5
SRR5920243 contigs 1kb cov5
GCA 007460915v1
GCA 015519905v1
GCA 006317865v1
SRR9855993 contigs 1kb cov5
GCA 023780455v1
GCA 023780425v1
GCA 020278665v1
GCA 010012365v1
GCA 006501935v1
GCA 006618505v1
GCA 006723245v1
GCA 006706435v1
GCA 018753415v1
GCA 014584935v1
SRR6216212 contigs 1kb cov5
GCA 006193405v1
GCA 007026865v1
GCA 007567545v1
GCA 008397415v1
GCA 010434195v1
GCA 011563705v1
GCA 011613445v1
GCA 015838795v1
GCA 014965015v1
GCA 013763035v1
GCA 013556515v1
SRR11604866 contigs 1kb cov5
GCA 009417305v1
GCA 005464915v1
GCA 005464975v1
GCA 005626995v1
GCA 015214005v1
GCA 011589985v1
GCA 006898325v1
GCA 020123655v1
SRR16578991 contigs 1kb cov5
SRR10981311 contigs 1kb cov5
GCA 001475345v1
GCA 003924655v1
GCA 005555895v1
GCA 005700065v1
GCA 005972225v1
GCA 005972185v1
GCA 005974485v1
GCA 005977905v1
GCA 006032055v1
GCA 006099975v1
GCA 006162285v1
GCA 006195115v1
GCA 006206705v1
GCA 006242435v1
GCA 010530815v1
SRR9966359 contigs 1kb cov5
GCA 010528955v1
GCA 006316325v1
GCA 006491075v1
GCA 007840875v1
GCA 007819835v1
GCA 006491435v1
GCA 006903605v1
GCA 006912635v1
GCA 007044105v1
GCA 010757755v1
GCA 010717775v1
GCA 005974525v1
GCA 006037995v1
GCA 006040955v1
GCA 006101815v1
GCA 006913235v1
GCA 006914555v1
GCA 006922275v1

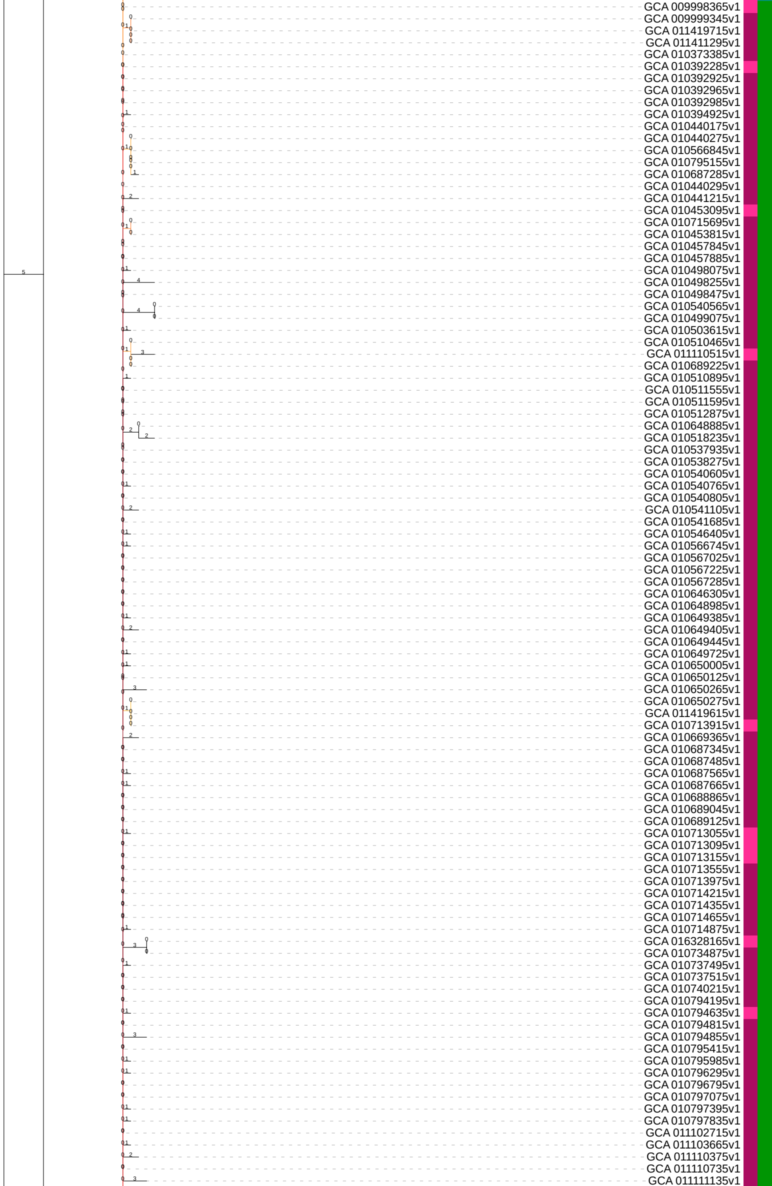

GCA 009998365v1
GCA 009999345v1
GCA 011419715v1
GCA 011411295v1
GCA 010373385v1
GCA 010392285v1
GCA 010392925v1
GCA 010392965v1
GCA 010392985v1
GCA 010394925v1
GCA 010440175v1
GCA 010440275v1
GCA 010566845v1
GCA 010795155v1
GCA 010687285v1
GCA 010440295v1
GCA 010441215v1
GCA 010453095v1
GCA 010715695v1
GCA 010453815v1
GCA 010457845v1
GCA 010457885v1
GCA 010498075v1
GCA 010498255v1
GCA 010498475v1
GCA 010540565v1
GCA 010499075v1
GCA 010503615v1
GCA 010510465v1
GCA 011110515v1
GCA 010689225v1
GCA 010510895v1
GCA 010511555v1
GCA 010511595v1
GCA 010512875v1
GCA 010648885v1
GCA 010518235v1
GCA 010537935v1
GCA 010538275v1
GCA 010540605v1
GCA 010540765v1
GCA 010540805v1
GCA 010541105v1
GCA 010541685v1
GCA 010546405v1
GCA 010566745v1
GCA 010567025v1
GCA 010567225v1
GCA 010567285v1
GCA 010646305v1
GCA 010648985v1
GCA 010649385v1
GCA 010649405v1
GCA 010649445v1
GCA 010649725v1
GCA 010650005v1
GCA 010650125v1
GCA 010650265v1
GCA 010650275v1
GCA 011419615v1
GCA 010713915v1
GCA 010669365v1
GCA 010687345v1
GCA 010687485v1
GCA 010687565v1
GCA 010687665v1
GCA 010688865v1
GCA 010689045v1
GCA 010689125v1
GCA 010713055v1
GCA 010713095v1
GCA 010713155v1
GCA 010713555v1
GCA 010713975v1
GCA 010714215v1
GCA 010714355v1
GCA 010714655v1
GCA 010714875v1
GCA 016328165v1
GCA 010734875v1
GCA 010737495v1
GCA 010737515v1
GCA 010740215v1
GCA 010794195v1
GCA 010794635v1
GCA 010794815v1
GCA 010794855v1
GCA 010795415v1
GCA 010795985v1
GCA 010796295v1
GCA 010796795v1
GCA 010797075v1
GCA 010797395v1
GCA 010797835v1
GCA 011102715v1
GCA 011103665v1
GCA 011110375v1
GCA 011110735v1
GCA 011111135v1

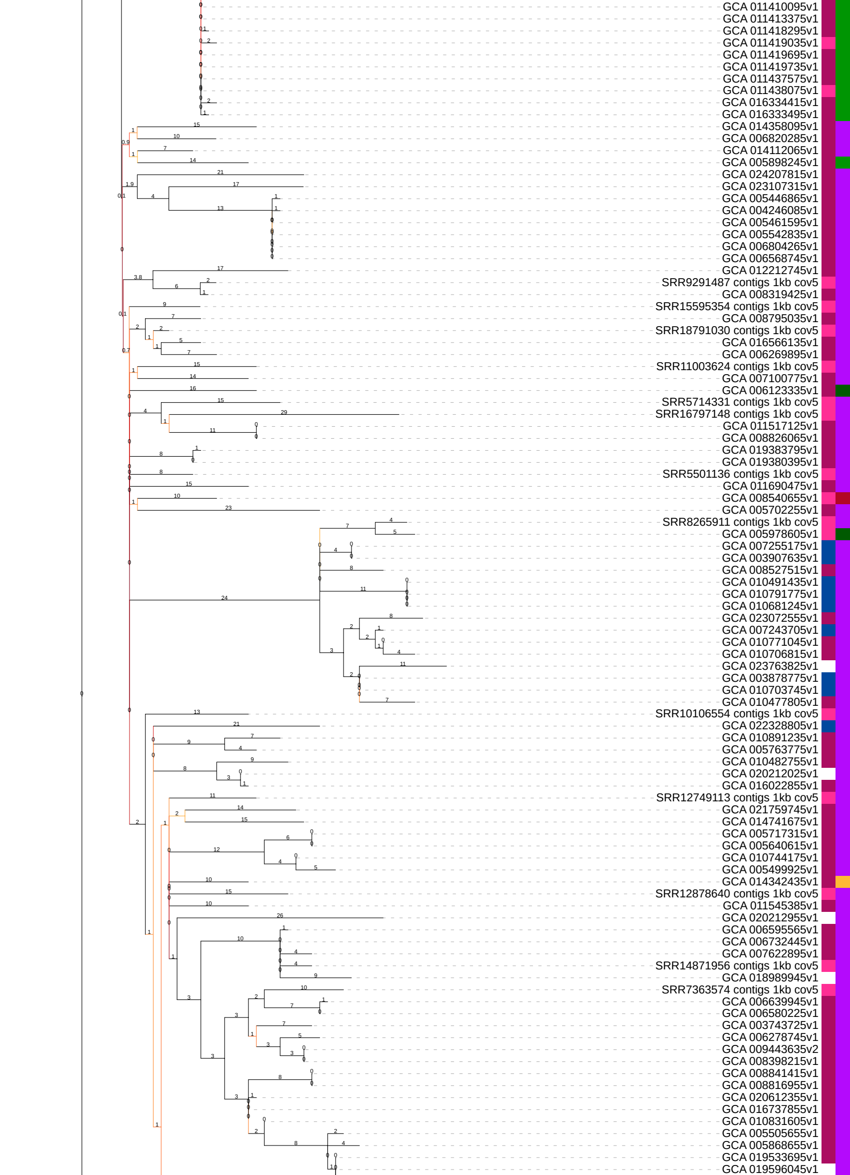
GCA 011410095v1
GCA 011413375v1
GCA 011418295v1
GCA 011419035v1
GCA 011419695v1
GCA 011419735v1
GCA 011437575v1
GCA 011438075v1
GCA 016334415v1
GCA 016333495v1
GCA 014358095v1
GCA 006820285v1
GCA 014112065v1
GCA 005898245v1
GCA 024207815v1
GCA 023107315v1
GCA 005446865v1
GCA 004246085v1
GCA 005461595v1
GCA 005542835v1
GCA 006804265v1
GCA 006568745v1
GCA 012212745v1
SRR9291487 contigs 1kb cov5
GCA 008319425v1
SRR15595354 contigs 1kb cov5
GCA 008795035v1
SRR18791030 contigs 1kb cov5
GCA 016566135v1
GCA 006269895v1
SRR11003624 contigs 1kb cov5
GCA 007100775v1
GCA 006123335v1
SRR5714331 contigs 1kb cov5
SRR16797148 contigs 1kb cov5
GCA 011517125v1
GCA 008826065v1
GCA 019383795v1
GCA 019380395v1
SRR5501136 contigs 1kb cov5
GCA 011690475v1
GCA 008540655v1
GCA 005702255v1
SRR8265911 contigs 1kb cov5
GCA 005978605v1
GCA 007255175v1
GCA 003907635v1
GCA 008527515v1
GCA 010491435v1
GCA 010791775v1
GCA 010681245v1
GCA 023072555v1
GCA 007243705v1
GCA 010771045v1
GCA 010706815v1
GCA 023763825v1
GCA 003878775v1
GCA 010703745v1
GCA 010477805v1
SRR10106554 contigs 1kb cov5
GCA 022328805v1
GCA 010891235v1
GCA 005763775v1
GCA 010482755v1
GCA 020212025v1
GCA 016022855v1
SRR12749113 contigs 1kb cov5
GCA 021759745v1
GCA 014741675v1
GCA 005717315v1
GCA 005640615v1
GCA 010744175v1
GCA 005499925v1
GCA 014342435v1
SRR12878640 contigs 1kb cov5
GCA 011545385v1
GCA 020212955v1
GCA 006595565v1
GCA 006732445v1
GCA 007622895v1
SRR14871956 contigs 1kb cov5
GCA 018989945v1
SRR7363574 contigs 1kb cov5
GCA 006639945v1
GCA 006580225v1
GCA 003743725v1
GCA 006278745v1
GCA 009443635v2
GCA 008398215v1
GCA 008841415v1
GCA 008816955v1
GCA 020612355v1
GCA 016737855v1
GCA 010831605v1
GCA 005505655v1
GCA 005868655v1
GCA 019533695v1
GCA 019596045v1

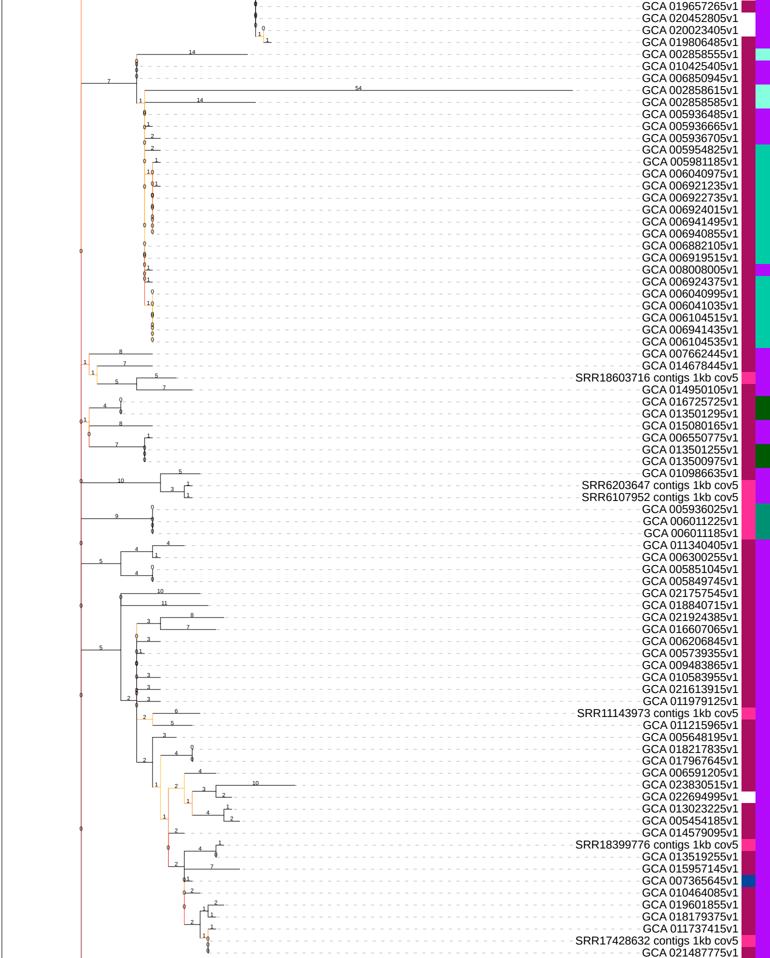
GCA 019657265v1
GCA 020452805v1
GCA 020023405v1
GCA 019806485v1
GCA 002858555v1
GCA 010425405v1
GCA 006850945v1
GCA 002858615v1
GCA 002858585v1
GCA 005936485v1
GCA 005936665v1
GCA 005936705v1
GCA 005954825v1
GCA 005981185v1
GCA 006040975v1
GCA 006921235v1
GCA 006922735v1
GCA 006924015v1
GCA 006941495v1
GCA 006940855v1
GCA 006882105v1
GCA 006919515v1
GCA 008008005v1
GCA 006924375v1
GCA 006040995v1
GCA 006041035v1
GCA 006104515v1
GCA 006941435v1
GCA 006104535v1
GCA 007662445v1
GCA 014678445v1
SRR18603716 contigs 1kb cov5
GCA 014950105v1
GCA 016725725v1
GCA 013501295v1
GCA 015080165v1
GCA 006550775v1
GCA 013501255v1
GCA 013500975v1
GCA 010986635v1
SRR6203647 contigs 1kb cov5
SRR6107952 contigs 1kb cov5
GCA 005936025v1
GCA 006011225v1
GCA 006011185v1
GCA 011340405v1
GCA 006300255v1
GCA 005851045v1
GCA 005849745v1
GCA 021757545v1
GCA 018840715v1
GCA 021924385v1
GCA 016607065v1
GCA 006206845v1
GCA 005739355v1
GCA 009483865v1
GCA 010583955v1
GCA 021613915v1
GCA 011979125v1
SRR11143973 contigs 1kb cov5
GCA 011215965v1
GCA 005648195v1
GCA 018217835v1
GCA 017967645v1
GCA 006591205v1
GCA 023830515v1
GCA 022694995v1
GCA 013023225v1
GCA 005454185v1
GCA 014579095v1
SRR18399776 contigs 1kb cov5
GCA 013519255v1
GCA 015957145v1
GCA 007365645v1
GCA 010464085v1
GCA 019601855v1
GCA 018179375v1
GCA 011737415v1
SRR17428632 contigs 1kb cov5
GCA 021487775v1

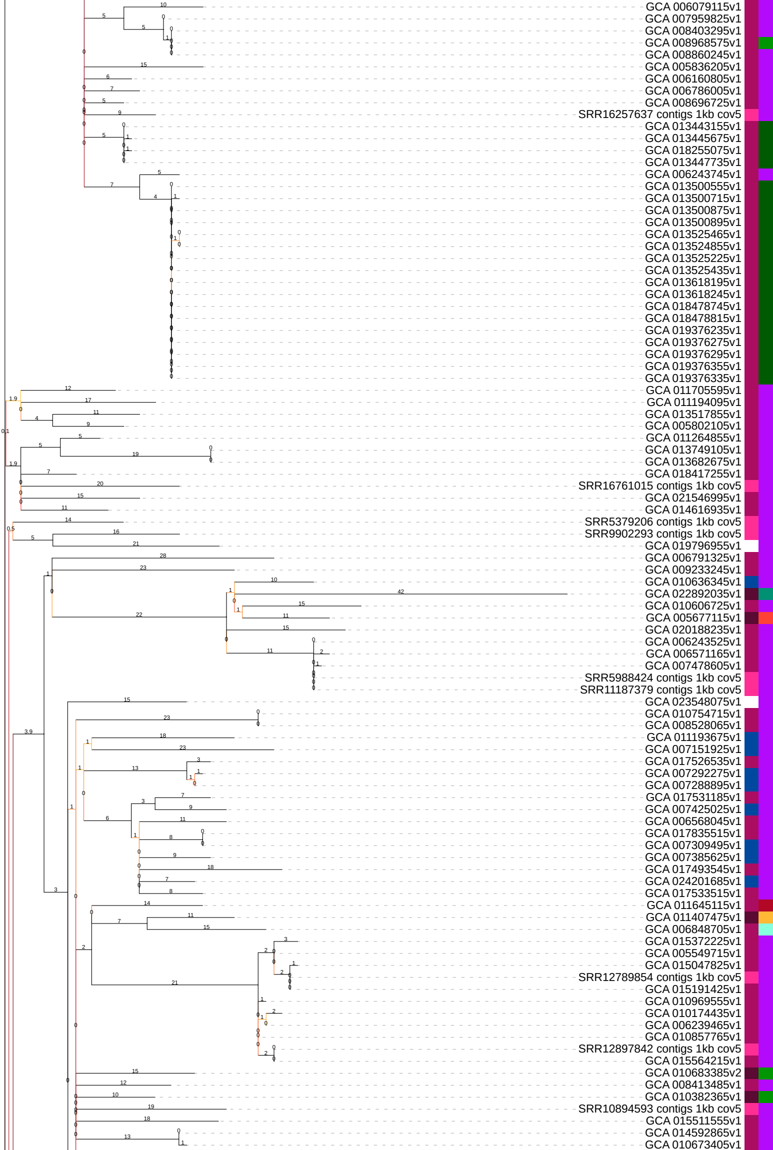
GCA 006079115v1
GCA 007959825v1
GCA 008403295v1
GCA 008968575v1
GCA 008860245v1
GCA 005836205v1
GCA 006160805v1
GCA 006786005v1
GCA 008696725v1
SRR16257637 contigs 1kb cov5
GCA 013443155v1
GCA 013445675v1
GCA 018255075v1
GCA 013447735v1
GCA 006243745v1
GCA 013500555v1
GCA 013500715v1
GCA 013500875v1
GCA 013500895v1
GCA 013525465v1
GCA 013524855v1
GCA 013525225v1
GCA 013525435v1
GCA 013618195v1
GCA 013618245v1
GCA 018478745v1
GCA 018478815v1
GCA 019376235v1
GCA 019376275v1
GCA 019376295v1
GCA 019376355v1
GCA 019376335v1
GCA 011705595v1
GCA 011194095v1
GCA 013517855v1
GCA 005802105v1
GCA 011264855v1
GCA 013749105v1
GCA 013682675v1
GCA 018417255v1
SRR16761015 contigs 1kb cov5
GCA 021546995v1
GCA 014616935v1
SRR5379206 contigs 1kb cov5
SRR9902293 contigs 1kb cov5
GCA 019796955v1
GCA 006791325v1
GCA 009233245v1
GCA 010636345v1
GCA 022892035v1
GCA 010606725v1
GCA 005677115v1
GCA 020188235v1
GCA 006243525v1
GCA 006571165v1
GCA 007478605v1
SRR5988424 contigs 1kb cov5
SRR11187379 contigs 1kb cov5
GCA 023548075v1
GCA 010754715v1
GCA 008528065v1
GCA 011193675v1
GCA 007151925v1
GCA 017526535v1
GCA 007292275v1
GCA 007288895v1
GCA 017531185v1
GCA 007425025v1
GCA 006568045v1
GCA 017835515v1
GCA 007309495v1
GCA 007385625v1
GCA 017493545v1
GCA 024201685v1
GCA 017533515v1
GCA 011645115v1
GCA 011407475v1
GCA 006848705v1
GCA 015372225v1
GCA 005549715v1
GCA 015047825v1
SRR12789854 contigs 1kb cov5
GCA 015191425v1
GCA 010969555v1
GCA 010174435v1
GCA 006239465v1
GCA 010857765v1
SRR12897842 contigs 1kb cov5
GCA 015564215v1
GCA 010683385v2
GCA 008413485v1
GCA 010382365v1
SRR10894593 contigs 1kb cov5
GCA 015511555v1
GCA 014592865v1
GCA 010673405v1

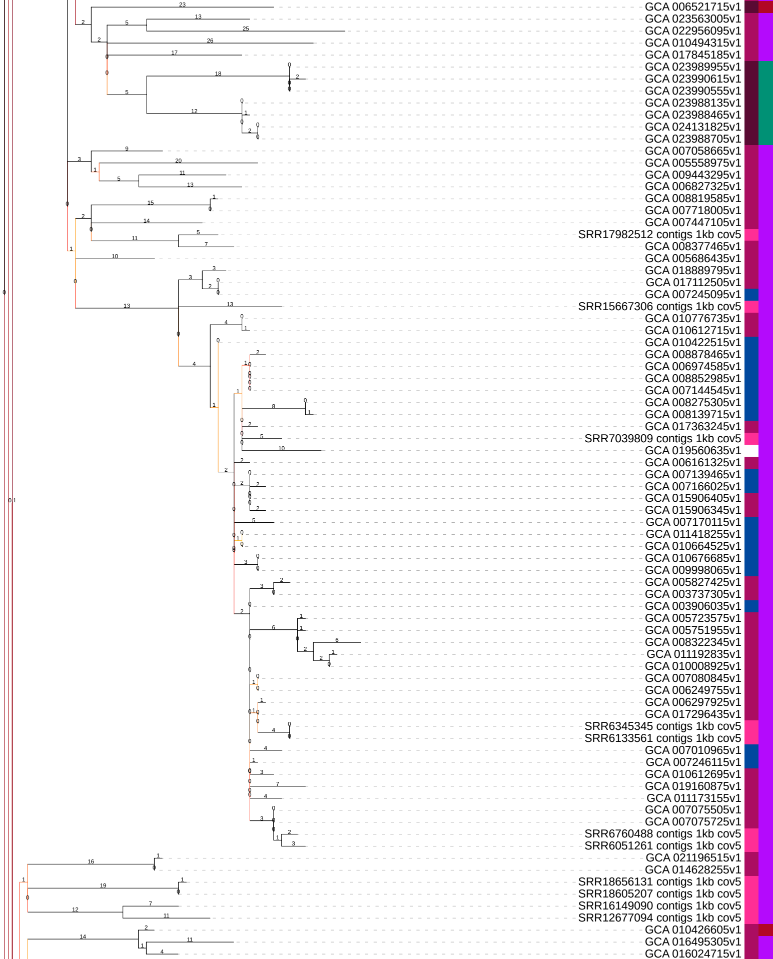
GCA 006521715v1
GCA 023563005v1
GCA 022956095v1
GCA 010494315v1
GCA 017845185v1
GCA 023989955v1
GCA 023990615v1
GCA 023990555v1
GCA 023988135v1
GCA 023988465v1
GCA 024131825v1
GCA 023988705v1
GCA 007058665v1
GCA 005558975v1
GCA 009443295v1
GCA 006827325v1
GCA 008819585v1
GCA 007718005v1
GCA 007447105v1
SRR17982512 contigs 1kb cov5
GCA 008377465v1
GCA 005686435v1
GCA 018889795v1
GCA 017112505v1
GCA 007245095v1
SRR15667306 contigs 1kb cov5
GCA 010776735v1
GCA 010612715v1
GCA 010422515v1
GCA 008878465v1
GCA 006974585v1
GCA 008852985v1
GCA 007144545v1
GCA 008275305v1
GCA 008139715v1
GCA 017363245v1
SRR7039809 contigs 1kb cov5
GCA 019560635v1
GCA 006161325v1
GCA 007139465v1
GCA 007166025v1
GCA 015906405v1
GCA 015906345v1
GCA 007170115v1
GCA 011418255v1
GCA 010664525v1
GCA 010676685v1
GCA 009998065v1
GCA 005827425v1
GCA 003737305v1
GCA 003906035v1
GCA 005723575v1
GCA 005751955v1
GCA 008322345v1
GCA 011192835v1
GCA 010008925v1
GCA 007080845v1
GCA 006249755v1
GCA 006297925v1
GCA 017296435v1
SRR6345345 contigs 1kb cov5
SRR6133561 contigs 1kb cov5
GCA 007010965v1
GCA 007246115v1
GCA 010612695v1
GCA 019160875v1
GCA 011173155v1
GCA 007075505v1
GCA 007075725v1
SRR6760488 contigs 1kb cov5
SRR6051261 contigs 1kb cov5
GCA 021196515v1
GCA 014628255v1
SRR18656131 contigs 1kb cov5
SRR18605207 contigs 1kb cov5
SRR16149090 contigs 1kb cov5
SRR12677094 contigs 1kb cov5
GCA 010426605v1
GCA 016495305v1
GCA 016024715v1

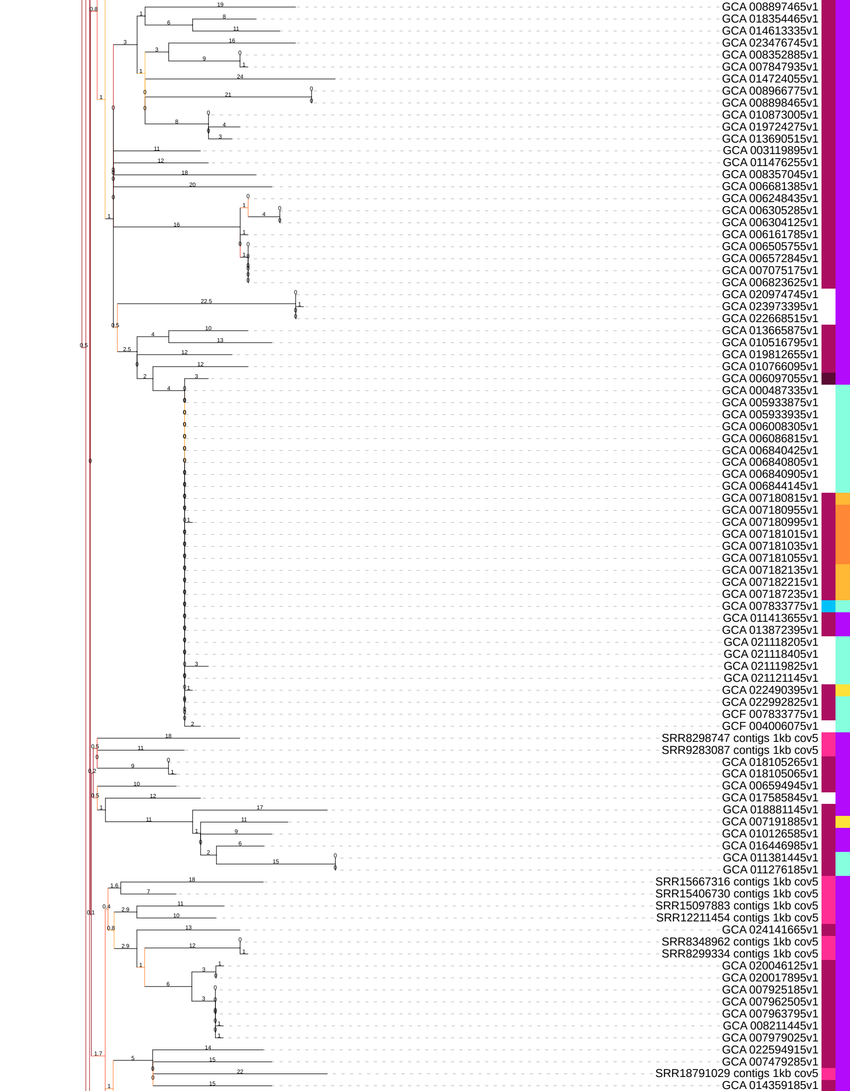
GCA 008897465v1
GCA 018354465v1
GCA 014613335v1
GCA 023476745v1
GCA 008352885v1
GCA 007847935v1
GCA 014724055v1
GCA 008966775v1
GCA 008898465v1
GCA 010873005v1
GCA 019724275v1
GCA 013690515v1
GCA 003119895v1
GCA 011476255v1
GCA 008357045v1
GCA 006681385v1
GCA 006248435v1
GCA 006305285v1
GCA 006304125v1
GCA 006161785v1
GCA 006505755v1
GCA 006572845v1
GCA 007075175v1
GCA 006823625v1
GCA 020974745v1
GCA 023973395v1
GCA 022668515v1
GCA 013665875v1
GCA 010516795v1
GCA 019812655v1
GCA 010766095v1
GCA 006097055v1
GCA 000487335v1
GCA 005933875v1
GCA 005933935v1
GCA 006008305v1
GCA 006086815v1
GCA 006840425v1
GCA 006840805v1
GCA 006840905v1
GCA 006844145v1
GCA 007180815v1
GCA 007180955v1
GCA 007180995v1
GCA 007181015v1
GCA 007181035v1
GCA 007181055v1
GCA 007182135v1
GCA 007182215v1
GCA 007187235v1
GCA 007833775v1
GCA 011413655v1
GCA 013872395v1
GCA 021118205v1
GCA 021118405v1
GCA 021119825v1
GCA 021121145v1
GCA 022490395v1
GCA 022992825v1
GCF 007833775v1
GCF 004006075v1
SRR8298747 contigs 1kb cov5
SRR9283087 contigs 1kb cov5
GCA 018105265v1
GCA 018105065v1
GCA 006594945v1
GCA 017585845v1
GCA 018881145v1
GCA 007191885v1
GCA 010126585v1
GCA 016446985v1
GCA 011381445v1
GCA 011276185v1
SRR15667316 contigs 1kb cov5
SRR15406730 contigs 1kb cov5
SRR15097883 contigs 1kb cov5
SRR12211454 contigs 1kb cov5
GCA 024141665v1
SRR8348962 contigs 1kb cov5
SRR8299334 contigs 1kb cov5
GCA 020046125v1
GCA 020017895v1
GCA 007925185v1
GCA 007962505v1
GCA 007963795v1
GCA 008211445v1
GCA 007979025v1
GCA 022594915v1
GCA 007479285v1
SRR18791029 contigs 1kb cov5
GCA 014359185v1

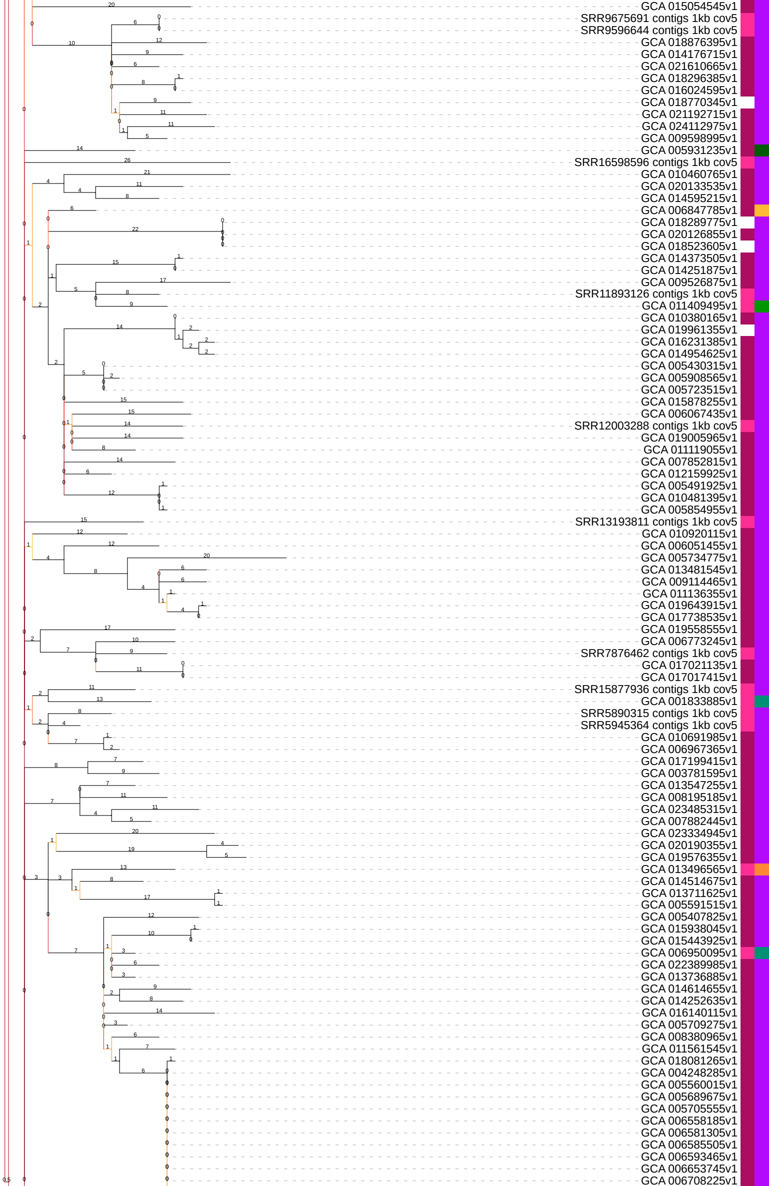
GCA 015054545v1
SRR9675691 contigs 1kb cov5
SRR9596644 contigs 1kb cov5
GCA 018876395v1
GCA 014176715v1
GCA 021610665v1
GCA 018296385v1
GCA 016024595v1
GCA 018770345v1
GCA 021192715v1
GCA 024112975v1
GCA 009598995v1
GCA 005931235v1
SRR16598596 contigs 1kb cov5
GCA 010460765v1
GCA 020133535v1
GCA 014595215v1
GCA 006847785v1
GCA 018289775v1
GCA 020126855v1
GCA 018523605v1
GCA 014373505v1
GCA 014251875v1
GCA 009526875v1
SRR11893126 contigs 1kb cov5
GCA 011409495v1
GCA 010380165v1
GCA 019961355v1
GCA 016231385v1
GCA 014954625v1
GCA 005430315v1
GCA 005908565v1
GCA 005723515v1
GCA 015878255v1
GCA 006067435v1
SRR12003288 contigs 1kb cov5
GCA 019005965v1
GCA 011119055v1
GCA 007852815v1
GCA 012159925v1
GCA 005491925v1
GCA 010481395v1
GCA 005854955v1
SRR13193811 contigs 1kb cov5
GCA 010920115v1
GCA 006051455v1
GCA 005734775v1
GCA 013481545v1
GCA 009114465v1
GCA 011136355v1
GCA 019643915v1
GCA 017738535v1
GCA 019558555v1
GCA 006773245v1
SRR7876462 contigs 1kb cov5
GCA 017021135v1
GCA 017017415v1
SRR15877936 contigs 1kb cov5
GCA 001833885v1
SRR5890315 contigs 1kb cov5
SRR5945364 contigs 1kb cov5
GCA 010691985v1
GCA 006967365v1
GCA 017199415v1
GCA 003781595v1
GCA 013547255v1
GCA 008195185v1
GCA 023485315v1
GCA 007882445v1
GCA 023334945v1
GCA 020190355v1
GCA 019576355v1
GCA 013496565v1
GCA 014514675v1
GCA 013711625v1
GCA 005591515v1
GCA 005407825v1
GCA 015938045v1
GCA 015443925v1
GCA 006950095v1
GCA 022389985v1
GCA 013736885v1
GCA 014614655v1
GCA 014252635v1
GCA 016140115v1
GCA 005709275v1
GCA 008380965v1
GCA 011561545v1
GCA 018081265v1
GCA 004248285v1
GCA 005560015v1
GCA 005689675v1
GCA 005705555v1
GCA 006558185v1
GCA 006581305v1
GCA 006585505v1
GCA 006593465v1
GCA 006653745v1
GCA 006708225v1
0.5

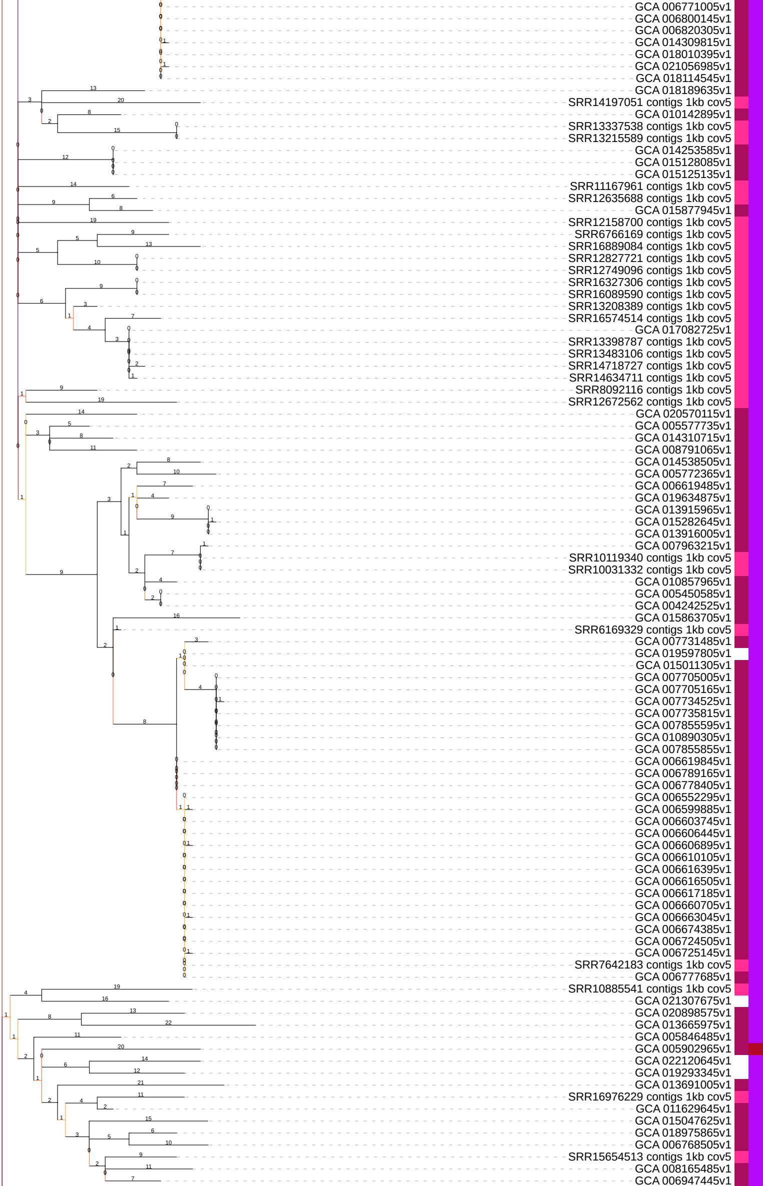
GCA 006771005v1
GCA 006800145v1
GCA 006820305v1
GCA 014309815v1
GCA 018010395v1
GCA 021056985v1
GCA 018114545v1
GCA 018189635v1
SRR14197051 contigs 1kb cov5
GCA 010142895v1
SRR13337538 contigs 1kb cov5
SRR13215589 contigs 1kb cov5
GCA 014253585v1
GCA 015128085v1
GCA 015125135v1
SRR11167961 contigs 1kb cov5
SRR12635688 contigs 1kb cov5
GCA 015877945v1
SRR12158700 contigs 1kb cov5
SRR6766169 contigs 1kb cov5
SRR16889084 contigs 1kb cov5
SRR12827721 contigs 1kb cov5
SRR12749096 contigs 1kb cov5
SRR16327306 contigs 1kb cov5
SRR16089590 contigs 1kb cov5
SRR13208389 contigs 1kb cov5
SRR16574514 contigs 1kb cov5
GCA 017082725v1
SRR13398787 contigs 1kb cov5
SRR13483106 contigs 1kb cov5
SRR14718727 contigs 1kb cov5
SRR14634711 contigs 1kb cov5
SRR8092116 contigs 1kb cov5
SRR12672562 contigs 1kb cov5
GCA 020570115v1
GCA 005577735v1
GCA 014310715v1
GCA 008791065v1
GCA 014538505v1
GCA 005772365v1
GCA 006619485v1
GCA 019634875v1
GCA 013915965v1
GCA 015282645v1
GCA 013916005v1
GCA 007963215v1
SRR10119340 contigs 1kb cov5
SRR10031332 contigs 1kb cov5
GCA 010857965v1
GCA 005450585v1
GCA 004242525v1
GCA 015863705v1
SRR6169329 contigs 1kb cov5
GCA 007731485v1
GCA 019597805v1
GCA 015011305v1
GCA 007705005v1
GCA 007705165v1
GCA 007734525v1
GCA 007735815v1
GCA 007855595v1
GCA 010890305v1
GCA 007855855v1
GCA 006619845v1
GCA 006789165v1
GCA 006778405v1
GCA 006552295v1
GCA 006599885v1
GCA 006603745v1
GCA 006606445v1
GCA 006606895v1
GCA 006610105v1
GCA 006616395v1
GCA 006616505v1
GCA 006617185v1
GCA 006660705v1
GCA 006663045v1
GCA 006674385v1
GCA 006724505v1
GCA 006725145v1
SRR7642183 contigs 1kb cov5
GCA 006777685v1
SRR10885541 contigs 1kb cov5
GCA 021307675v1
GCA 020898575v1
GCA 013665975v1
GCA 005846485v1
GCA 005902965v1
GCA 022120645v1
GCA 019293345v1
GCA 013691005v1
SRR16976229 contigs 1kb cov5
GCA 011629645v1
GCA 015047625v1
GCA 018975865v1
GCA 006768505v1
SRR15654513 contigs 1kb cov5
GCA 008165485v1
GCA 006947445v1

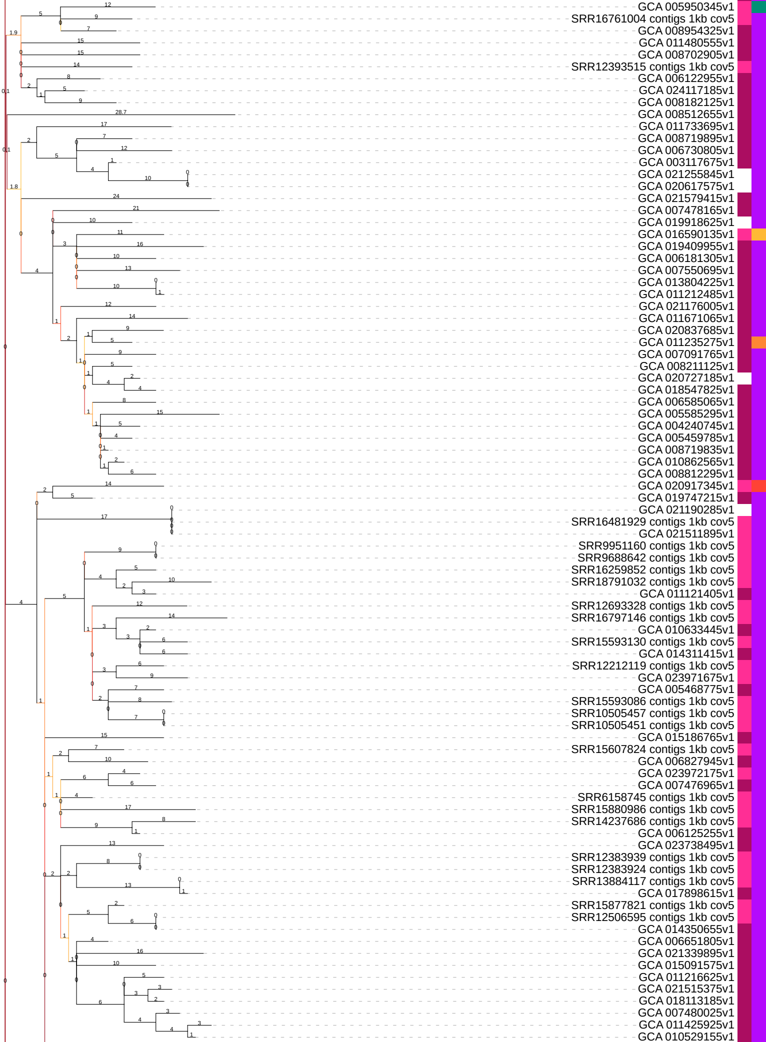
GCA 005950345v1
SRR16761004 contigs 1kb cov5
GCA 008954325v1
GCA 011480555v1
GCA 008702905v1
SRR12393515 contigs 1kb cov5
GCA 006122955v1
GCA 024117185v1
GCA 008182125v1
GCA 008512655v1
GCA 011733695v1
GCA 008719895v1
GCA 006730805v1
GCA 003117675v1
GCA 021255845v1
GCA 020617575v1
GCA 021579415v1
GCA 007478165v1
GCA 019918625v1
GCA 016590135v1
GCA 019409955v1
GCA 006181305v1
GCA 007550695v1
GCA 013804225v1
GCA 011212485v1
GCA 021176005v1
GCA 011671065v1
GCA 020837685v1
GCA 011235275v1
GCA 007091765v1
GCA 008211125v1
GCA 020727185v1
GCA 018547825v1
GCA 006585065v1
GCA 005585295v1
GCA 004240745v1
GCA 005459785v1
GCA 008719835v1
GCA 010862565v1
GCA 008812295v1
GCA 020917345v1
GCA 019747215v1
GCA 021190285v1
SRR16481929 contigs 1kb cov5
GCA 021511895v1
SRR9951160 contigs 1kb cov5
SRR9688642 contigs 1kb cov5
SRR16259852 contigs 1kb cov5
SRR18791032 contigs 1kb cov5
GCA 011121405v1
SRR12693328 contigs 1kb cov5
SRR16797146 contigs 1kb cov5
GCA 010633445v1
SRR15593130 contigs 1kb cov5
GCA 014311415v1
SRR12212119 contigs 1kb cov5
GCA 023971675v1
GCA 005468775v1
SRR15593086 contigs 1kb cov5
SRR10505457 contigs 1kb cov5
SRR10505451 contigs 1kb cov5
GCA 015186765v1
SRR15607824 contigs 1kb cov5
GCA 006827945v1
GCA 023972175v1
GCA 007476965v1
SRR6158745 contigs 1kb cov5
SRR15880986 contigs 1kb cov5
SRR14237686 contigs 1kb cov5
GCA 006125255v1
GCA 023738495v1
SRR12383939 contigs 1kb cov5
SRR12383924 contigs 1kb cov5
SRR13884117 contigs 1kb cov5
GCA 017898615v1
SRR15877821 contigs 1kb cov5
SRR12506595 contigs 1kb cov5
GCA 014350655v1
GCA 006651805v1
GCA 021339895v1
GCA 015091575v1
GCA 011216625v1
GCA 021515375v1
GCA 018113185v1
GCA 007480025v1
GCA 011425925v1
GCA 010529155v1

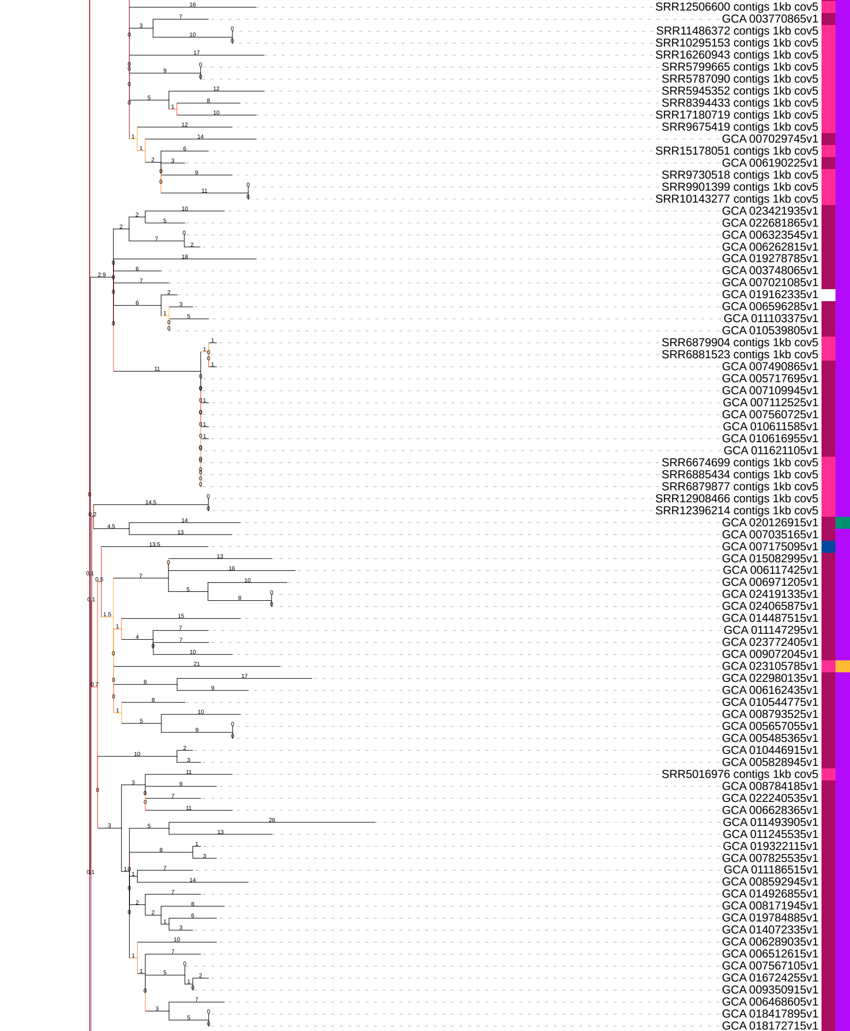
SRR12506600 contigs 1kb cov5
GCA 003770865v1
SRR11486372 contigs 1kb cov5
SRR10295153 contigs 1kb cov5
SRR16260943 contigs 1kb cov5
SRR5799665 contigs 1kb cov5
SRR5787090 contigs 1kb cov5
SRR5945352 contigs 1kb cov5
SRR8394433 contigs 1kb cov5
SRR17180719 contigs 1kb cov5
SRR9675419 contigs 1kb cov5
GCA 007029745v1
SRR15178051 contigs 1kb cov5
GCA 006190225v1
SRR9730518 contigs 1kb cov5
SRR9901399 contigs 1kb cov5
SRR10143277 contigs 1kb cov5
GCA 023421935v1
GCA 022681865v1
GCA 006323545v1
GCA 006262815v1
GCA 019278785v1
GCA 003748065v1
GCA 007021085v1
GCA 019162335v1
GCA 006596285v1
GCA 011103375v1
GCA 010539805v1
SRR6879904 contigs 1kb cov5
SRR6881523 contigs 1kb cov5
GCA 007490865v1
GCA 005717695v1
GCA 007109945v1
GCA 007112525v1
GCA 007560725v1
GCA 010611585v1
GCA 010616955v1
GCA 011621105v1
SRR6674699 contigs 1kb cov5
SRR6885434 contigs 1kb cov5
SRR6879877 contigs 1kb cov5
SRR12908466 contigs 1kb cov5
SRR12396214 contigs 1kb cov5
GCA 020126915v1
GCA 007035165v1
GCA 007175095v1
GCA 015082995v1
GCA 006117425v1
GCA 006971205v1
GCA 024191335v1
GCA 024065875v1
GCA 014487515v1
GCA 011147295v1
GCA 023772405v1
GCA 009072045v1
GCA 023105785v1
GCA 022980135v1
GCA 006162435v1
GCA 010544775v1
GCA 008793525v1
GCA 005657055v1
GCA 005485365v1
GCA 010446915v1
GCA 005828945v1
SRR5016976 contigs 1kb cov5
GCA 008784185v1
GCA 022240535v1
GCA 006628365v1
GCA 011493905v1
GCA 011245535v1
GCA 019322115v1
GCA 007825535v1
GCA 011186515v1
GCA 008592945v1
GCA 014926855v1
GCA 008171945v1
GCA 019784885v1
GCA 014072335v1
GCA 006289035v1
GCA 006512615v1
GCA 007567105v1
GCA 016724255v1
GCA 009350915v1
GCA 006468605v1
GCA 018417895v1
GCA 018172715v1

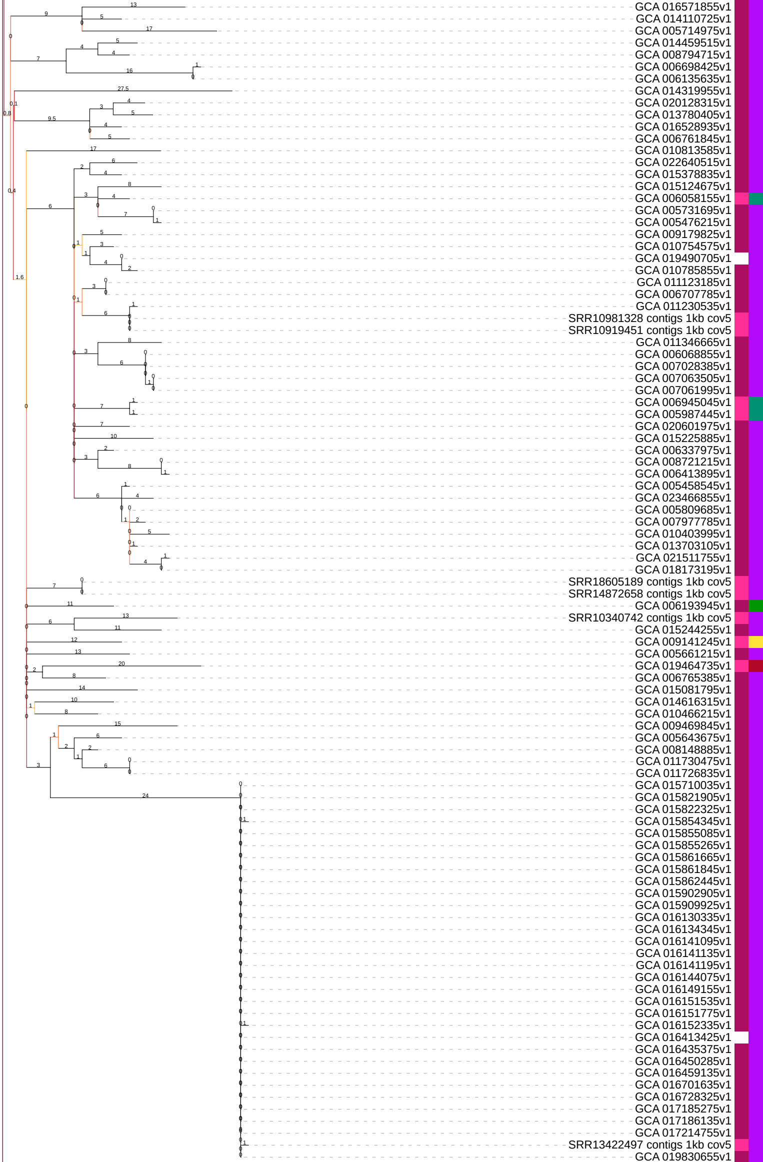
GCA 016571855v1
GCA 014110725v1
GCA 005714975v1
GCA 014459515v1
GCA 008794715v1
GCA 006698425v1
GCA 006135635v1
GCA 014319955v1
GCA 020128315v1
GCA 013780405v1
GCA 016528935v1
GCA 006761845v1
GCA 010813585v1
GCA 022640515v1
GCA 015378835v1
GCA 015124675v1
GCA 006058155v1
GCA 005731695v1
GCA 005476215v1
GCA 009179825v1
GCA 010754575v1
GCA 019490705v1
GCA 010785855v1
GCA 011123185v1
GCA 006707785v1
GCA 011230535v1
SRR10981328 contigs 1kb cov5
SRR10919451 contigs 1kb cov5
GCA 011346665v1
GCA 006068855v1
GCA 007028385v1
GCA 007063505v1
GCA 007061995v1
GCA 006945045v1
GCA 005987445v1
GCA 020601975v1
GCA 015225885v1
GCA 006337975v1
GCA 008721215v1
GCA 006413895v1
GCA 005458545v1
GCA 023466855v1
GCA 005809685v1
GCA 007977785v1
GCA 010403995v1
GCA 013703105v1
GCA 021511755v1
GCA 018173195v1
SRR18605189 contigs 1kb cov5
SRR14872658 contigs 1kb cov5
GCA 006193945v1
SRR10340742 contigs 1kb cov5
GCA 015244255v1
GCA 009141245v1
GCA 005661215v1
GCA 019464735v1
GCA 006765385v1
GCA 015081795v1
GCA 014616315v1
GCA 010466215v1
GCA 009469845v1
GCA 005643675v1
GCA 008148885v1
GCA 011730475v1
GCA 011726835v1
GCA 015710035v1
GCA 015821905v1
GCA 015822325v1
GCA 015854345v1
GCA 015855085v1
GCA 015855265v1
GCA 015861665v1
GCA 015861845v1
GCA 015862445v1
GCA 015902905v1
GCA 015909925v1
GCA 016130335v1
GCA 016134345v1
GCA 016141095v1
GCA 016141135v1
GCA 016141195v1
GCA 016144075v1
GCA 016149155v1
GCA 016151535v1
GCA 016151775v1
GCA 016152335v1
GCA 016413425v1
GCA 016435375v1
GCA 016450285v1
GCA 016459135v1
GCA 016701635v1
GCA 016728325v1
GCA 017185275v1
GCA 017186135v1
GCA 017214755v1
SRR13422497 contigs 1kb cov5
GCA 019830655v1

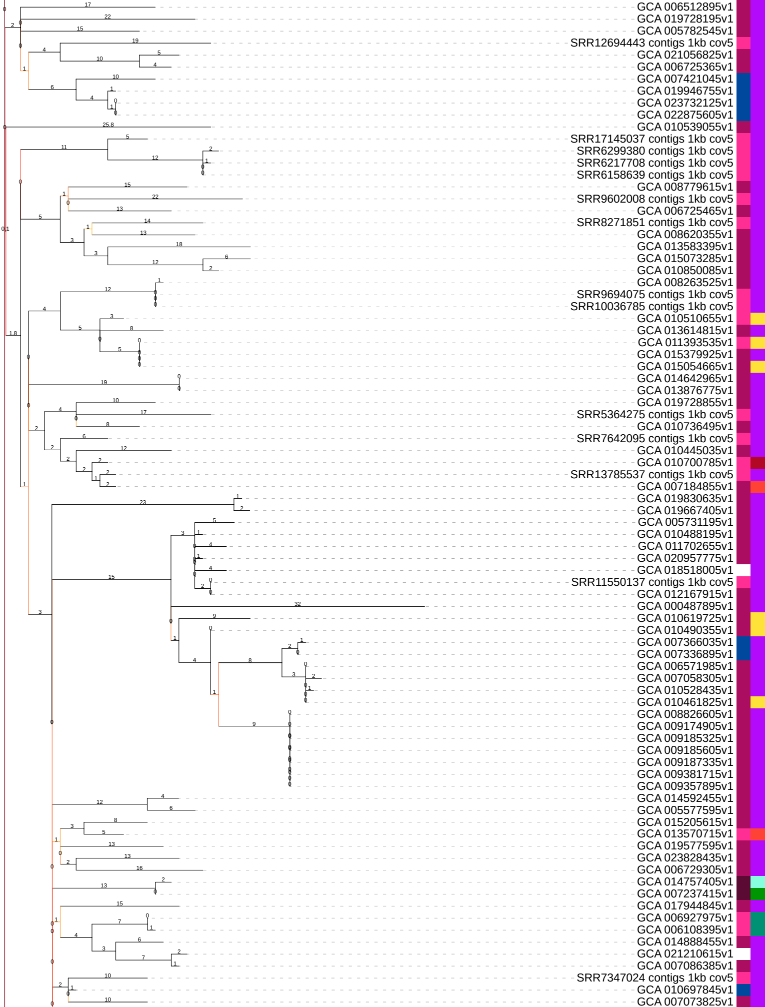
GCA 006512895v1
GCA 019728195v1
GCA 005782545v1
SRR12694443 contigs 1kb cov5
GCA 021056825v1
GCA 006725365v1
GCA 007421045v1
GCA 019946755v1
GCA 023732125v1
GCA 022875605v1
GCA 010539055v1
SRR17145037 contigs 1kb cov5
SRR6299380 contigs 1kb cov5
SRR6217708 contigs 1kb cov5
SRR6158639 contigs 1kb cov5
GCA 008779615v1
SRR9602008 contigs 1kb cov5
GCA 006725465v1
SRR8271851 contigs 1kb cov5
GCA 008620355v1
GCA 013583395v1
GCA 015073285v1
GCA 010850085v1
GCA 008263525v1
SRR9694075 contigs 1kb cov5
SRR10036785 contigs 1kb cov5
GCA 010510655v1
GCA 013614815v1
GCA 011393535v1
GCA 015379925v1
GCA 015054665v1
GCA 014642965v1
GCA 013876775v1
GCA 019728855v1
SRR5364275 contigs 1kb cov5
GCA 010736495v1
SRR7642095 contigs 1kb cov5
GCA 010445035v1
GCA 010700785v1
SRR13785537 contigs 1kb cov5
GCA 007184855v1
GCA 019830635v1
GCA 019667405v1
GCA 005731195v1
GCA 010488195v1
GCA 011702655v1
GCA 020957775v1
GCA 018518005v1
SRR11550137 contigs 1kb cov5
GCA 012167915v1
GCA 000487895v1
GCA 010619725v1
GCA 010490355v1
GCA 007366035v1
GCA 007336895v1
GCA 006571985v1
GCA 007058305v1
GCA 010528435v1
GCA 010461825v1
GCA 008826605v1
GCA 009174905v1
GCA 009185325v1
GCA 009185605v1
GCA 009187335v1
GCA 009381715v1
GCA 009357895v1
GCA 014592455v1
GCA 005577595v1
GCA 015205615v1
GCA 013570715v1
GCA 019577595v1
GCA 023828435v1
GCA 006729305v1
GCA 014757405v1
GCA 007237415v1
GCA 017944845v1
GCA 006927975v1
GCA 006108395v1
GCA 014888455v1
GCA 021210615v1
GCA 007086385v1
SRR7347024 contigs 1kb cov5
GCA 010697845v1
GCA 007073825v1

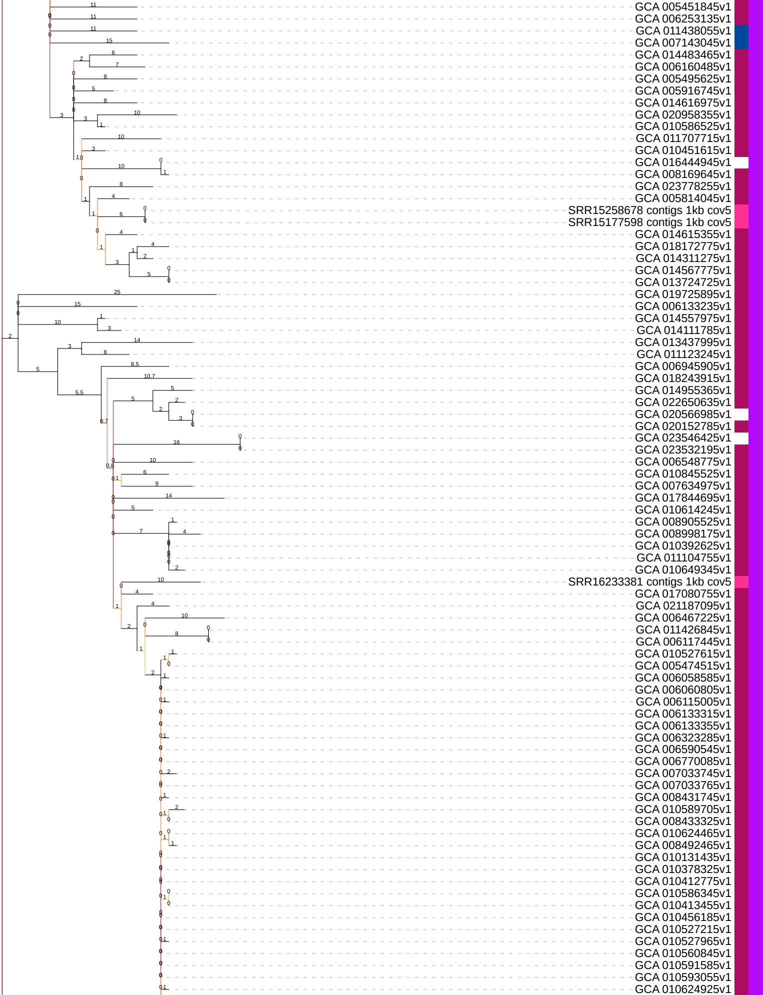
GCA 005451845v1
GCA 006253135v1
GCA 011438055v1
GCA 007143045v1
GCA 014483465v1
GCA 006160485v1
GCA 005495625v1
GCA 005916745v1
GCA 014616975v1
GCA 020958355v1
GCA 010586525v1
GCA 011707715v1
GCA 010451615v1
GCA 016444945v1
GCA 008169645v1
GCA 023778255v1
GCA 005814045v1
SRR15258678 contigs 1kb cov5
SRR15177598 contigs 1kb cov5
GCA 014615355v1
GCA 018172775v1
GCA 014311275v1
GCA 014567775v1
GCA 013724725v1
GCA 019725895v1
GCA 006133235v1
GCA 014557975v1
GCA 014111785v1
GCA 013437995v1
GCA 011123245v1
GCA 006945905v1
GCA 018243915v1
GCA 014955365v1
GCA 022650635v1
GCA 020566985v1
GCA 020152785v1
GCA 023546425v1
GCA 023532195v1
GCA 006548775v1
GCA 010845525v1
GCA 007634975v1
GCA 017844695v1
GCA 010614245v1
GCA 008905525v1
GCA 008998175v1
GCA 010392625v1
GCA 011104755v1
GCA 010649345v1
SRR16233381 contigs 1kb cov5
GCA 017080755v1
GCA 021187095v1
GCA 006467225v1
GCA 011426845v1
GCA 006117445v1
GCA 010527615v1
GCA 005474515v1
GCA 006058585v1
GCA 006060805v1
GCA 006115005v1
GCA 006133315v1
GCA 006133355v1
GCA 006323285v1
GCA 006590545v1
GCA 006770085v1
GCA 007033745v1
GCA 007033765v1
GCA 008431745v1
GCA 010589705v1
GCA 008433325v1
GCA 010624465v1
GCA 008492465v1
GCA 010131435v1
GCA 010378325v1
GCA 010412775v1
GCA 010586345v1
GCA 010413455v1
GCA 010456185v1
GCA 010527215v1
GCA 010527965v1
GCA 010560845v1
GCA 010591585v1
GCA 010593055v1
GCA 010624925v1

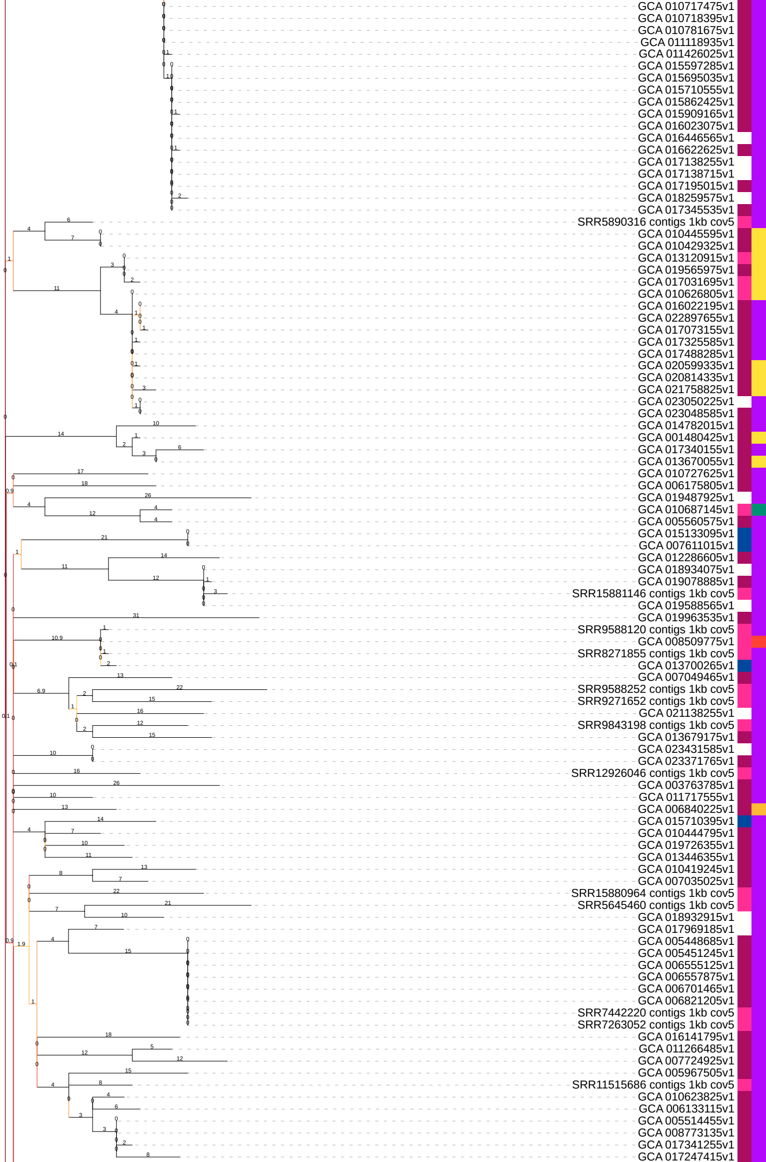
GCA 010717475v1
GCA 010718395v1
GCA 010781675v1
GCA 011118935v1
GCA 011426025v1
GCA 015597285v1
GCA 015695035v1
GCA 015710555v1
GCA 015862425v1
GCA 015909165v1
GCA 016023075v1
GCA 016446565v1
GCA 016622625v1
GCA 017138255v1
GCA 017138715v1
GCA 017195015v1
GCA 018259575v1
GCA 017345535v1
SRR5890316 contigs 1kb cov5
GCA 010445595v1
GCA 010429325v1
GCA 013120915v1
GCA 019565975v1
GCA 017031695v1
GCA 010626805v1
GCA 016022195v1
GCA 022897655v1
GCA 017073155v1
GCA 017325585v1
GCA 017488285v1
GCA 020599335v1
GCA 020814335v1
GCA 021758825v1
GCA 023050225v1
GCA 023048585v1
GCA 014782015v1
GCA 001480425v1
GCA 017340155v1
GCA 013670055v1
GCA 010727625v1
GCA 006175805v1
GCA 019487925v1
GCA 010687145v1
GCA 005560575v1
GCA 015133095v1
GCA 007611015v1
GCA 012286605v1
GCA 018934075v1
GCA 019078885v1
SRR15881146 contigs 1kb cov5
GCA 019588565v1
GCA 019963535v1
SRR9588120 contigs 1kb cov5
GCA 008509775v1
SRR8271855 contigs 1kb cov5
GCA 013700265v1
GCA 007049465v1
SRR9588252 contigs 1kb cov5
SRR9271652 contigs 1kb cov5
GCA 021138255v1
SRR9843198 contigs 1kb cov5
GCA 013679175v1
GCA 023431585v1
GCA 023371765v1
SRR12926046 contigs 1kb cov5
GCA 003763785v1
GCA 011717555v1
GCA 006840225v1
GCA 015710395v1
GCA 010444795v1
GCA 019726355v1
GCA 013446355v1
GCA 010419245v1
GCA 007035025v1
SRR15880964 contigs 1kb cov5
SRR5645460 contigs 1kb cov5
GCA 018932915v1
GCA 017969185v1
GCA 005448685v1
GCA 005451245v1
GCA 006555125v1
GCA 006557875v1
GCA 006701465v1
GCA 006821205v1
SRR7442220 contigs 1kb cov5
SRR7263052 contigs 1kb cov5
GCA 016141795v1
GCA 011266485v1
GCA 007724925v1
GCA 005967505v1
SRR11515686 contigs 1kb cov5
GCA 010623825v1
GCA 006133115v1
GCA 005514455v1
GCA 008773135v1
GCA 017341255v1
GCA 017247415v1

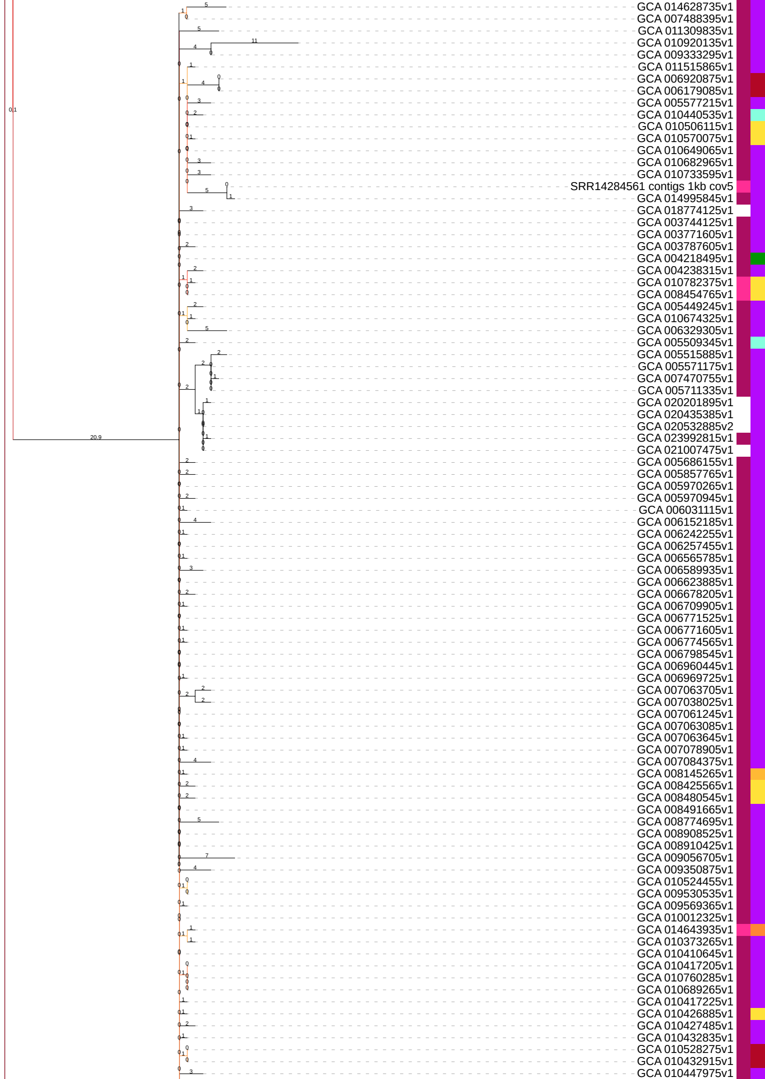
0.1
20.9
GCA 014628735v1
GCA 007488395v1
GCA 011309835v1
GCA 010920135v1
GCA 009333295v1
GCA 011515865v1
GCA 006920875v1
GCA 006179085v1
GCA 005577215v1
GCA 010440535v1
GCA 010506115v1
GCA 010570075v1
GCA 010649065v1
GCA 010682965v1
GCA 010733595v1
SRR14284561 contigs 1kb cov5
GCA 014995845v1
GCA 018774125v1
GCA 003744125v1
GCA 003771605v1
GCA 003787605v1
GCA 004218495v1
GCA 004238315v1
GCA 010782375v1
GCA 008454765v1
GCA 005449245v1
GCA 010674325v1
GCA 006329305v1
GCA 005509345v1
GCA 005515885v1
GCA 005571175v1
GCA 007470755v1
GCA 005711335v1
GCA 020201895v1
GCA 020435385v1
GCA 020532885v2
GCA 023992815v1
GCA 021007475v1
GCA 005686155v1
GCA 005857765v1
GCA 005970265v1
GCA 005970945v1
GCA 006031115v1
GCA 006152185v1
GCA 006242255v1
GCA 006257455v1
GCA 006565785v1
GCA 006589935v1
GCA 006623885v1
GCA 006678205v1
GCA 006709905v1
GCA 006771525v1
GCA 006771605v1
GCA 006774565v1
GCA 006798545v1
GCA 006960445v1
GCA 006969725v1
GCA 007063705v1
GCA 007038025v1
GCA 007061245v1
GCA 007063085v1
GCA 007063645v1
GCA 007078905v1
GCA 007084375v1
GCA 008145265v1
GCA 008425565v1
GCA 008480545v1
GCA 008491665v1
GCA 008774695v1
GCA 008908525v1
GCA 008910425v1
GCA 009056705v1
GCA 009350875v1
GCA 010524455v1
GCA 009530535v1
GCA 009569365v1
GCA 010012325v1
GCA 014643935v1
GCA 010373265v1
GCA 010410645v1
GCA 010417205v1
GCA 010760285v1
GCA 010689265v1
GCA 010417225v1
GCA 010426885v1
GCA 010427485v1
GCA 010432835v1
GCA 010528275v1
GCA 010432915v1
GCA 010447975v1

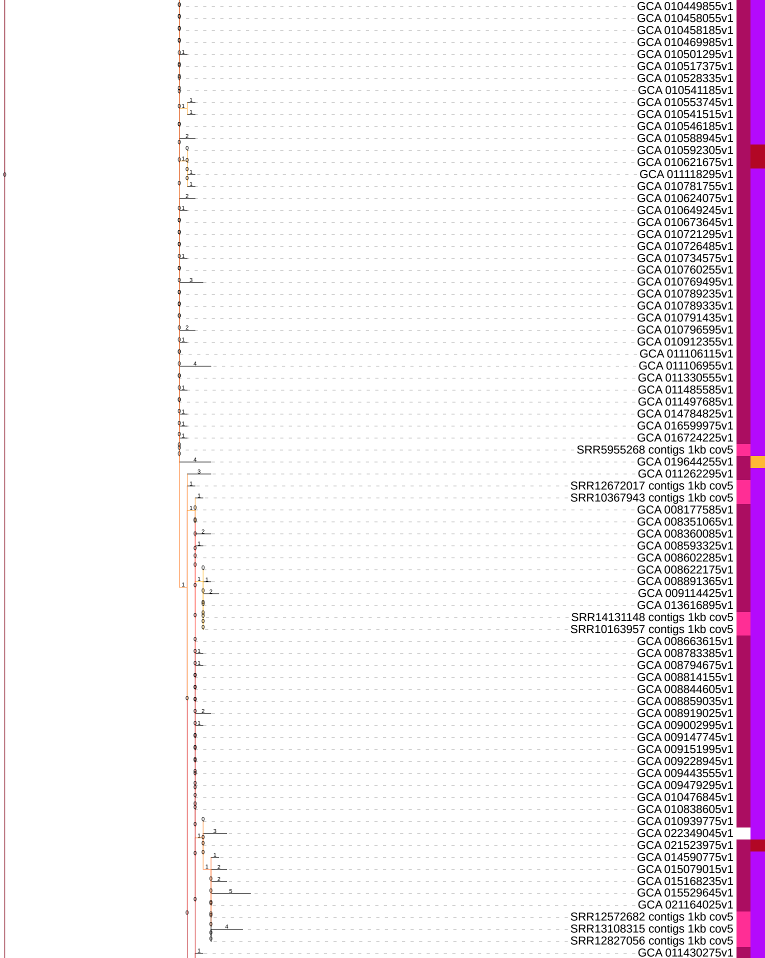
GCA 010449855v1
GCA 010458055v1
GCA 010458185v1
GCA 010469985v1
GCA 010501295v1
GCA 010517375v1
GCA 010528335v1
GCA 010541185v1
GCA 010553745v1
GCA 010541515v1
GCA 010546185v1
GCA 010588945v1
GCA 010592305v1
GCA 010621675v1
GCA 011118295v1
GCA 010781755v1
GCA 010624075v1
GCA 010649245v1
GCA 010673645v1
GCA 010721295v1
GCA 010726485v1
GCA 010734575v1
GCA 010760255v1
GCA 010769495v1
GCA 010789235v1
GCA 010789335v1
GCA 010791435v1
GCA 010796595v1
GCA 010912355v1
GCA 011106115v1
GCA 011106955v1
GCA 011330555v1
GCA 011485585v1
GCA 011497685v1
GCA 014784825v1
GCA 016599975v1
GCA 016724225v1
SRR5955268 contigs 1kb cov5
GCA 019644255v1
GCA 011262295v1
SRR12672017 contigs 1kb cov5
SRR10367943 contigs 1kb cov5
GCA 008177585v1
GCA 008351065v1
GCA 008360085v1
GCA 008593325v1
GCA 008602285v1
GCA 008622175v1
GCA 008891365v1
GCA 009114425v1
GCA 013616895v1
SRR14131148 contigs 1kb cov5
SRR10163957 contigs 1kb cov5
GCA 008663615v1
GCA 008783385v1
GCA 008794675v1
GCA 008814155v1
GCA 008844605v1
GCA 008859035v1
GCA 008919025v1
GCA 009002995v1
GCA 009147745v1
GCA 009151995v1
GCA 009228945v1
GCA 009443555v1
GCA 009479295v1
GCA 010476845v1
GCA 010838605v1
GCA 010939775v1
GCA 022349045v1
GCA 021523975v1
GCA 014590775v1
GCA 015079015v1
GCA 015168235v1
GCA 015529645v1
GCA 021164025v1
SRR12572682 contigs 1kb cov5
SRR13108315 contigs 1kb cov5
SRR12827056 contigs 1kb cov5
GCA 011430275v1

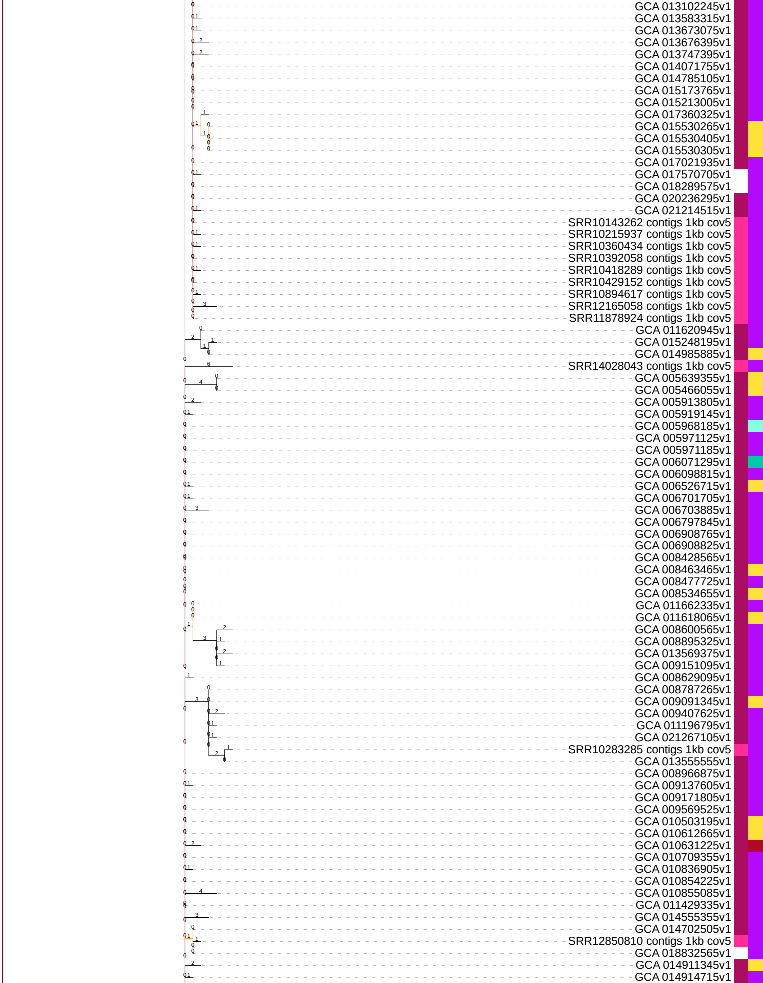
GCA 013102245v1
GCA 013583315v1
GCA 013673075v1
GCA 013676395v1
GCA 013747395v1
GCA 014071755v1
GCA 014785105v1
GCA 015173765v1
GCA 015213005v1
GCA 017360325v1
GCA 015530265v1
GCA 015530405v1
GCA 015530305v1
GCA 017021935v1
GCA 017570705v1
GCA 018289575v1
GCA 020236295v1
GCA 021214515v1
SRR10143262 contigs 1kb cov5
SRR10215937 contigs 1kb cov5
SRR10360434 contigs 1kb cov5
SRR10392058 contigs 1kb cov5
SRR10418289 contigs 1kb cov5
SRR10429152 contigs 1kb cov5
SRR10894617 contigs 1kb cov5
SRR12165058 contigs 1kb cov5
SRR11878924 contigs 1kb cov5
GCA 011620945v1
GCA 015248195v1
GCA 014985885v1
SRR14028043 contigs 1kb cov5
GCA 005639355v1
GCA 005466055v1
GCA 005913805v1
GCA 005919145v1
GCA 005968185v1
GCA 005971125v1
GCA 005971185v1
GCA 006071295v1
GCA 006098815v1
GCA 006526715v1
GCA 006701705v1
GCA 006703885v1
GCA 006797845v1
GCA 006908765v1
GCA 006908825v1
GCA 008428565v1
GCA 008463465v1
GCA 008477725v1
GCA 008534655v1
GCA 011662335v1
GCA 011618065v1
GCA 008600565v1
GCA 008895325v1
GCA 013569375v1
GCA 009151095v1
GCA 008629095v1
GCA 008787265v1
GCA 009091345v1
GCA 009407625v1
GCA 011196795v1
GCA 021267105v1
SRR10283285 contigs 1kb cov5
GCA 013555555v1
GCA 008966875v1
GCA 009137605v1
GCA 009171805v1
GCA 009569525v1
GCA 010503195v1
GCA 010612665v1
GCA 010631225v1
GCA 010709355v1
GCA 010836905v1
GCA 010854225v1
GCA 010855085v1
GCA 011429335v1
GCA 014555355v1
GCA 014702505v1
SRR12850810 contigs 1kb cov5
GCA 018832565v1
GCA 014911345v1
GCA 014914715v1

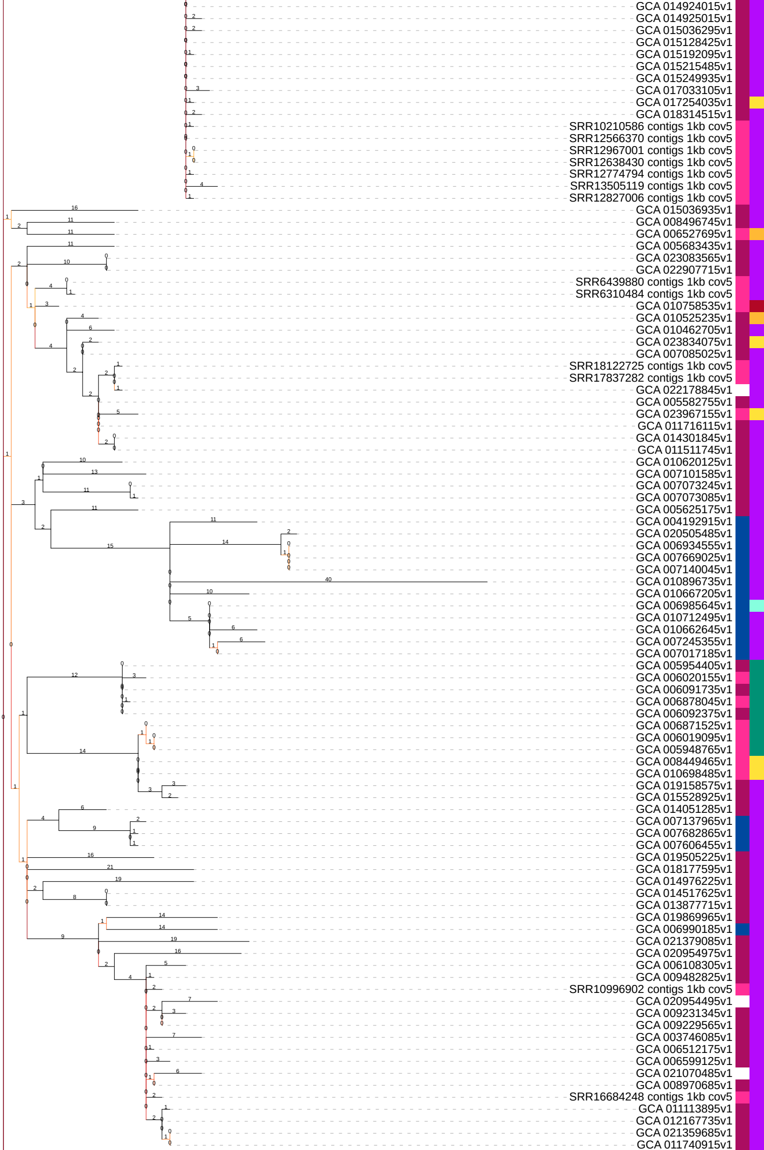
GCA 014924015v1
GCA 014925015v1
GCA 015036295v1
GCA 015128425v1
GCA 015192095v1
GCA 015215485v1
GCA 015249935v1
GCA 017033105v1
GCA 017254035v1
GCA 018314515v1
SRR10210586 contigs 1kb cov5
SRR12566370 contigs 1kb cov5
SRR12967001 contigs 1kb cov5
SRR12638430 contigs 1kb cov5
SRR12774794 contigs 1kb cov5
SRR13505119 contigs 1kb cov5
SRR12827006 contigs 1kb cov5
GCA 015036935v1
GCA 008496745v1
GCA 006527695v1
GCA 005683435v1
GCA 023083565v1
GCA 022907715v1
SRR6439880 contigs 1kb cov5
SRR6310484 contigs 1kb cov5
GCA 010758535v1
GCA 010525235v1
GCA 010462705v1
GCA 023834075v1
GCA 007085025v1
SRR18122725 contigs 1kb cov5
SRR17837282 contigs 1kb cov5
GCA 022178845v1
GCA 005582755v1
GCA 023967155v1
GCA 011716115v1
GCA 014301845v1
GCA 011511745v1
GCA 010620125v1
GCA 007101585v1
GCA 007073245v1
GCA 007073085v1
GCA 005625175v1
GCA 004192915v1
GCA 020505485v1
GCA 006934555v1
GCA 007669025v1
GCA 007140045v1
GCA 010896735v1
GCA 010667205v1
GCA 006985645v1
GCA 010712495v1
GCA 010662645v1
GCA 007245355v1
GCA 007017185v1
GCA 005954405v1
GCA 006020155v1
GCA 006091735v1
GCA 006878045v1
GCA 006092375v1
GCA 006871525v1
GCA 006019095v1
GCA 005948765v1
GCA 008449465v1
GCA 010698485v1
GCA 019158575v1
GCA 015528925v1
GCA 014051285v1
GCA 007137965v1
GCA 007682865v1
GCA 007606455v1
GCA 019505225v1
GCA 018177595v1
GCA 014976225v1
GCA 014517625v1
GCA 013877715v1
GCA 019869965v1
GCA 006990185v1
GCA 021379085v1
GCA 020954975v1
GCA 006108305v1
GCA 009482825v1
SRR10996902 contigs 1kb cov5
GCA 020954495v1
GCA 009231345v1
GCA 009229565v1
GCA 003746085v1
GCA 006512175v1
GCA 006599125v1
GCA 021070485v1
GCA 008970685v1
SRR16684248 contigs 1kb cov5
GCA 011113895v1
GCA 012167735v1
GCA 021359685v1
GCA 011740915v1

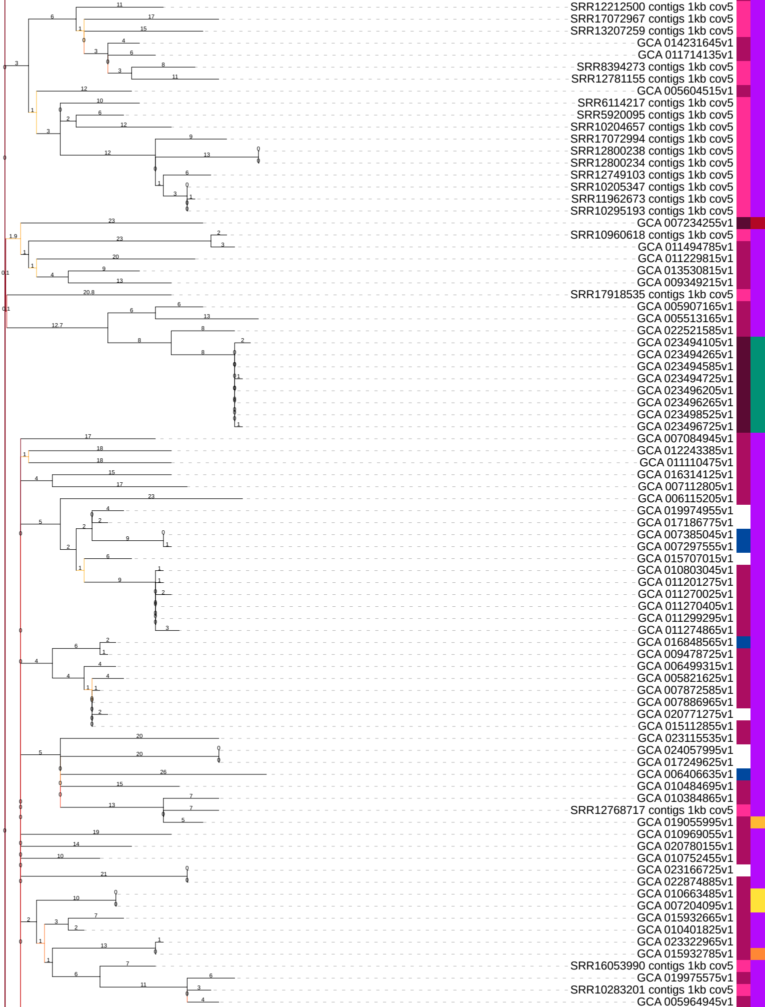
SRR12212500 contigs 1kb cov5
SRR17072967 contigs 1kb cov5
SRR13207259 contigs 1kb cov5
GCA 014231645v1
GCA 011714135v1
SRR8394273 contigs 1kb cov5
SRR12781155 contigs 1kb cov5
GCA 005604515v1
SRR6114217 contigs 1kb cov5
SRR5920095 contigs 1kb cov5
SRR10204657 contigs 1kb cov5
SRR17072994 contigs 1kb cov5
SRR12800238 contigs 1kb cov5
SRR12800234 contigs 1kb cov5
SRR12749103 contigs 1kb cov5
SRR10205347 contigs 1kb cov5
SRR11962673 contigs 1kb cov5
SRR10295193 contigs 1kb cov5
GCA 007234255v1
SRR10960618 contigs 1kb cov5
GCA 011494785v1
GCA 011229815v1
GCA 013530815v1
GCA 009349215v1
SRR17918535 contigs 1kb cov5
GCA 005907165v1
GCA 005513165v1
GCA 022521585v1
GCA 023494105v1
GCA 023494265v1
GCA 023494585v1
GCA 023494725v1
GCA 023496205v1
GCA 023496265v1
GCA 023498525v1
GCA 023496725v1
GCA 007084945v1
GCA 012243385v1
GCA 011110475v1
GCA 016314125v1
GCA 007112805v1
GCA 006115205v1
GCA 019974955v1
GCA 017186775v1
GCA 007385045v1
GCA 007297555v1
GCA 015707015v1
GCA 010803045v1
GCA 011201275v1
GCA 011270025v1
GCA 011270405v1
GCA 011299295v1
GCA 011274865v1
GCA 016848565v1
GCA 009478725v1
GCA 006499315v1
GCA 005821625v1
GCA 007872585v1
GCA 007886965v1
GCA 020771275v1
GCA 015112855v1
GCA 023115535v1
GCA 024057995v1
GCA 017249625v1
GCA 006406635v1
GCA 010484695v1
GCA 010384865v1
SRR12768717 contigs 1kb cov5
GCA 019055995v1
GCA 010969055v1
GCA 020780155v1
GCA 010752455v1
GCA 023166725v1
GCA 022874885v1
GCA 010663485v1
GCA 007204095v1
GCA 015932665v1
GCA 010401825v1
GCA 023322965v1
GCA 015932785v1
SRR16053990 contigs 1kb cov5
GCA 019975575v1
SRR10283201 contigs 1kb cov5
GCA 005964945v1

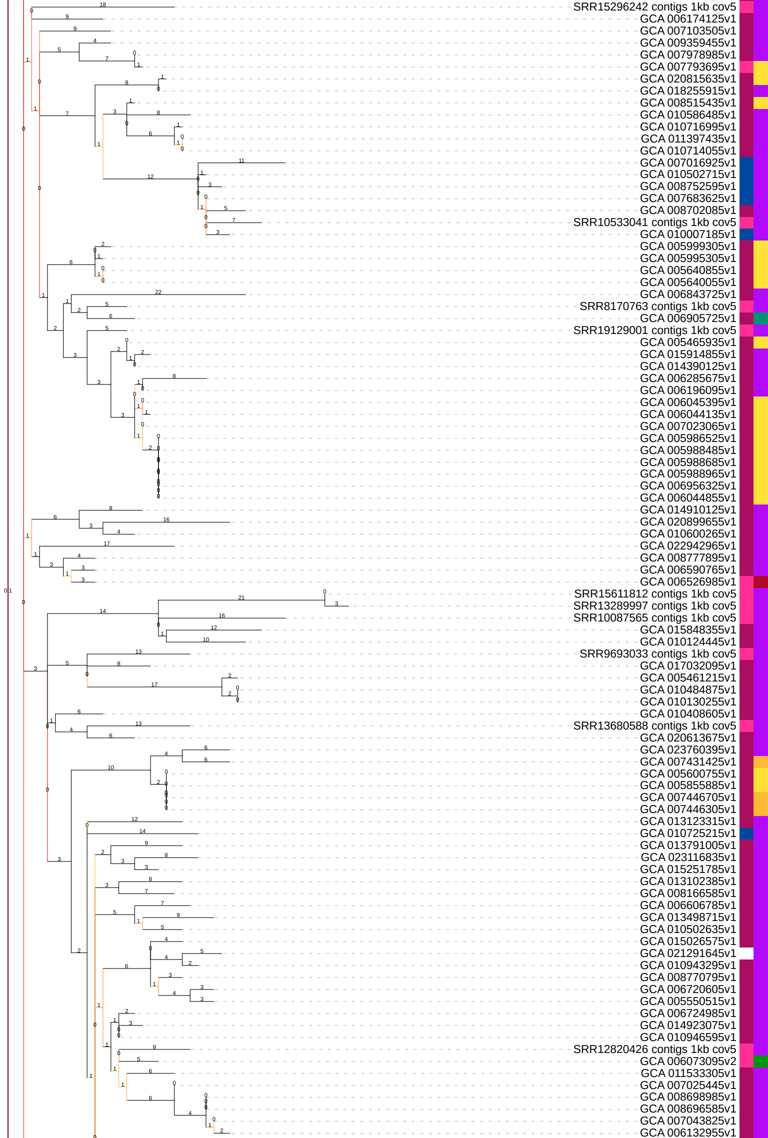
SRR15296242 contigs 1kb cov5
GCA 006174125v1
GCA 007103505v1
GCA 009359455v1
GCA 007978985v1
GCA 007793695v1
GCA 020815635v1
GCA 018255915v1
GCA 008515435v1
GCA 010586485v1
GCA 010716995v1
GCA 011397435v1
GCA 010714055v1
GCA 007016925v1
GCA 010502715v1
GCA 008752595v1
GCA 007683625v1
GCA 008702085v1
SRR10533041 contigs 1kb cov5
GCA 010007185v1
GCA 005999305v1
GCA 005995305v1
GCA 005640855v1
GCA 005640055v1
GCA 006843725v1
SRR8170763 contigs 1kb cov5
GCA 006905725v1
SRR19129001 contigs 1kb cov5
GCA 005465935v1
GCA 015914855v1
GCA 014390125v1
GCA 006285675v1
GCA 006196095v1
GCA 006045395v1
GCA 006044135v1
GCA 007023065v1
GCA 005986525v1
GCA 005988485v1
GCA 005988685v1
GCA 005988965v1
GCA 006956325v1
GCA 006044855v1
GCA 014910125v1
GCA 020899655v1
GCA 010600265v1
GCA 022942965v1
GCA 008777895v1
GCA 006590765v1
GCA 006526985v1
SRR15611812 contigs 1kb cov5
SRR13289997 contigs 1kb cov5
SRR10087565 contigs 1kb cov5
GCA 015848355v1
GCA 010124445v1
SRR9693033 contigs 1kb cov5
GCA 017032095v1
GCA 005461215v1
GCA 010484875v1
GCA 010130255v1
GCA 010408605v1
SRR13680588 contigs 1kb cov5
GCA 020613675v1
GCA 023760395v1
GCA 007431425v1
GCA 005600755v1
GCA 005855885v1
GCA 007446705v1
GCA 007446305v1
GCA 013123315v1
GCA 010725215v1
GCA 013791005v1
GCA 023116835v1
GCA 015251785v1
GCA 013102385v1
GCA 008166585v1
GCA 006606785v1
GCA 013498715v1
GCA 010502635v1
GCA 015026575v1
GCA 021291645v1
GCA 010943295v1
GCA 008770795v1
GCA 006720605v1
GCA 005550515v1
GCA 006724985v1
GCA 014923075v1
GCA 010946595v1
SRR12820426 contigs 1kb cov5
GCA 006073095v2
GCA 011533305v1
GCA 007025445v1
GCA 008698985v1
GCA 008696585v1
GCA 007043825v1
GCA 006132955v1

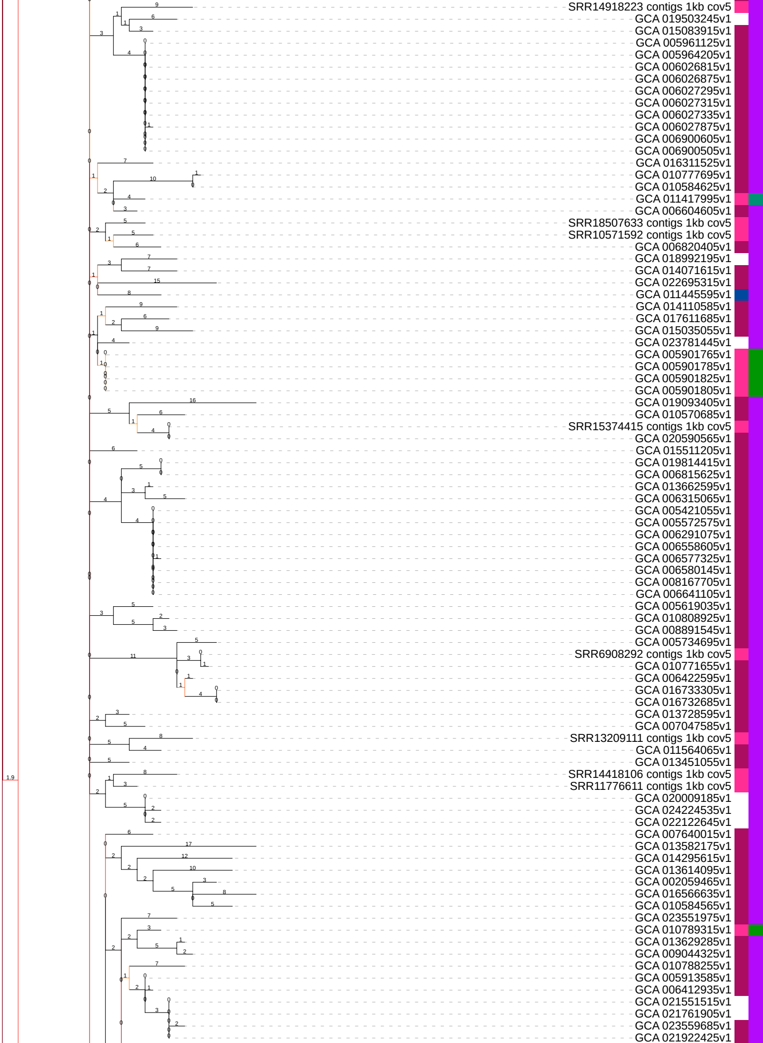

1.9
SRR14918223 contigs 1kb cov5
GCA 019503245v1
GCA 015083915v1
GCA 005961125v1
GCA 005964205v1
GCA 006026815v1
GCA 006026875v1
GCA 006027295v1
GCA 006027315v1
GCA 006027335v1
GCA 006027875v1
GCA 006900605v1
GCA 006900505v1
GCA 016311525v1
GCA 010777695v1
GCA 010584625v1
GCA 011417995v1
GCA 006604605v1
SRR18507633 contigs 1kb cov5
SRR10571592 contigs 1kb cov5
GCA 006820405v1
GCA 018992195v1
GCA 014071615v1
GCA 022695315v1
GCA 011445595v1
GCA 014110585v1
GCA 017611685v1
GCA 015035055v1
GCA 023781445v1
GCA 005901765v1
GCA 005901785v1
GCA 005901825v1
GCA 005901805v1
GCA 019093405v1
GCA 010570685v1
SRR15374415 contigs 1kb cov5
GCA 020590565v1
GCA 015511205v1
GCA 019814415v1
GCA 006815625v1
GCA 013662595v1
GCA 006315065v1
GCA 005421055v1
GCA 005572575v1
GCA 006291075v1
GCA 006558605v1
GCA 006577325v1
GCA 006580145v1
GCA 008167705v1
GCA 006641105v1
GCA 005619035v1
GCA 010808925v1
GCA 008891545v1
GCA 005734695v1
SRR6908292 contigs 1kb cov5
GCA 010771655v1
GCA 006422595v1
GCA 016733305v1
GCA 016732685v1
GCA 013728595v1
GCA 007047585v1
SRR13209111 contigs 1kb cov5
GCA 011564065v1
GCA 013451055v1
SRR14418106 contigs 1kb cov5
SRR11776611 contigs 1kb cov5
GCA 020009185v1
GCA 024224535v1
GCA 022122645v1
GCA 007640015v1
GCA 013582175v1
GCA 014295615v1
GCA 013614095v1
GCA 002059465v1
GCA 016566635v1
GCA 010584565v1
GCA 023551975v1
GCA 010789315v1
GCA 013629285v1
GCA 009044325v1
GCA 010788255v1
GCA 005913585v1
GCA 006412935v1
GCA 021551515v1
GCA 021761905v1
GCA 023559685v1
GCA 021922425v1

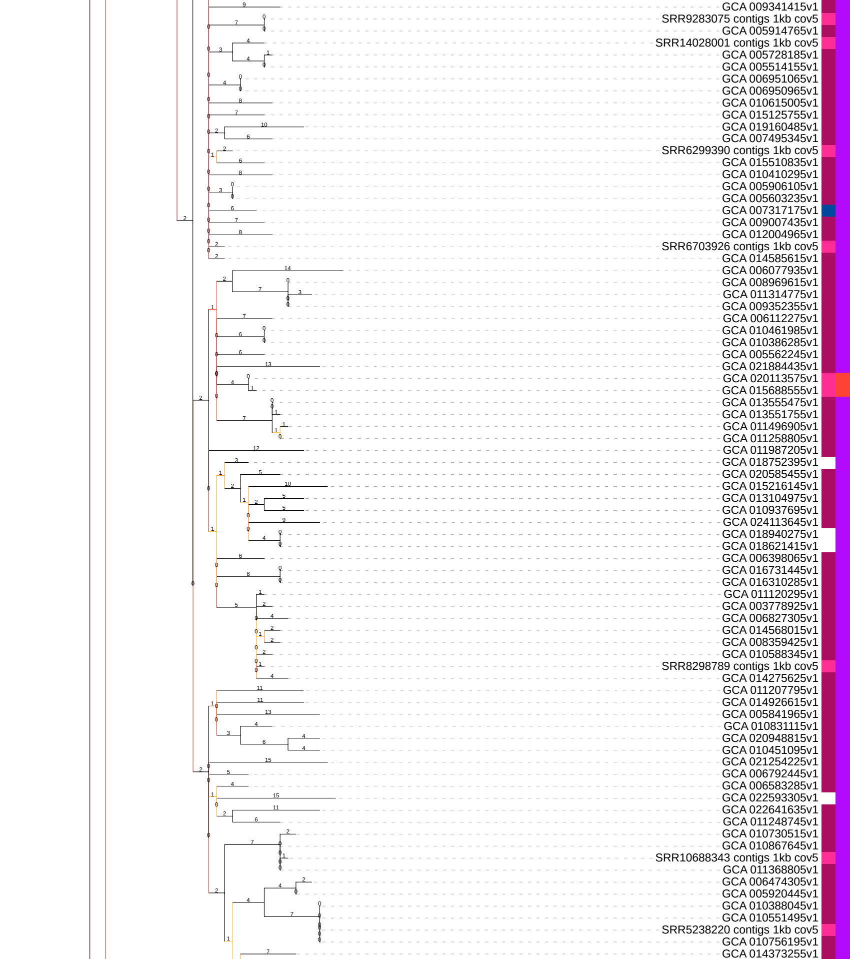
GCA 009341415v1
SRR9283075 contigs 1kb cov5
GCA 005914765v1
SRR14028001 contigs 1kb cov5
GCA 005728185v1
GCA 005514155v1
GCA 006951065v1
GCA 006950965v1
GCA 010615005v1
GCA 015125755v1
GCA 019160485v1
GCA 007495345v1
SRR6299390 contigs 1kb cov5
GCA 015510835v1
GCA 010410295v1
GCA 005906105v1
GCA 005603235v1
GCA 007317175v1
GCA 009007435v1
GCA 012004965v1
SRR6703926 contigs 1kb cov5
GCA 014585615v1
GCA 006077935v1
GCA 008969615v1
GCA 011314775v1
GCA 009352355v1
GCA 006112275v1
GCA 010461985v1
GCA 010386285v1
GCA 005562245v1
GCA 021884435v1
GCA 020113575v1
GCA 015688555v1
GCA 013555475v1
GCA 013551755v1
GCA 011496905v1
GCA 011258805v1
GCA 011987205v1
GCA 018752395v1
GCA 020585455v1
GCA 015216145v1
GCA 013104975v1
GCA 010937695v1
GCA 024113645v1
GCA 018940275v1
GCA 018621415v1
GCA 006398065v1
GCA 016731445v1
GCA 016310285v1
GCA 011120295v1
GCA 003778925v1
GCA 006827305v1
GCA 014568015v1
GCA 008359425v1
GCA 010588345v1
SRR8298789 contigs 1kb cov5
GCA 014275625v1
GCA 011207795v1
GCA 014926615v1
GCA 005841965v1
GCA 010831115v1
GCA 020948815v1
GCA 010451095v1
GCA 021254225v1
GCA 006792445v1
GCA 006583285v1
GCA 022593305v1
GCA 022641635v1
GCA 011248745v1
GCA 010730515v1
GCA 010867645v1
SRR10688343 contigs 1kb cov5
GCA 011368805v1
GCA 006474305v1
GCA 005920445v1
GCA 010388045v1
GCA 010551495v1
SRR5238220 contigs 1kb cov5
GCA 010756195v1
GCA 014373255v1

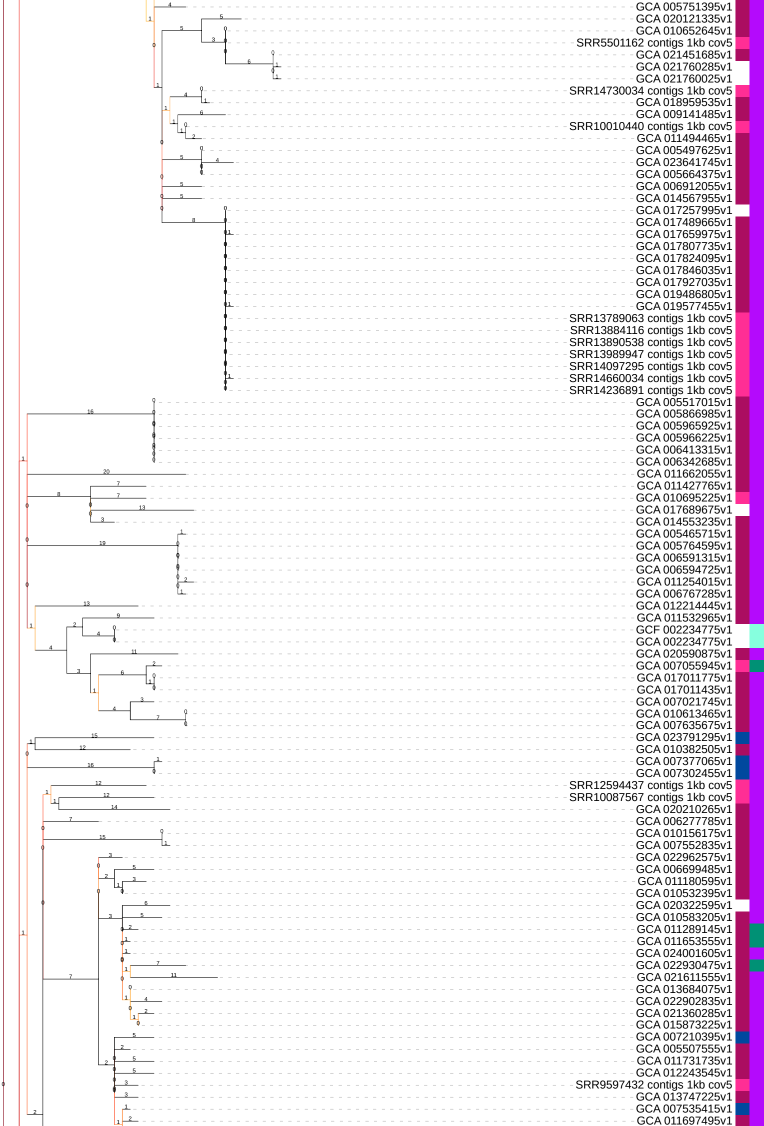
GCA 005751395v1
GCA 020121335v1
GCA 010652645v1
SRR5501162 contigs 1kb cov5
GCA 021451685v1
GCA 021760285v1
GCA 021760025v1
SRR14730034 contigs 1kb cov5
GCA 018959535v1
GCA 009141485v1
SRR10010440 contigs 1kb cov5
GCA 011494465v1
GCA 005497625v1
GCA 023641745v1
GCA 005664375v1
GCA 006912055v1
GCA 014567955v1
GCA 017257995v1
GCA 017489665v1
GCA 017659975v1
GCA 017807735v1
GCA 017824095v1
GCA 017846035v1
GCA 017927035v1
GCA 019486805v1
GCA 019577455v1
SRR13789063 contigs 1kb cov5
SRR13884116 contigs 1kb cov5
SRR13890538 contigs 1kb cov5
SRR13989947 contigs 1kb cov5
SRR14097295 contigs 1kb cov5
SRR14660034 contigs 1kb cov5
SRR14236891 contigs 1kb cov5
GCA 005517015v1
GCA 005866985v1
GCA 005965925v1
GCA 005966225v1
GCA 006413315v1
GCA 006342685v1
GCA 011662055v1
GCA 011427765v1
GCA 010695225v1
GCA 017689675v1
GCA 014553235v1
GCA 005465715v1
GCA 005764595v1
GCA 006591315v1
GCA 006594725v1
GCA 011254015v1
GCA 006767285v1
GCA 012214445v1
GCA 011532965v1
GCF 002234775v1
GCA 002234775v1
GCA 020590875v1
GCA 007055945v1
GCA 017011775v1
GCA 017011435v1
GCA 007021745v1
GCA 010613465v1
GCA 007635675v1
GCA 023791295v1
GCA 010382505v1
GCA 007377065v1
GCA 007302455v1
SRR12594437 contigs 1kb cov5
SRR10087567 contigs 1kb cov5
GCA 020210265v1
GCA 006277785v1
GCA 010156175v1
GCA 007552835v1
GCA 022962575v1
GCA 006699485v1
GCA 011180595v1
GCA 010532395v1
GCA 020322595v1
GCA 010583205v1
GCA 011289145v1
GCA 011653555v1
GCA 024001605v1
GCA 022930475v1
GCA 021611555v1
GCA 013684075v1
GCA 022902835v1
GCA 021360285v1
GCA 015873225v1
GCA 007210395v1
GCA 005507555v1
GCA 011731735v1
GCA 012243545v1
SRR9597432 contigs 1kb cov5
GCA 013747225v1
GCA 007535415v1
GCA 011697495v1

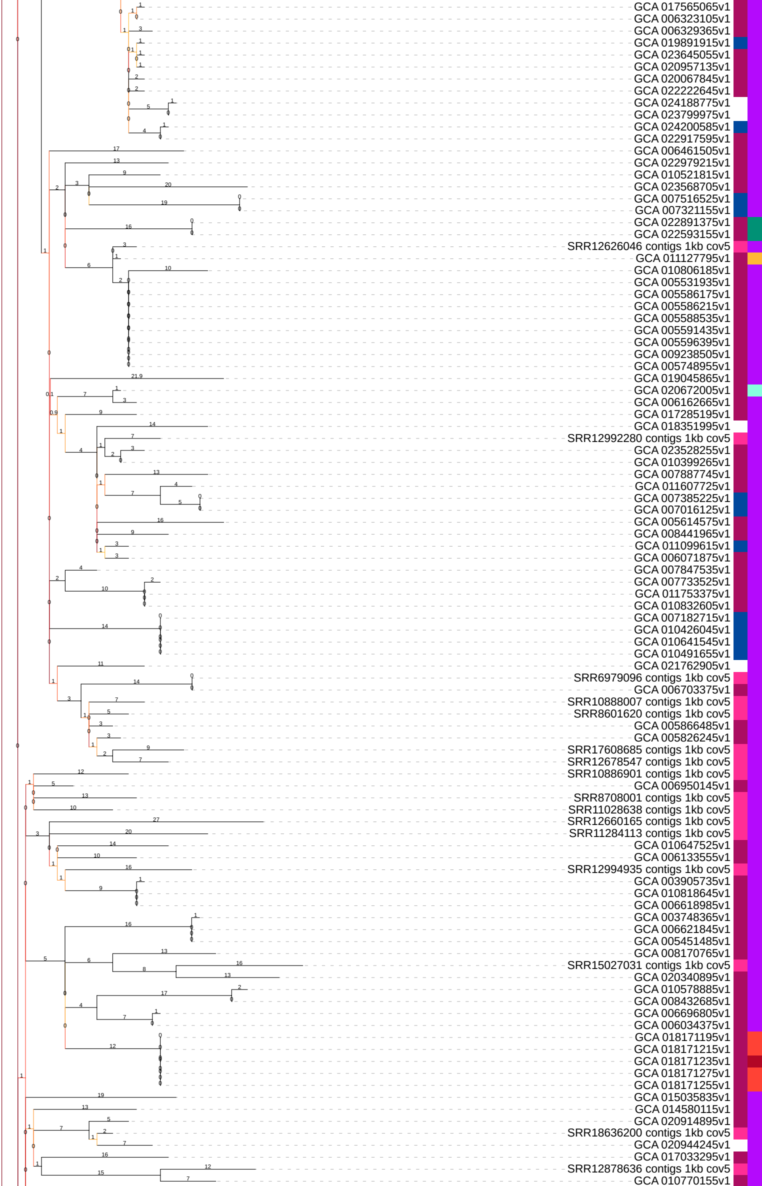
GCA 017565065v1
GCA 006323105v1
GCA 006329365v1
GCA 019891915v1
GCA 023645055v1
GCA 020957135v1
GCA 020067845v1
GCA 022222645v1
GCA 024188775v1
GCA 023799975v1
GCA 024200585v1
GCA 022917595v1
GCA 006461505v1
GCA 022979215v1
GCA 010521815v1
GCA 023568705v1
GCA 007516525v1
GCA 007321155v1
GCA 022891375v1
GCA 022593155v1
SRR12626046 contigs 1kb cov5
GCA 011127795v1
GCA 010806185v1
GCA 005531935v1
GCA 005586175v1
GCA 005586215v1
GCA 005588535v1
GCA 005591435v1
GCA 005596395v1
GCA 009238505v1
GCA 005748955v1
GCA 019045865v1
GCA 020672005v1
GCA 006162665v1
GCA 017285195v1
GCA 018351995v1
SRR12992280 contigs 1kb cov5
GCA 023528255v1
GCA 010399265v1
GCA 007887745v1
GCA 011607725v1
GCA 007385225v1
GCA 007016125v1
GCA 005614575v1
GCA 008441965v1
GCA 011099615v1
GCA 006071875v1
GCA 007847535v1
GCA 007733525v1
GCA 011753375v1
GCA 010832605v1
GCA 007182715v1
GCA 010426045v1
GCA 010641545v1
GCA 010491655v1
GCA 021762905v1
SRR6979096 contigs 1kb cov5
GCA 006703375v1
SRR10888007 contigs 1kb cov5
SRR8601620 contigs 1kb cov5
GCA 005866485v1
GCA 005826245v1
SRR17608685 contigs 1kb cov5
SRR12678547 contigs 1kb cov5
SRR10886901 contigs 1kb cov5
GCA 006950145v1
SRR8708001 contigs 1kb cov5
SRR11028638 contigs 1kb cov5
SRR12660165 contigs 1kb cov5
SRR11284113 contigs 1kb cov5
GCA 010647525v1
GCA 006133555v1
SRR12994935 contigs 1kb cov5
GCA 003905735v1
GCA 010818645v1
GCA 006618985v1
GCA 003748365v1
GCA 006621845v1
GCA 005451485v1
GCA 008170765v1
SRR15027031 contigs 1kb cov5
GCA 020340895v1
GCA 010578885v1
GCA 008432685v1
GCA 006696805v1
GCA 006034375v1
GCA 018171195v1
GCA 018171215v1
GCA 018171235v1
GCA 018171275v1
GCA 018171255v1
GCA 015035835v1
GCA 014580115v1
GCA 020914895v1
SRR18636200 contigs 1kb cov5
GCA 020944245v1
GCA 017033295v1
SRR12878636 contigs 1kb cov5
GCA 010770155v1

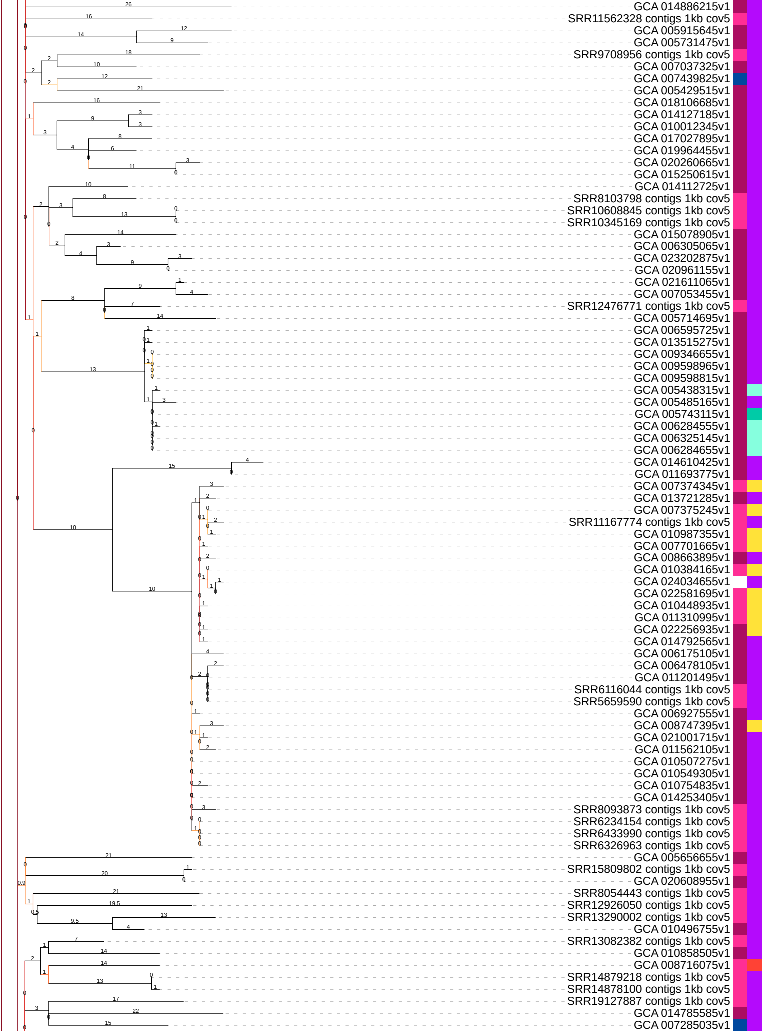
GCA 014886215v1
SRR11562328 contigs 1kb cov5
GCA 005915645v1
GCA 005731475v1
SRR9708956 contigs 1kb cov5
GCA 007037325v1
GCA 007439825v1
GCA 005429515v1
GCA 018106685v1
GCA 014127185v1
GCA 010012345v1
GCA 017027895v1
GCA 019964455v1
GCA 020260665v1
GCA 015250615v1
GCA 014112725v1
SRR8103798 contigs 1kb cov5
SRR10608845 contigs 1kb cov5
SRR10345169 contigs 1kb cov5
GCA 015078905v1
GCA 006305065v1
GCA 023202875v1
GCA 020961155v1
GCA 021611065v1
GCA 007053455v1
SRR12476771 contigs 1kb cov5
GCA 005714695v1
GCA 006595725v1
GCA 013515275v1
GCA 009346655v1
GCA 009598965v1
GCA 009598815v1
GCA 005438315v1
GCA 005485165v1
GCA 005743115v1
GCA 006284555v1
GCA 006325145v1
GCA 006284655v1
GCA 014610425v1
GCA 011693775v1
GCA 007374345v1
GCA 013721285v1
GCA 007375245v1
SRR11167774 contigs 1kb cov5
GCA 010987355v1
GCA 007701665v1
GCA 008663895v1
GCA 010384165v1
GCA 024034655v1
GCA 022581695v1
GCA 010448935v1
GCA 011310995v1
GCA 022256935v1
GCA 014792565v1
GCA 006175105v1
GCA 006478105v1
GCA 011201495v1
SRR6116044 contigs 1kb cov5
SRR5659590 contigs 1kb cov5
GCA 006927555v1
GCA 008747395v1
GCA 021001715v1
GCA 011562105v1
GCA 010507275v1
GCA 010549305v1
GCA 010754835v1
GCA 014253405v1
SRR8093873 contigs 1kb cov5
SRR6234154 contigs 1kb cov5
SRR6433990 contigs 1kb cov5
SRR6326963 contigs 1kb cov5
GCA 005656655v1
SRR15809802 contigs 1kb cov5
GCA 020608955v1
SRR8054443 contigs 1kb cov5
SRR12926050 contigs 1kb cov5
SRR13290002 contigs 1kb cov5
GCA 010496755v1
SRR13082382 contigs 1kb cov5
GCA 010858505v1
GCA 008716075v1
SRR14879218 contigs 1kb cov5
SRR14878100 contigs 1kb cov5
SRR19127887 contigs 1kb cov5
GCA 014785585v1
GCA 007285035v1

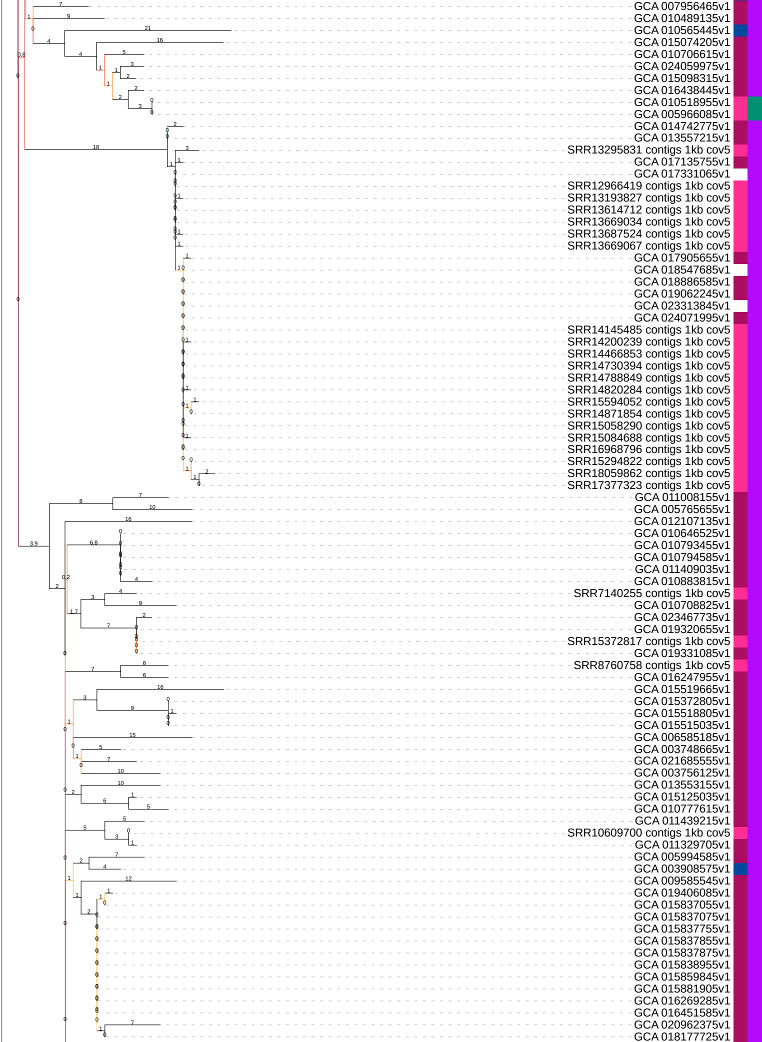

GCA 007956465v1
GCA 010489135v1
GCA 010565445v1
GCA 015074205v1
GCA 010706615v1
GCA 024059975v1
GCA 015098315v1
GCA 016438445v1
GCA 010518955v1
GCA 005966085v1
GCA 014742775v1
GCA 013557215v1
SRR13295831 contigs 1kb cov5
GCA 017135755v1
GCA 017331065v1
SRR12966419 contigs 1kb cov5
SRR13193827 contigs 1kb cov5
SRR13614712 contigs 1kb cov5
SRR13669034 contigs 1kb cov5
SRR13687524 contigs 1kb cov5
SRR13669067 contigs 1kb cov5
GCA 017905655v1
GCA 018547685v1
GCA 018886585v1
GCA 019062245v1
GCA 023313845v1
GCA 024071995v1
SRR14145485 contigs 1kb cov5
SRR14200239 contigs 1kb cov5
SRR14466853 contigs 1kb cov5
SRR14730394 contigs 1kb cov5
SRR14788849 contigs 1kb cov5
SRR14820284 contigs 1kb cov5
SRR15594052 contigs 1kb cov5
SRR14871854 contigs 1kb cov5
SRR15058290 contigs 1kb cov5
SRR15084688 contigs 1kb cov5
SRR16968796 contigs 1kb cov5
SRR15294822 contigs 1kb cov5
SRR18059862 contigs 1kb cov5
SRR17377323 contigs 1kb cov5
GCA 011008155v1
GCA 005765655v1
GCA 012107135v1
GCA 010646525v1
GCA 010793455v1
GCA 010794585v1
GCA 011409035v1
GCA 010883815v1
SRR7140255 contigs 1kb cov5
GCA 010708825v1
GCA 023467735v1
GCA 019320655v1
SRR15372817 contigs 1kb cov5
GCA 019331085v1
SRR8760758 contigs 1kb cov5
GCA 016247955v1
GCA 015519665v1
GCA 015372805v1
GCA 015518805v1
GCA 015515035v1
GCA 006585185v1
GCA 003748665v1
GCA 021685555v1
GCA 003756125v1
GCA 013553155v1
GCA 015125035v1
GCA 010777615v1
GCA 011439215v1
SRR10609700 contigs 1kb cov5
GCA 011329705v1
GCA 005994585v1
GCA 003908575v1
GCA 009585545v1
GCA 019406085v1
GCA 015837055v1
GCA 015837075v1
GCA 015837755v1
GCA 015837855v1
GCA 015837875v1
GCA 015838955v1
GCA 015859845v1
GCA 015881905v1
GCA 016269285v1
GCA 016451585v1
GCA 020962375v1
GCA 018177725v1

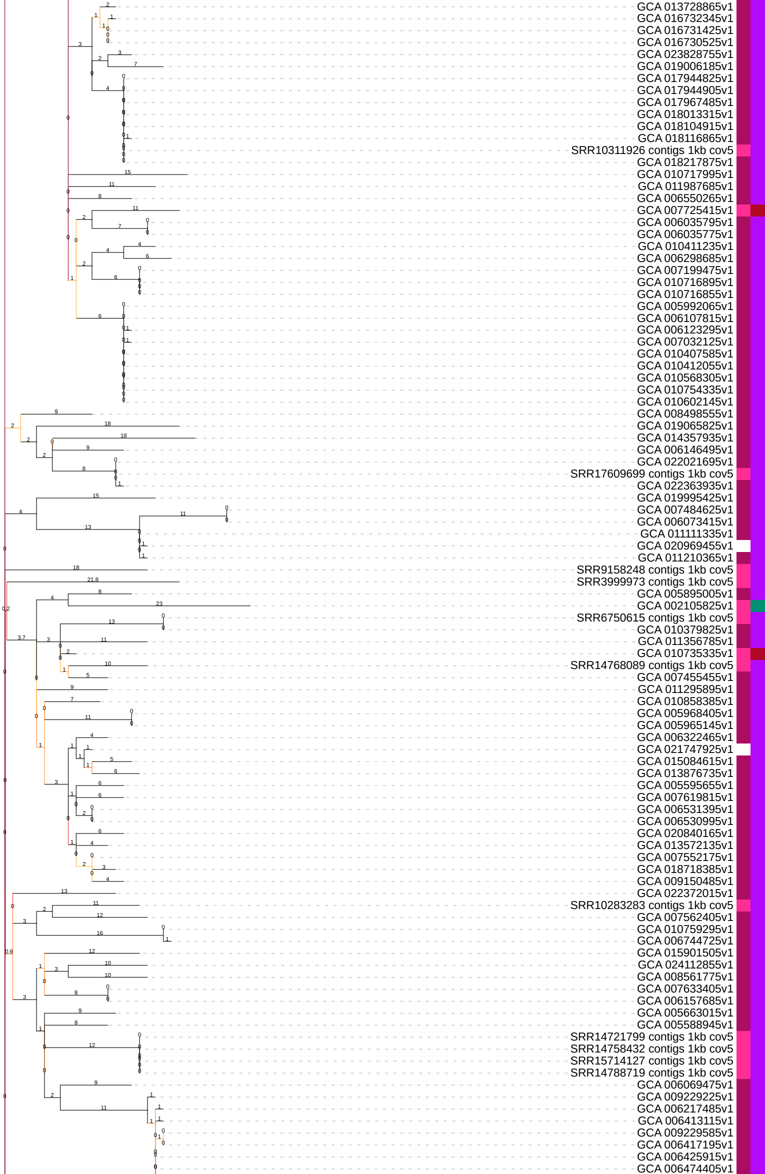
GCA 013728865v1
GCA 016732345v1
GCA 016731425v1
GCA 016730525v1
GCA 023828755v1
GCA 019006185v1
GCA 017944825v1
GCA 017944905v1
GCA 017967485v1
GCA 018013315v1
GCA 018104915v1
GCA 018116865v1
SRR10311926 contigs 1kb cov5
GCA 018217875v1
GCA 010717995v1
GCA 011987685v1
GCA 006550265v1
GCA 007725415v1
GCA 006035795v1
GCA 006035775v1
GCA 010411235v1
GCA 006298685v1
GCA 007199475v1
GCA 010716895v1
GCA 010716855v1
GCA 005992065v1
GCA 006107815v1
GCA 006123295v1
GCA 007032125v1
GCA 010407585v1
GCA 010412055v1
GCA 010568305v1
GCA 010754335v1
GCA 010602145v1
GCA 008498555v1
GCA 019065825v1
GCA 014357935v1
GCA 006146495v1
GCA 022021695v1
SRR17609699 contigs 1kb cov5
GCA 022363935v1
GCA 019995425v1
GCA 007484625v1
GCA 006073415v1
GCA 011111335v1
GCA 020969455v1
GCA 011210365v1
SRR9158248 contigs 1kb cov5
SRR3999973 contigs 1kb cov5
GCA 005895005v1
GCA 002105825v1
SRR6750615 contigs 1kb cov5
GCA 010379825v1
GCA 011356785v1
GCA 010735335v1
SRR14768089 contigs 1kb cov5
GCA 007455455v1
GCA 011295895v1
GCA 010858385v1
GCA 005968405v1
GCA 005965145v1
GCA 006322465v1
GCA 021747925v1
GCA 015084615v1
GCA 013876735v1
GCA 005595655v1
GCA 007619815v1
GCA 006531395v1
GCA 006530995v1
GCA 020840165v1
GCA 013572135v1
GCA 007552175v1
GCA 018718385v1
GCA 009150485v1
GCA 022372015v1
SRR10283283 contigs 1kb cov5
GCA 007562405v1
GCA 010759295v1
GCA 006744725v1
GCA 015901505v1
GCA 024112855v1
GCA 008561775v1
GCA 007633405v1
GCA 006157685v1
GCA 005663015v1
GCA 005588945v1
SRR14721799 contigs 1kb cov5
SRR14758432 contigs 1kb cov5
SRR15714127 contigs 1kb cov5
SRR14788719 contigs 1kb cov5
GCA 006069475v1
GCA 009229225v1
GCA 006217485v1
GCA 006413115v1
GCA 009229585v1
GCA 006417195v1
GCA 006425915v1
GCA 006474405v1

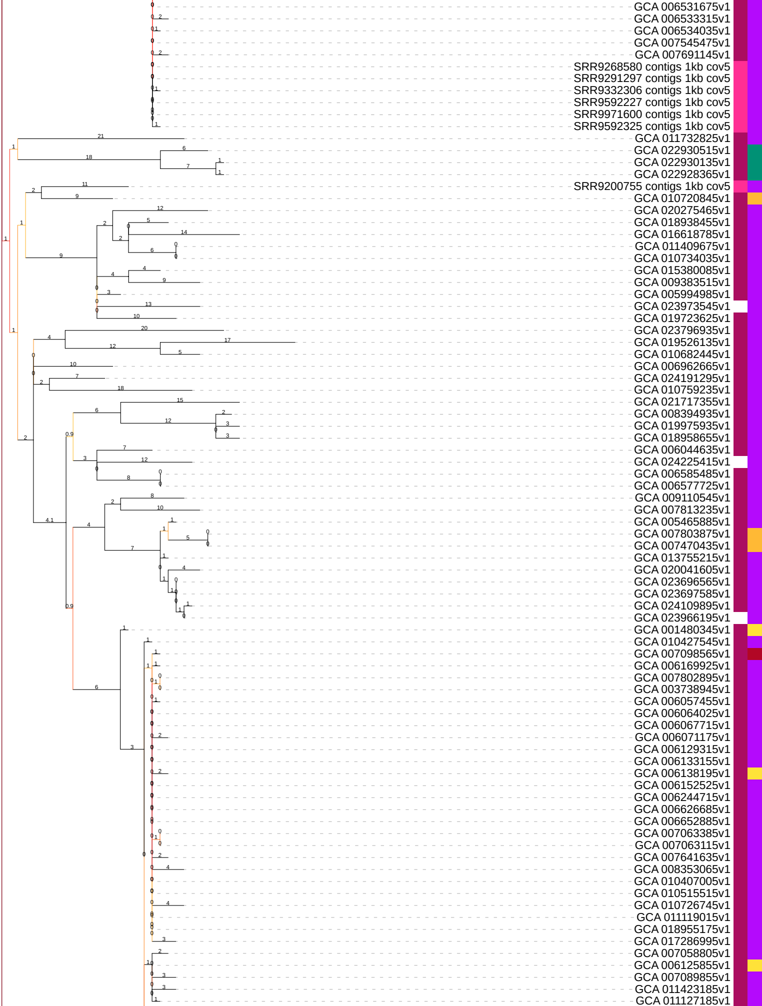
GCA 006531675v1
GCA 006533315v1
GCA 006534035v1
GCA 007545475v1
GCA 007691145v1
SRR9268580 contigs 1kb cov5
SRR9291297 contigs 1kb cov5
SRR9332306 contigs 1kb cov5
SRR9592227 contigs 1kb cov5
SRR9971600 contigs 1kb cov5
SRR9592325 contigs 1kb cov5
GCA 011732825v1
GCA 022930515v1
GCA 022930135v1
GCA 022928365v1
SRR9200755 contigs 1kb cov5
GCA 010720845v1
GCA 020275465v1
GCA 018938455v1
GCA 016618785v1
GCA 011409675v1
GCA 010734035v1
GCA 015380085v1
GCA 009383515v1
GCA 005994985v1
GCA 023973545v1
GCA 019723625v1
GCA 023796935v1
GCA 019526135v1
GCA 010682445v1
GCA 006962665v1
GCA 024191295v1
GCA 010759235v1
GCA 021717355v1
GCA 008394935v1
GCA 019975935v1
GCA 018958655v1
GCA 006044635v1
GCA 024225415v1
GCA 006585485v1
GCA 006577725v1
GCA 009110545v1
GCA 007813235v1
GCA 005465885v1
GCA 007803875v1
GCA 007470435v1
GCA 013755215v1
GCA 020041605v1
GCA 023696565v1
GCA 023697585v1
GCA 024109895v1
GCA 023966195v1
GCA 001480345v1
GCA 010427545v1
GCA 007098565v1
GCA 006169925v1
GCA 007802895v1
GCA 003738945v1
GCA 006057455v1
GCA 006064025v1
GCA 006067715v1
GCA 006071175v1
GCA 006129315v1
GCA 006133155v1
GCA 006138195v1
GCA 006152525v1
GCA 006244715v1
GCA 006626685v1
GCA 006652885v1
GCA 007063385v1
GCA 007063115v1
GCA 007641635v1
GCA 008353065v1
GCA 010407005v1
GCA 010515515v1
GCA 010726745v1
GCA 011119015v1
GCA 018955175v1
GCA 017286995v1
GCA 007058805v1
GCA 006125855v1
GCA 007089855v1
GCA 011423185v1
GCA 011127185v1

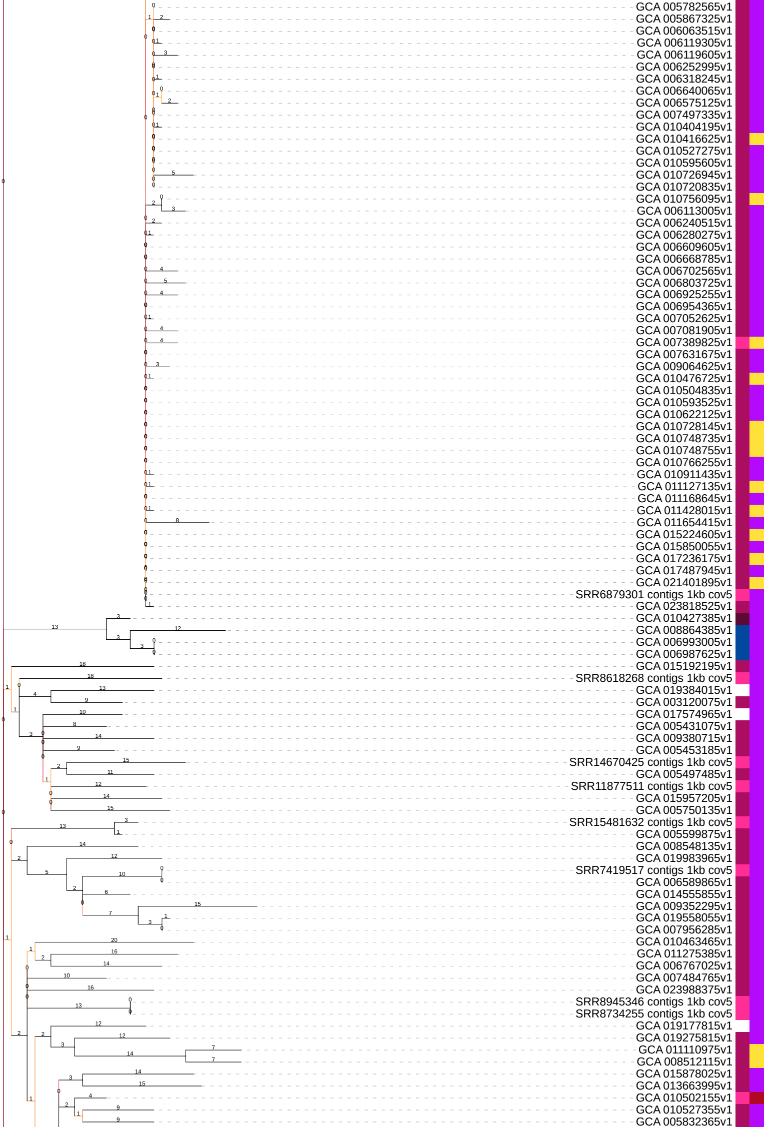
GCA 005782565v1
GCA 005867325v1
GCA 006063515v1
GCA 006119305v1
GCA 006119605v1
GCA 006252995v1
GCA 006318245v1
GCA 006640065v1
GCA 006575125v1
GCA 007497335v1
GCA 010404195v1
GCA 010416625v1
GCA 010527275v1
GCA 010595605v1
GCA 010726945v1
GCA 010720835v1
GCA 010756095v1
GCA 006113005v1
GCA 006240515v1
GCA 006280275v1
GCA 006609605v1
GCA 006668785v1
GCA 006702565v1
GCA 006803725v1
GCA 006925255v1
GCA 006954365v1
GCA 007052625v1
GCA 007081905v1
GCA 007389825v1
GCA 007631675v1
GCA 009064625v1
GCA 010476725v1
GCA 010504835v1
GCA 010593525v1
GCA 010622125v1
GCA 010728145v1
GCA 010748735v1
GCA 010748755v1
GCA 010766255v1
GCA 010911435v1
GCA 011127135v1
GCA 011168645v1
GCA 011428015v1
GCA 011654415v1
GCA 015224605v1
GCA 015850055v1
GCA 017236175v1
GCA 017487945v1
GCA 021401895v1
SRR6879301 contigs 1kb cov5
GCA 023818525v1
GCA 010427385v1
GCA 008864385v1
GCA 006993005v1
GCA 006987625v1
GCA 015192195v1
SRR8618268 contigs 1kb cov5
GCA 019384015v1
GCA 003120075v1
GCA 017574965v1
GCA 005431075v1
GCA 009380715v1
GCA 005453185v1
SRR14670425 contigs 1kb cov5
GCA 005497485v1
SRR11877511 contigs 1kb cov5
GCA 015957205v1
GCA 005750135v1
SRR15481632 contigs 1kb cov5
GCA 005599875v1
GCA 008548135v1
GCA 019983965v1
SRR7419517 contigs 1kb cov5
GCA 006589865v1
GCA 014555855v1
GCA 009352295v1
GCA 019558055v1
GCA 007956285v1
GCA 010463465v1
GCA 011275385v1
GCA 006767025v1
GCA 007484765v1
GCA 023988375v1
SRR8945346 contigs 1kb cov5
SRR8734255 contigs 1kb cov5
GCA 019177815v1
GCA 019275815v1
GCA 011110975v1
GCA 008512115v1
GCA 015878025v1
GCA 013663995v1
GCA 010502155v1
GCA 010527355v1
GCA 005832365v1

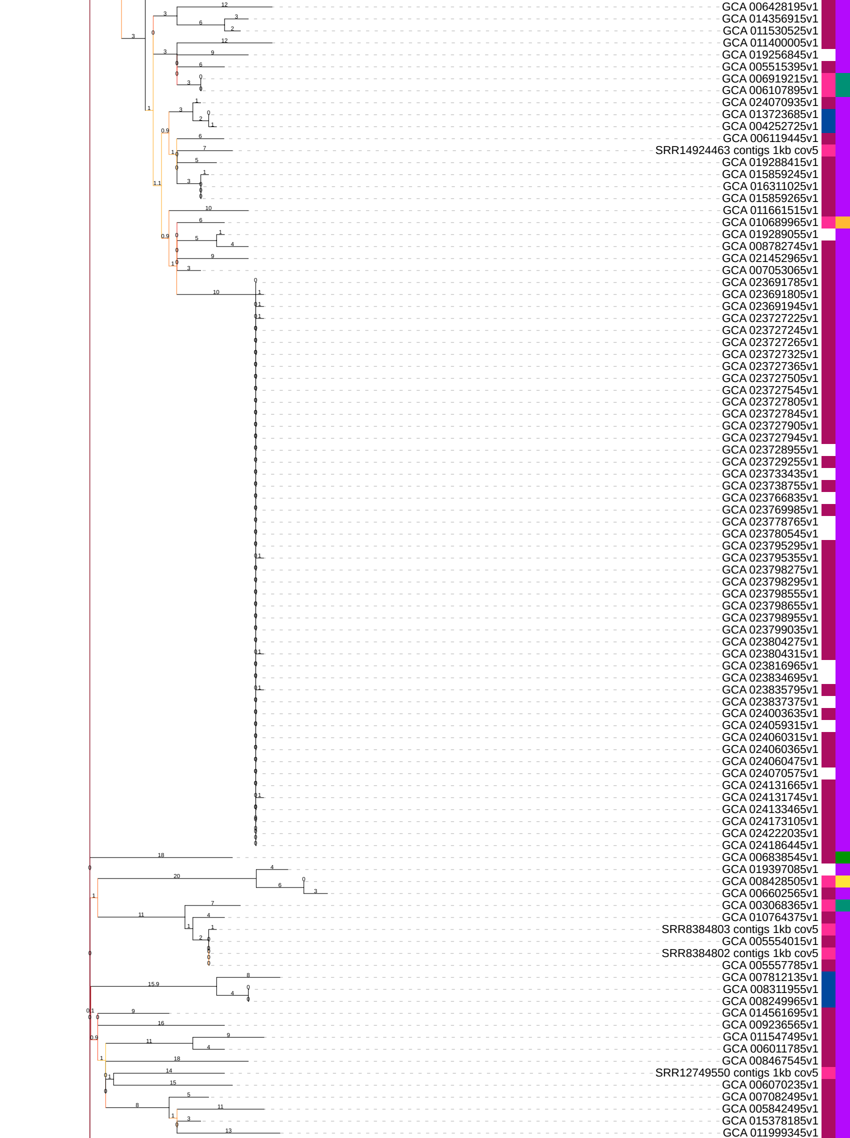
GCA 006428195v1
GCA 014356915v1
GCA 011530525v1
GCA 011400005v1
GCA 019256845v1
GCA 005515395v1
GCA 006919215v1
GCA 006107895v1
GCA 024070935v1
GCA 013723685v1
GCA 004252725v1
GCA 006119445v1
SRR14924463 contigs 1kb cov5
GCA 019288415v1
GCA 015859245v1
GCA 016311025v1
GCA 015859265v1
GCA 011661515v1
GCA 010689965v1
GCA 019289055v1
GCA 008782745v1
GCA 021452965v1
GCA 007053065v1
GCA 023691785v1
GCA 023691805v1
GCA 023691945v1
GCA 023727225v1
GCA 023727245v1
GCA 023727265v1
GCA 023727325v1
GCA 023727365v1
GCA 023727505v1
GCA 023727545v1
GCA 023727805v1
GCA 023727845v1
GCA 023727905v1
GCA 023727945v1
GCA 023728955v1
GCA 023729255v1
GCA 023733435v1
GCA 023738755v1
GCA 023766835v1
GCA 023769985v1
GCA 023778765v1
GCA 023780545v1
GCA 023795295v1
GCA 023795355v1
GCA 023798275v1
GCA 023798295v1
GCA 023798555v1
GCA 023798655v1
GCA 023798955v1
GCA 023799035v1
GCA 023804275v1
GCA 023804315v1
GCA 023816965v1
GCA 023834695v1
GCA 023835795v1
GCA 023837375v1
GCA 024003635v1
GCA 024059315v1
GCA 024060315v1
GCA 024060365v1
GCA 024060475v1
GCA 024070575v1
GCA 024131665v1
GCA 024131745v1
GCA 024133465v1
GCA 024173105v1
GCA 024222035v1
GCA 024186445v1
GCA 006838545v1
GCA 019397085v1
GCA 008428505v1
GCA 006602565v1
GCA 003068365v1
GCA 010764375v1
SRR8384803 contigs 1kb cov5
GCA 005554015v1
SRR8384802 contigs 1kb cov5
GCA 005557785v1
GCA 007812135v1
GCA 008311955v1
GCA 008249965v1
GCA 014561695v1
GCA 009236565v1
GCA 011547495v1
GCA 006011785v1
GCA 008467545v1
SRR12749550 contigs 1kb cov5
GCA 006070235v1
GCA 007082495v1
GCA 005842495v1
GCA 015378185v1
GCA 011999345v1

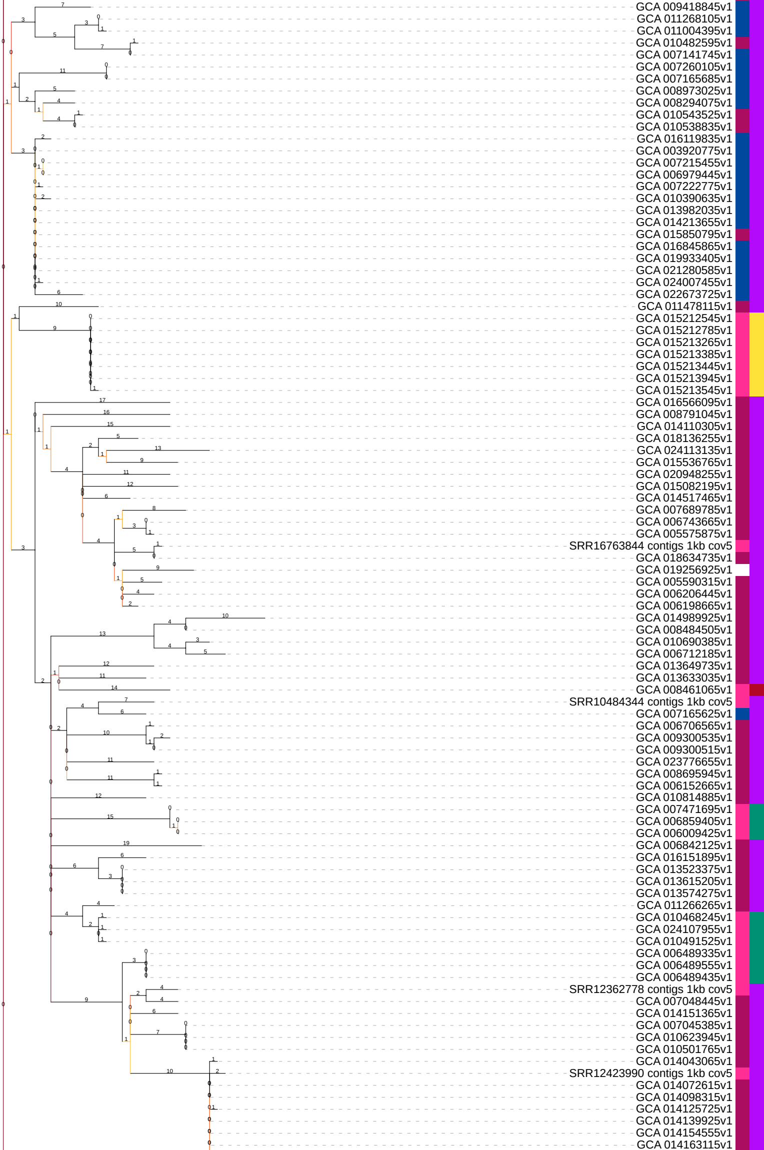
GCA 009418845v1
GCA 011268105v1
GCA 011004395v1
GCA 010482595v1
GCA 007141745v1
GCA 007260105v1
GCA 007165685v1
GCA 008973025v1
GCA 008294075v1
GCA 010543525v1
GCA 010538835v1
GCA 016119835v1
GCA 003920775v1
GCA 007215455v1
GCA 006979445v1
GCA 007222775v1
GCA 010390635v1
GCA 013982035v1
GCA 014213655v1
GCA 015850795v1
GCA 016845865v1
GCA 019933405v1
GCA 021280585v1
GCA 024007455v1
GCA 022673725v1
GCA 011478115v1
GCA 015212545v1
GCA 015212785v1
GCA 015213265v1
GCA 015213385v1
GCA 015213445v1
GCA 015213945v1
GCA 015213545v1
GCA 016566095v1
GCA 008791045v1
GCA 014110305v1
GCA 018136255v1
GCA 024113135v1
GCA 015536765v1
GCA 020948255v1
GCA 015082195v1
GCA 014517465v1
GCA 007689785v1
GCA 006743665v1
GCA 005575875v1
SRR16763844 contigs 1kb cov5
GCA 018634735v1
GCA 019256925v1
GCA 005590315v1
GCA 006206445v1
GCA 006198665v1
GCA 014989925v1
GCA 008484505v1
GCA 010690385v1
GCA 006712185v1
GCA 013649735v1
GCA 013633035v1
GCA 008461065v1
SRR10484344 contigs 1kb cov5
GCA 007165625v1
GCA 006706565v1
GCA 009300535v1
GCA 009300515v1
GCA 023776655v1
GCA 008695945v1
GCA 006152665v1
GCA 010814885v1
GCA 007471695v1
GCA 006859405v1
GCA 006009425v1
GCA 006842125v1
GCA 016151895v1
GCA 013523375v1
GCA 013615205v1
GCA 013574275v1
GCA 011266265v1
GCA 010468245v1
GCA 024107955v1
GCA 010491525v1
GCA 006489335v1
GCA 006489555v1
GCA 006489435v1
SRR12362778 contigs 1kb cov5
GCA 007048445v1
GCA 014151365v1
GCA 007045385v1
GCA 010623945v1
GCA 010501765v1
GCA 014043065v1
SRR12423990 contigs 1kb cov5
GCA 014072615v1
GCA 014098315v1
GCA 014125725v1
GCA 014139925v1
GCA 014154555v1
GCA 014163115v1

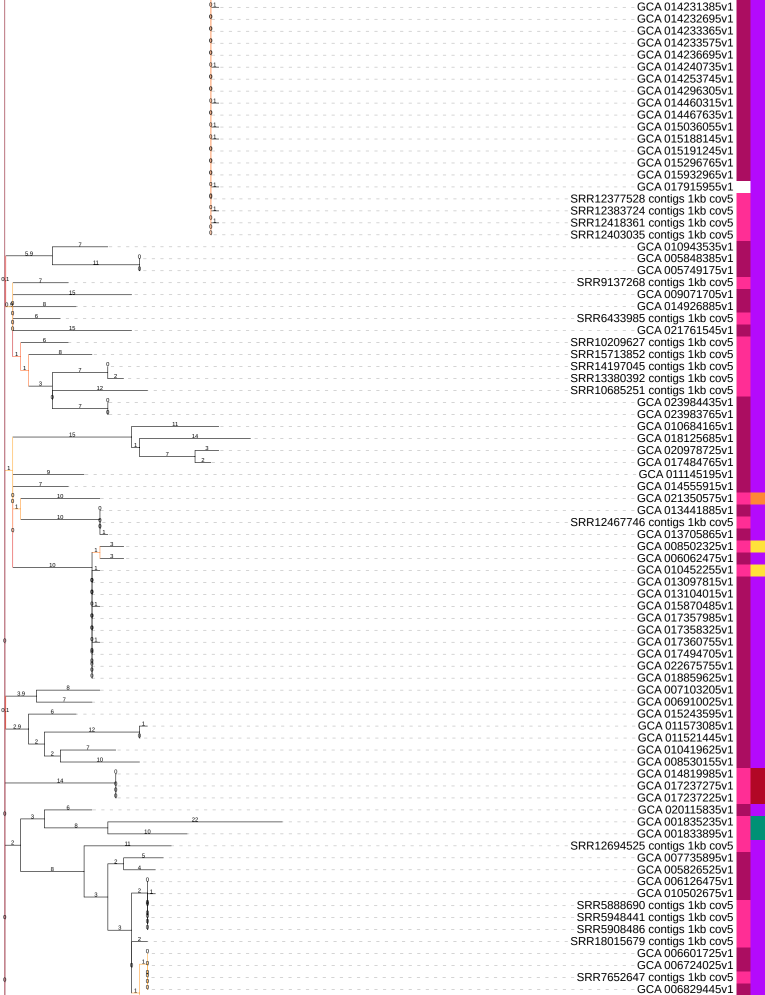
GCA 014231385v1
GCA 014232695v1
GCA 014233365v1
GCA 014233575v1
GCA 014236695v1
GCA 014240735v1
GCA 014253745v1
GCA 014296305v1
GCA 014460315v1
GCA 014467635v1
GCA 015036055v1
GCA 015188145v1
GCA 015191245v1
GCA 015296765v1
GCA 015932965v1
GCA 017915955v1
SRR12377528 contigs 1kb cov5
SRR12383724 contigs 1kb cov5
SRR12418361 contigs 1kb cov5
SRR12403035 contigs 1kb cov5
GCA 010943535v1
GCA 005848385v1
GCA 005749175v1
SRR9137268 contigs 1kb cov5
GCA 009071705v1
GCA 014926885v1
SRR6433985 contigs 1kb cov5
GCA 021761545v1
SRR10209627 contigs 1kb cov5
SRR15713852 contigs 1kb cov5
SRR14197045 contigs 1kb cov5
SRR13380392 contigs 1kb cov5
SRR10685251 contigs 1kb cov5
GCA 023984435v1
GCA 023983765v1
GCA 010684165v1
GCA 018125685v1
GCA 020978725v1
GCA 017484765v1
GCA 011145195v1
GCA 014555915v1
GCA 021350575v1
GCA 013441885v1
SRR12467746 contigs 1kb cov5
GCA 013705865v1
GCA 008502325v1
GCA 006062475v1
GCA 010452255v1
GCA 013097815v1
GCA 013104015v1
GCA 015870485v1
GCA 017357985v1
GCA 017358325v1
GCA 017360755v1
GCA 017494705v1
GCA 022675755v1
GCA 018859625v1
GCA 007103205v1
GCA 006910025v1
GCA 015243595v1
GCA 011573085v1
GCA 011521445v1
GCA 010419625v1
GCA 008530155v1
GCA 014819985v1
GCA 017237275v1
GCA 017237225v1
GCA 020115835v1
GCA 001835235v1
GCA 001833895v1
SRR12694525 contigs 1kb cov5
GCA 007735895v1
GCA 005826525v1
GCA 006126475v1
GCA 010502675v1
SRR5888690 contigs 1kb cov5
SRR5948441 contigs 1kb cov5
SRR5908486 contigs 1kb cov5
SRR18015679 contigs 1kb cov5
GCA 006601725v1
GCA 006724025v1
SRR7652647 contigs 1kb cov5
GCA 006829445v1

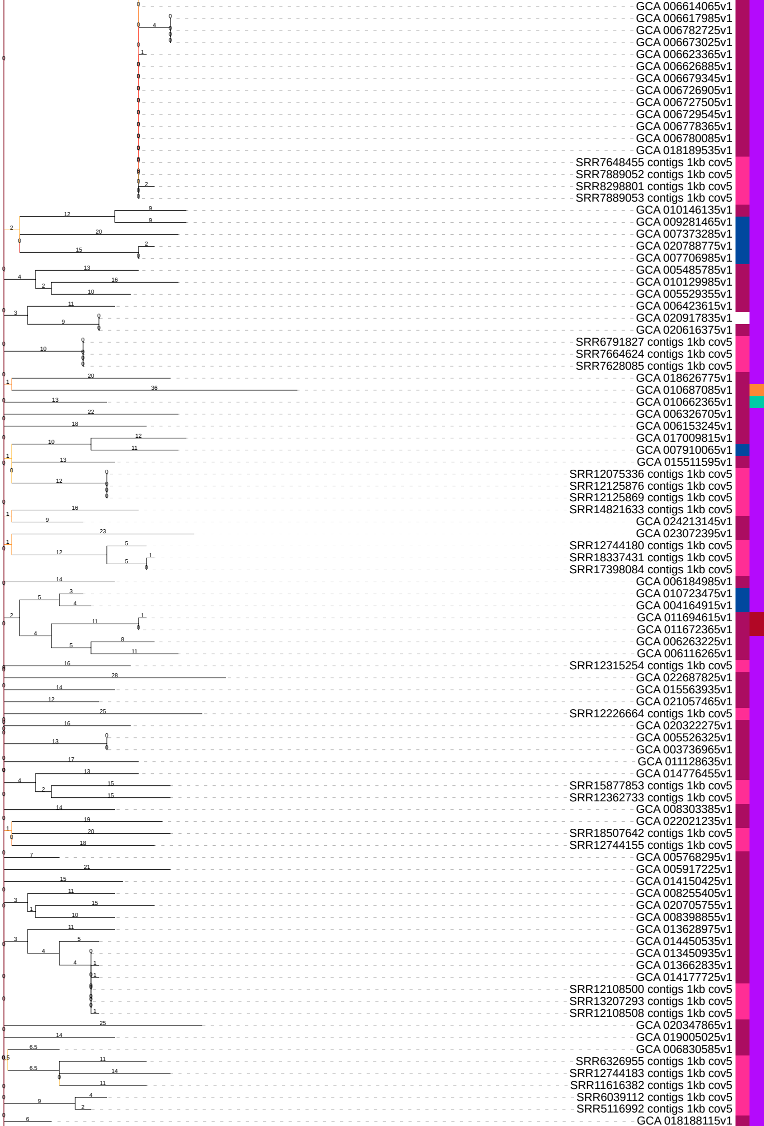
GCA 006614065v1
GCA 006617985v1
GCA 006782725v1
GCA 006673025v1
GCA 006623365v1
GCA 006626885v1
GCA 006679345v1
GCA 006726905v1
GCA 006727505v1
GCA 006729545v1
GCA 006778365v1
GCA 006780085v1
GCA 018189535v1
SRR7648455 contigs 1kb cov5
SRR7889052 contigs 1kb cov5
SRR8298801 contigs 1kb cov5
SRR7889053 contigs 1kb cov5
GCA 010146135v1
GCA 009281465v1
GCA 007373285v1
GCA 020788775v1
GCA 007706985v1
GCA 005485785v1
GCA 010129985v1
GCA 005529355v1
GCA 006423615v1
GCA 020917835v1
GCA 020616375v1
SRR6791827 contigs 1kb cov5
SRR7664624 contigs 1kb cov5
SRR7628085 contigs 1kb cov5
GCA 018626775v1
GCA 010687085v1
GCA 010662365v1
GCA 006326705v1
GCA 006153245v1
GCA 017009815v1
GCA 007910065v1
GCA 015511595v1
SRR12075336 contigs 1kb cov5
SRR12125876 contigs 1kb cov5
SRR12125869 contigs 1kb cov5
SRR14821633 contigs 1kb cov5
GCA 024213145v1
GCA 023072395v1
SRR12744180 contigs 1kb cov5
SRR18337431 contigs 1kb cov5
SRR17398084 contigs 1kb cov5
GCA 006184985v1
GCA 010723475v1
GCA 004164915v1
GCA 011694615v1
GCA 011672365v1
GCA 006263225v1
GCA 006116265v1
SRR12315254 contigs 1kb cov5
GCA 022687825v1
GCA 015563935v1
GCA 021057465v1
SRR12226664 contigs 1kb cov5
GCA 020322275v1
GCA 005526325v1
GCA 003736965v1
GCA 011128635v1
GCA 014776455v1
SRR15877853 contigs 1kb cov5
SRR12362733 contigs 1kb cov5
GCA 008303385v1
GCA 022021235v1
SRR18507642 contigs 1kb cov5
SRR12744155 contigs 1kb cov5
GCA 005768295v1
GCA 005917225v1
GCA 014150425v1
GCA 008255405v1
GCA 020705755v1
GCA 008398855v1
GCA 013628975v1
GCA 014450535v1
GCA 013450935v1
GCA 013662835v1
GCA 014177725v1
SRR12108500 contigs 1kb cov5
SRR13207293 contigs 1kb cov5
SRR12108508 contigs 1kb cov5
GCA 020347865v1
GCA 019005025v1
GCA 006830585v1
SRR6326955 contigs 1kb cov5
SRR12744183 contigs 1kb cov5
SRR11616382 contigs 1kb cov5
SRR6039112 contigs 1kb cov5
SRR5116992 contigs 1kb cov5
GCA 018188115v1

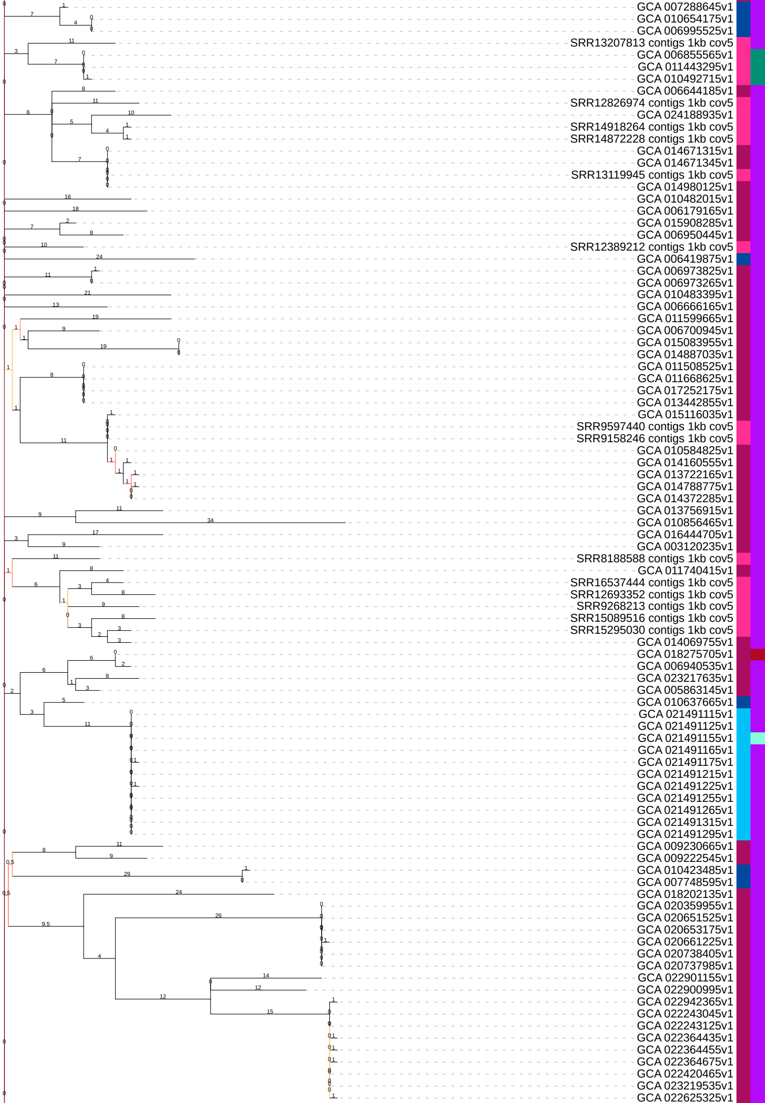
GCA 007288645v1
GCA 010654175v1
GCA 006995525v1
SRR13207813 contigs 1kb cov5
GCA 006855565v1
GCA 011443295v1
GCA 010492715v1
GCA 006644185v1
SRR12826974 contigs 1kb cov5
GCA 024188935v1
SRR14918264 contigs 1kb cov5
SRR14872228 contigs 1kb cov5
GCA 014671315v1
GCA 014671345v1
SRR13119945 contigs 1kb cov5
GCA 014980125v1
GCA 010482015v1
GCA 006179165v1
GCA 015908285v1
GCA 006950445v1
SRR12389212 contigs 1kb cov5
GCA 006419875v1
GCA 006973825v1
GCA 006973265v1
GCA 010483395v1
GCA 006666165v1
GCA 011599665v1
GCA 006700945v1
GCA 015083955v1
GCA 014887035v1
GCA 011508525v1
GCA 011668625v1
GCA 017252175v1
GCA 013442855v1
GCA 015116035v1
SRR9597440 contigs 1kb cov5
SRR9158246 contigs 1kb cov5
GCA 010584825v1
GCA 014160555v1
GCA 013722165v1
GCA 014788775v1
GCA 014372285v1
GCA 013756915v1
GCA 010856465v1
GCA 016444705v1
GCA 003120235v1
SRR8188588 contigs 1kb cov5
GCA 011740415v1
SRR16537444 contigs 1kb cov5
SRR12693352 contigs 1kb cov5
SRR9268213 contigs 1kb cov5
SRR15089516 contigs 1kb cov5
SRR15295030 contigs 1kb cov5
GCA 014069755v1
GCA 018275705v1
GCA 006940535v1
GCA 023217635v1
GCA 005863145v1
GCA 010637665v1
GCA 021491115v1
GCA 021491125v1
GCA 021491155v1
GCA 021491165v1
GCA 021491175v1
GCA 021491215v1
GCA 021491225v1
GCA 021491255v1
GCA 021491265v1
GCA 021491315v1
GCA 021491295v1
GCA 009230665v1
GCA 009222545v1
GCA 010423485v1
GCA 007748595v1
GCA 018202135v1
GCA 020359955v1
GCA 020651525v1
GCA 020653175v1
GCA 020661225v1
GCA 020738405v1
GCA 020737985v1
GCA 022901155v1
GCA 022900995v1
GCA 022942365v1
GCA 022243045v1
GCA 022243125v1
GCA 022364435v1
GCA 022364455v1
GCA 022364675v1
GCA 022420465v1
GCA 023219535v1
GCA 022625325v1

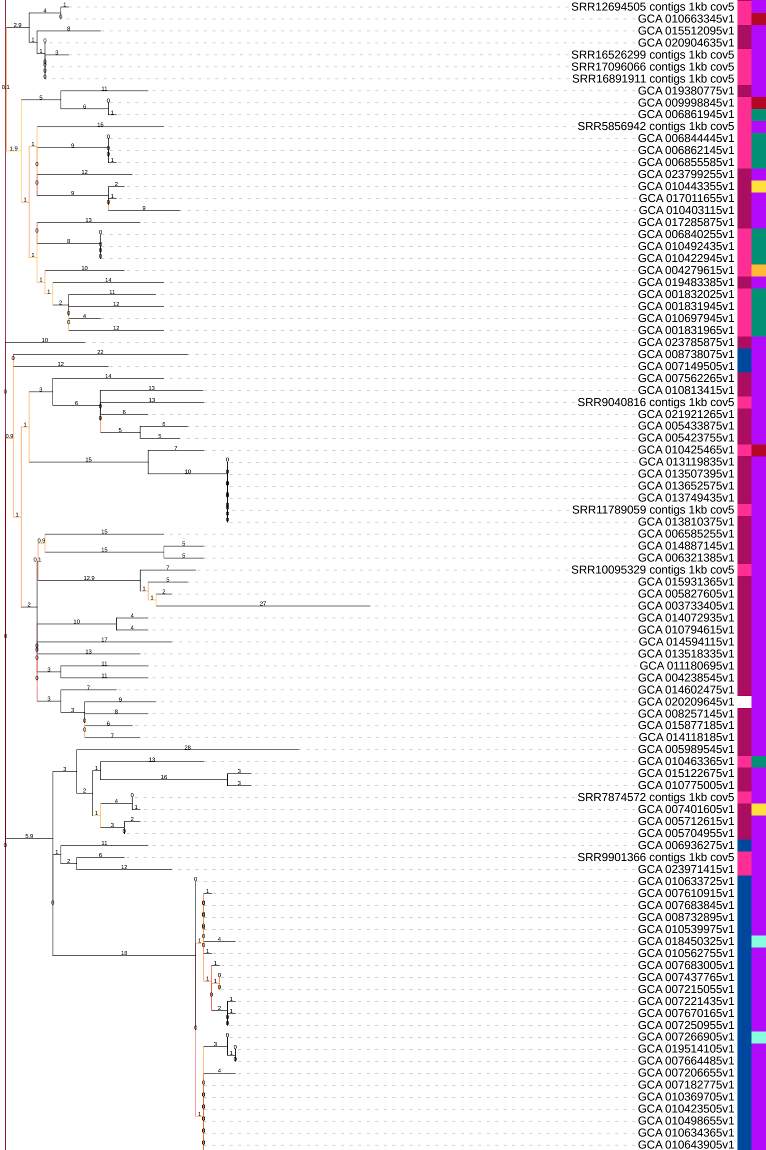

SRR12694505 contigs 1kb cov5
GCA 010663345v1
GCA 015512095v1
GCA 020904635v1
SRR16526299 contigs 1kb cov5
SRR17096066 contigs 1kb cov5
SRR16891911 contigs 1kb cov5
GCA 019380775v1
GCA 009998845v1
GCA 006861945v1
SRR5856942 contigs 1kb cov5
GCA 006844445v1
GCA 006862145v1
GCA 006855585v1
GCA 023799255v1
GCA 010443355v1
GCA 017011655v1
GCA 010403115v1
GCA 017285875v1
GCA 006840255v1
GCA 010492435v1
GCA 010422945v1
GCA 004279615v1
GCA 019483385v1
GCA 001832025v1
GCA 001831945v1
GCA 010697945v1
GCA 001831965v1
GCA 023785875v1
GCA 008738075v1
GCA 007149505v1
GCA 007562265v1
GCA 010813415v1
SRR9040816 contigs 1kb cov5
GCA 021921265v1
GCA 005433875v1
GCA 005423755v1
GCA 010425465v1
GCA 013119835v1
GCA 013507395v1
GCA 013652575v1
GCA 013749435v1
SRR11789059 contigs 1kb cov5
GCA 013810375v1
GCA 006585255v1
GCA 014887145v1
GCA 006321385v1
SRR10095329 contigs 1kb cov5
GCA 015931365v1
GCA 005827605v1
GCA 003733405v1
GCA 014072935v1
GCA 010794615v1
GCA 014594115v1
GCA 013518335v1
GCA 011180695v1
GCA 004238545v1
GCA 014602475v1
GCA 020209645v1
GCA 008257145v1
GCA 015877185v1
GCA 014118185v1
GCA 005989545v1
GCA 010463365v1
GCA 015122675v1
GCA 010775005v1
SRR7874572 contigs 1kb cov5
GCA 007401605v1
GCA 005712615v1
GCA 005704955v1
GCA 006936275v1
SRR9901366 contigs 1kb cov5
GCA 023971415v1
GCA 010633725v1
GCA 007610915v1
GCA 007683845v1
GCA 008732895v1
GCA 010539975v1
GCA 018450325v1
GCA 010562755v1
GCA 007683005v1
GCA 007437765v1
GCA 007215055v1
GCA 007221435v1
GCA 007670165v1
GCA 007250955v1
GCA 007266905v1
GCA 019514105v1
GCA 007664485v1
GCA 007206655v1
GCA 007182775v1
GCA 010369705v1
GCA 010423505v1
GCA 010498655v1
GCA 010634365v1
GCA 010643905v1

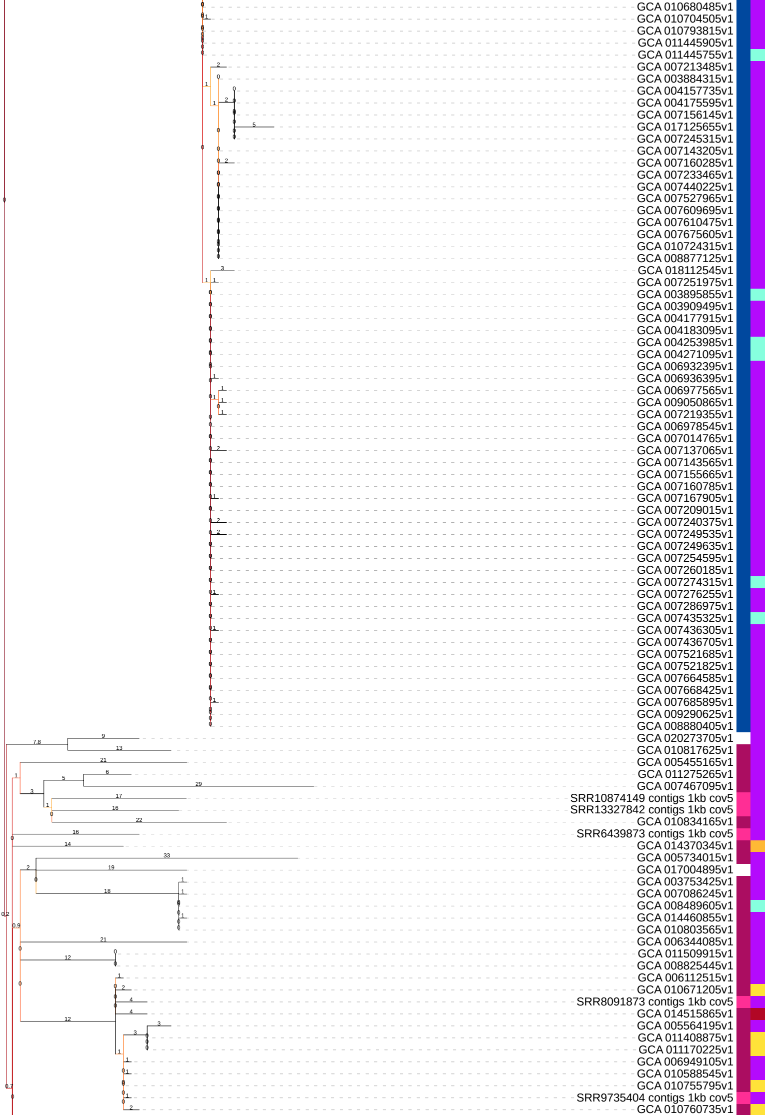
GCA 010680485v1
GCA 010704505v1
GCA 010793815v1
GCA 011445905v1
GCA 011445755v1
GCA 007213485v1
GCA 003884315v1
GCA 004157735v1
GCA 004175595v1
GCA 007156145v1
GCA 017125655v1
GCA 007245315v1
GCA 007143205v1
GCA 007160285v1
GCA 007233465v1
GCA 007440225v1
GCA 007527965v1
GCA 007609695v1
GCA 007610475v1
GCA 007675605v1
GCA 010724315v1
GCA 008877125v1
GCA 018112545v1
GCA 007251975v1
GCA 003895855v1
GCA 003909495v1
GCA 004177915v1
GCA 004183095v1
GCA 004253985v1
GCA 004271095v1
GCA 006932395v1
GCA 006936395v1
GCA 006977565v1
GCA 009050865v1
GCA 007219355v1
GCA 006978545v1
GCA 007014765v1
GCA 007137065v1
GCA 007143565v1
GCA 007155665v1
GCA 007160785v1
GCA 007167905v1
GCA 007209015v1
GCA 007240375v1
GCA 007249535v1
GCA 007249635v1
GCA 007254595v1
GCA 007260185v1
GCA 007274315v1
GCA 007276255v1
GCA 007286975v1
GCA 007435325v1
GCA 007436305v1
GCA 007436705v1
GCA 007521685v1
GCA 007521825v1
GCA 007664585v1
GCA 007668425v1
GCA 007685895v1
GCA 009290625v1
GCA 008880405v1
GCA 020273705v1
GCA 010817625v1
GCA 005455165v1
GCA 011275265v1
GCA 007467095v1
SRR10874149 contigs 1kb cov5
SRR13327842 contigs 1kb cov5
GCA 010834165v1
SRR6439873 contigs 1kb cov5
GCA 014370345v1
GCA 005734015v1
GCA 017004895v1
GCA 003753425v1
GCA 007086245v1
GCA 008489605v1
GCA 014460855v1
GCA 010803565v1
GCA 006344085v1
GCA 011509915v1
GCA 008825445v1
GCA 006112515v1
GCA 010671205v1
SRR8091873 contigs 1kb cov5
GCA 014515865v1
GCA 005564195v1
GCA 011408875v1
GCA 011170225v1
GCA 006949105v1
GCA 010588545v1
GCA 010755795v1
SRR9735404 contigs 1kb cov5
GCA 010760735v1

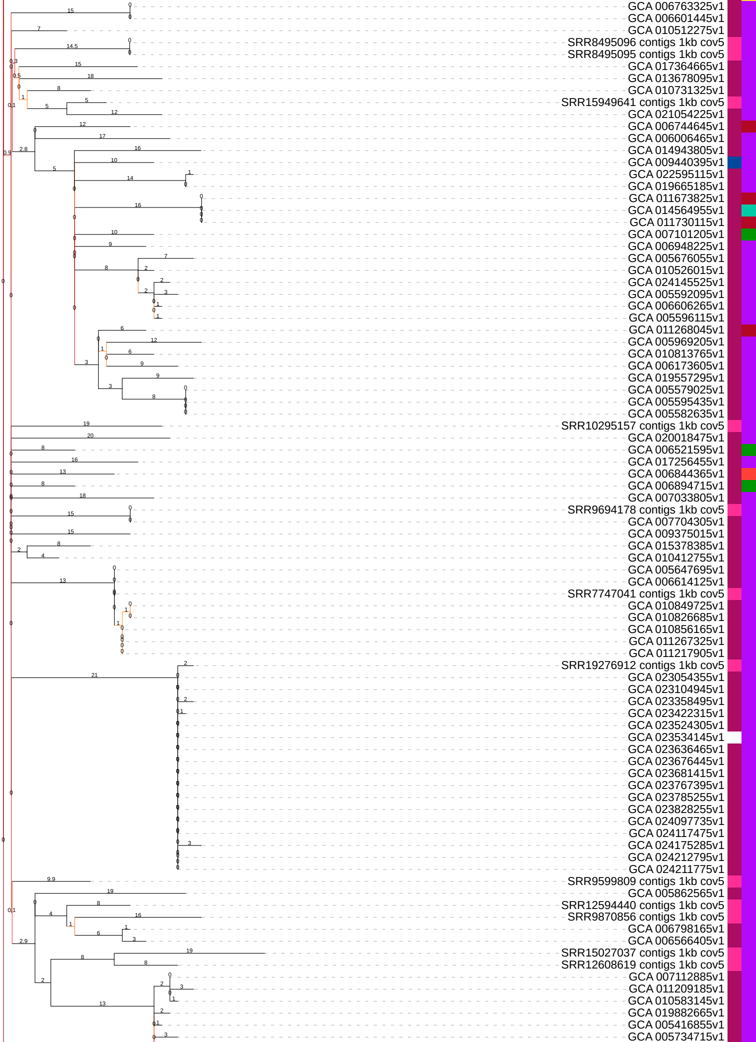
GCA 006763325v1
GCA 006601445v1
GCA 010512275v1
SRR8495096 contigs 1kb cov5
SRR8495095 contigs 1kb cov5
GCA 017364665v1
GCA 013678095v1
GCA 010731325v1
SRR15949641 contigs 1kb cov5
GCA 021054225v1
GCA 006744645v1
GCA 006006465v1
GCA 014943805v1
GCA 009440395v1
GCA 022595115v1
GCA 019665185v1
GCA 011673825v1
GCA 014564955v1
GCA 011730115v1
GCA 007101205v1
GCA 006948225v1
GCA 005676055v1
GCA 010526015v1
GCA 024145525v1
GCA 005592095v1
GCA 006606265v1
GCA 005596115v1
GCA 011268045v1
GCA 005969205v1
GCA 010813765v1
GCA 006173605v1
GCA 019557295v1
GCA 005579025v1
GCA 005595435v1
GCA 005582635v1
SRR10295157 contigs 1kb cov5
GCA 020018475v1
GCA 006521595v1
GCA 017256455v1
GCA 006844365v1
GCA 006894715v1
GCA 007033805v1
SRR9694178 contigs 1kb cov5
GCA 007704305v1
GCA 009375015v1
GCA 015378385v1
GCA 010412755v1
GCA 005647695v1
GCA 006614125v1
SRR7747041 contigs 1kb cov5
GCA 010849725v1
GCA 010826685v1
GCA 010856165v1
GCA 011267325v1
GCA 011217905v1
SRR19276912 contigs 1kb cov5
GCA 023054355v1
GCA 023104945v1
GCA 023358495v1
GCA 023422315v1
GCA 023524305v1
GCA 023534145v1
GCA 023636465v1
GCA 023676445v1
GCA 023681415v1
GCA 023767395v1
GCA 023785255v1
GCA 023828255v1
GCA 024097735v1
GCA 024117475v1
GCA 024175285v1
GCA 024212795v1
GCA 024211775v1
SRR9599809 contigs 1kb cov5
GCA 005862565v1
SRR12594440 contigs 1kb cov5
SRR9870856 contigs 1kb cov5
GCA 006798165v1
GCA 006566405v1
SRR15027037 contigs 1kb cov5
SRR12608619 contigs 1kb cov5
GCA 007112885v1
GCA 011209185v1
GCA 010583145v1
GCA 019882665v1
GCA 005416855v1
GCA 005734715v1

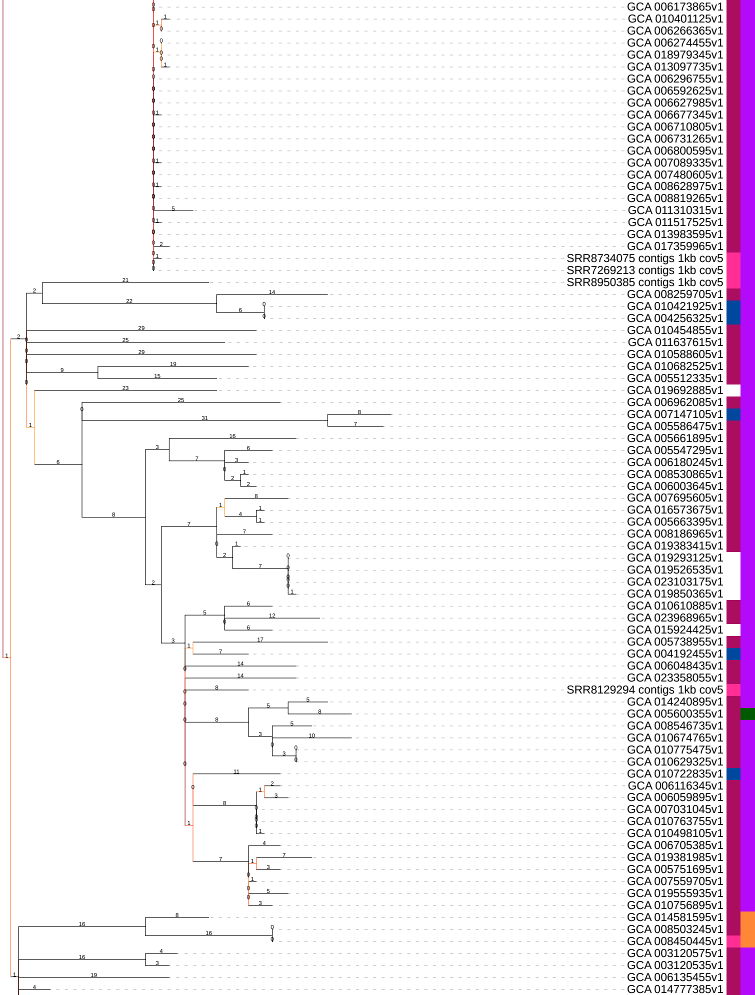
GCA 006173865v1
GCA 010401125v1
GCA 006266365v1
GCA 006274455v1
GCA 018979345v1
GCA 013097735v1
GCA 006296755v1
GCA 006592625v1
GCA 006627985v1
GCA 006677345v1
GCA 006710805v1
GCA 006731265v1
GCA 006800595v1
GCA 007089335v1
GCA 007480605v1
GCA 008628975v1
GCA 008819265v1
GCA 011310315v1
GCA 011517525v1
GCA 013983595v1
GCA 017359965v1
SRR8734075 contigs 1kb cov5
SRR7269213 contigs 1kb cov5
SRR8950385 contigs 1kb cov5
GCA 008259705v1
GCA 010421925v1
GCA 004256325v1
GCA 010454855v1
GCA 011637615v1
GCA 010588605v1
GCA 010682525v1
GCA 005512335v1
GCA 019692885v1
GCA 006962085v1
GCA 007147105v1
GCA 005586475v1
GCA 005661895v1
GCA 005547295v1
GCA 006180245v1
GCA 008530865v1
GCA 006003645v1
GCA 007695605v1
GCA 016573675v1
GCA 005663395v1
GCA 008186965v1
GCA 019383415v1
GCA 019293125v1
GCA 019526535v1
GCA 023103175v1
GCA 019850365v1
GCA 010610885v1
GCA 023968965v1
GCA 015924425v1
GCA 005738955v1
GCA 004192455v1
GCA 006048435v1
GCA 023358055v1
SRR8129294 contigs 1kb cov5
GCA 014240895v1
GCA 005600355v1
GCA 008546735v1
GCA 010674765v1
GCA 010775475v1
GCA 010629325v1
GCA 010722835v1
GCA 006116345v1
GCA 006059895v1
GCA 007031045v1
GCA 010763755v1
GCA 010498105v1
GCA 006705385v1
GCA 019381985v1
GCA 005751695v1
GCA 007559705v1
GCA 019555935v1
GCA 010756895v1
GCA 014581595v1
GCA 008503245v1
GCA 008450445v1
GCA 003120575v1
GCA 003120535v1
GCA 006135455v1
GCA 014777385v1

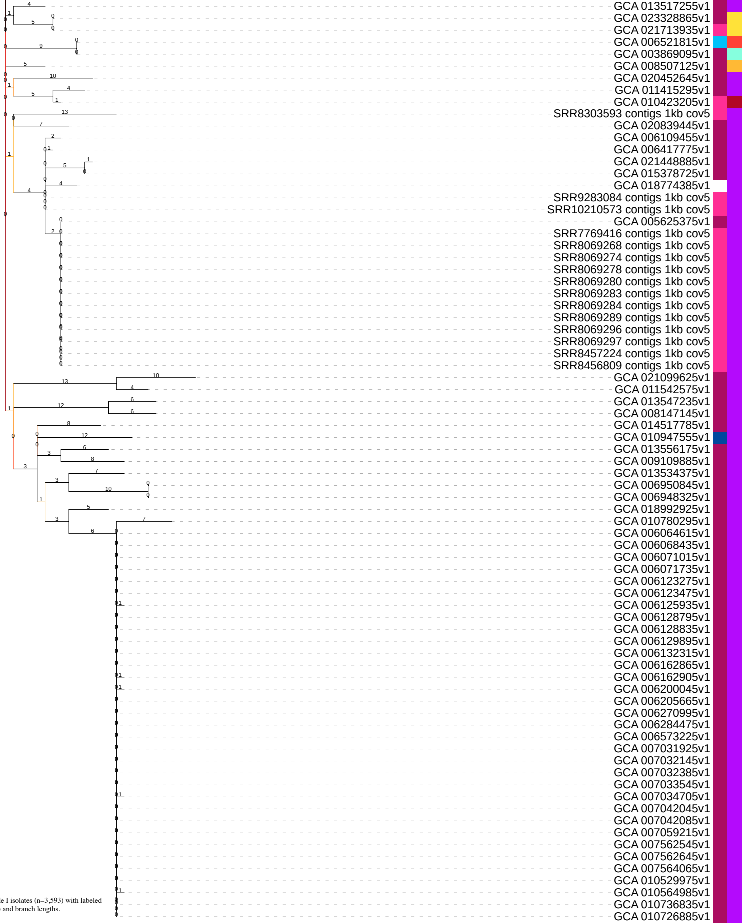

Fig. S1. Phylogenetic tree of Clade I isolates (n=3,593) with labeled isolate IDs, bootstrap values (0-1) and branch lengths.
