# Supplementary material for: Salmonella enterica serovar Braenderup shows clade-specific source associations and a high proportion of molecular epidemiological clustering
Source: Appl Environ Microbiol. 2025 Mar 21;91(4):e02594-24. doi: 10.1128/aem.02594-24 (PMC12016519; doi:10.1128/aem.02594-24)
Supplement: Fig. S2 — Phylogenetic tree of Clade II isolates with labeled isolate IDs. [file aem.02594-24-s0002.pdf]

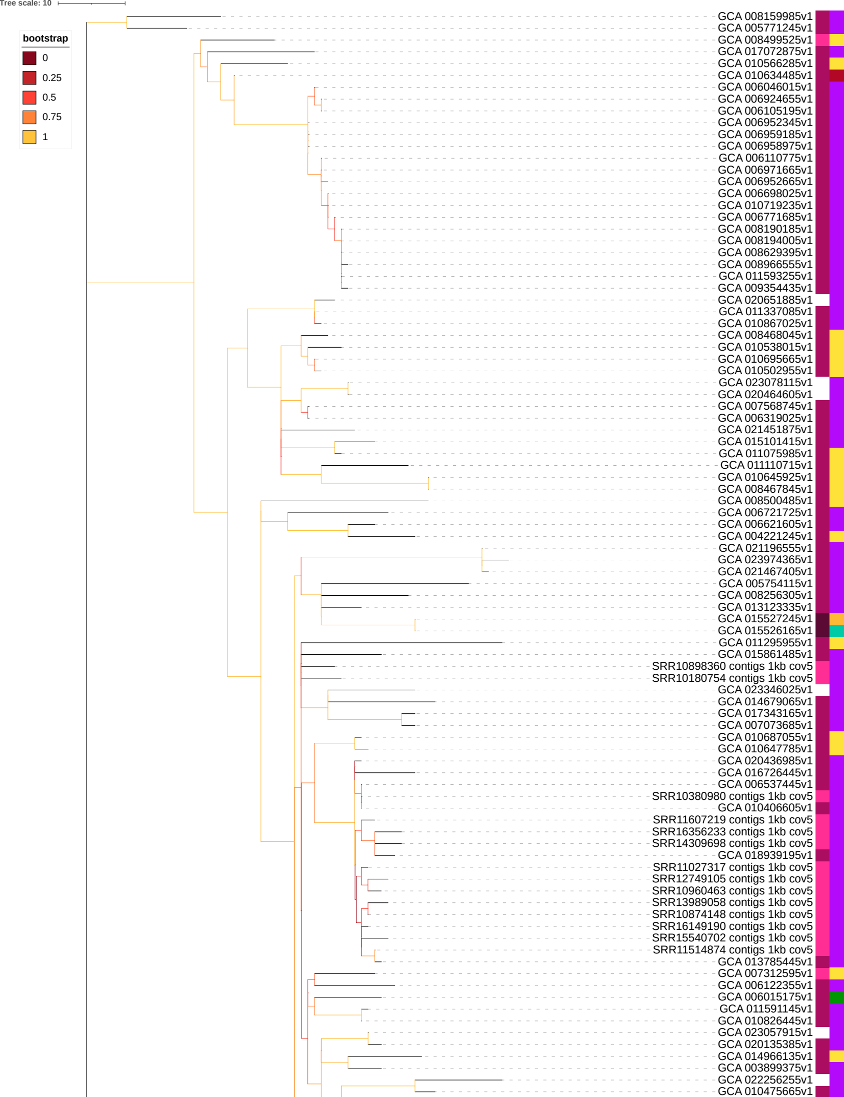

Tree scale: 10
bootstrap
0
0.25
0.5
0.75
1
GCA 008159985v1
GCA 005771245v1
GCA 008499525v1
GCA 017072875v1
GCA 010566285v1
GCA 010634485v1
GCA 006046015v1
GCA 006924655v1
GCA 006105195v1
GCA 006952345v1
GCA 006959185v1
GCA 006958975v1
GCA 006110775v1
GCA 006971665v1
GCA 006952665v1
GCA 006698025v1
GCA 010719235v1
GCA 006771685v1
GCA 008190185v1
GCA 008194005v1
GCA 008629395v1
GCA 008966555v1
GCA 011593255v1
GCA 009354435v1
GCA 020651885v1
GCA 011337085v1
GCA 010867025v1
GCA 008468045v1
GCA 010538015v1
GCA 010695665v1
GCA 010502955v1
GCA 023078115v1
GCA 020464605v1
GCA 007568745v1
GCA 006319025v1
GCA 021451875v1
GCA 015101415v1
GCA 011075985v1
GCA 011110715v1
GCA 010645925v1
GCA 008467845v1
GCA 008500485v1
GCA 006721725v1
GCA 006621605v1
GCA 004221245v1
GCA 021196555v1
GCA 023974365v1
GCA 021467405v1
GCA 005754115v1
GCA 008256305v1
GCA 013123335v1
GCA 015527245v1
GCA 015526165v1
GCA 011295955v1
GCA 015861485v1
SRR10898360 contigs 1kb cov5
SRR10180754 contigs 1kb cov5
GCA 023346025v1
GCA 014679065v1
GCA 017343165v1
GCA 007073685v1
GCA 010687055v1
GCA 010647785v1
GCA 020436985v1
GCA 016726445v1
GCA 006537445v1
SRR10380980 contigs 1kb cov5
GCA 010406605v1
SRR11607219 contigs 1kb cov5
SRR16356233 contigs 1kb cov5
SRR14309698 contigs 1kb cov5
GCA 018939195v1
SRR11027317 contigs 1kb cov5
SRR12749105 contigs 1kb cov5
SRR10960463 contigs 1kb cov5
SRR13989058 contigs 1kb cov5
SRR10874148 contigs 1kb cov5
SRR16149190 contigs 1kb cov5
SRR15540702 contigs 1kb cov5
SRR11514874 contigs 1kb cov5
GCA 013785445v1
GCA 007312595v1
GCA 006122355v1
GCA 006015175v1
GCA 011591145v1
GCA 010826445v1
GCA 023057915v1
GCA 020135385v1
GCA 014966135v1
GCA 003899375v1
GCA 022256255v1
GCA 010475665v1

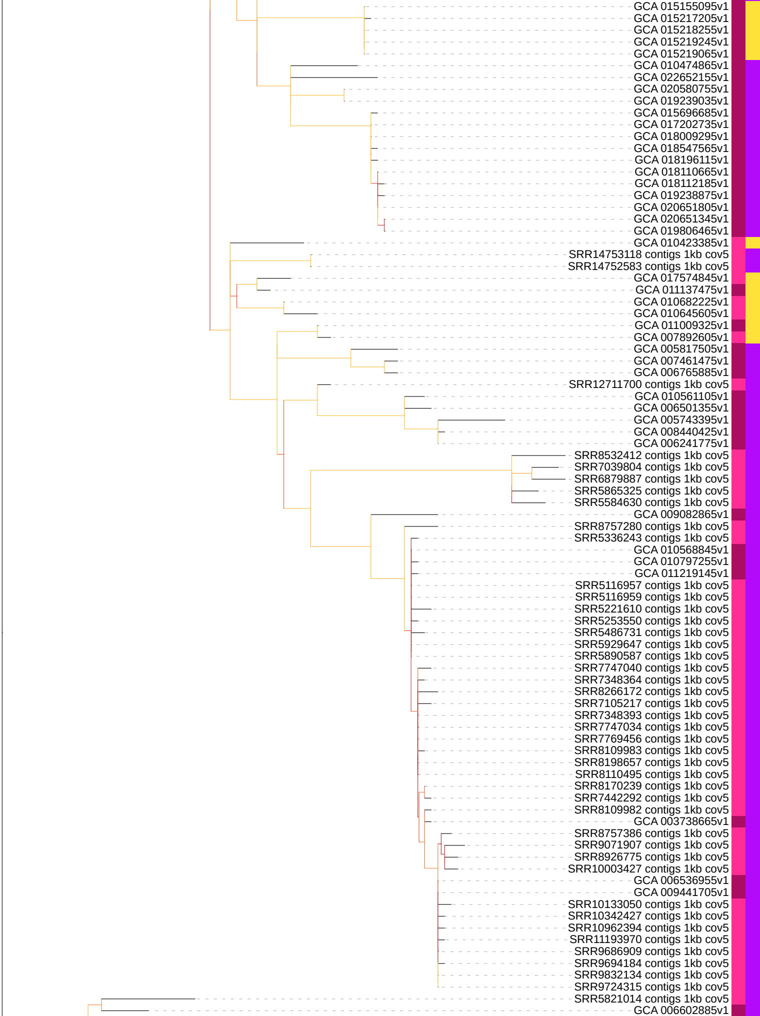
GCA 015155095v1
GCA 015217205v1
GCA 015218255v1
GCA 015219245v1
GCA 015219065v1
GCA 010474865v1
GCA 022652155v1
GCA 020580755v1
GCA 019239035v1
GCA 015696685v1
GCA 017202735v1
GCA 018009295v1
GCA 018547565v1
GCA 018196115v1
GCA 018110665v1
GCA 018112185v1
GCA 019238875v1
GCA 020651805v1
GCA 020651345v1
GCA 019806465v1
GCA 010423385v1
SRR14753118 contigs 1kb cov5
SRR14752583 contigs 1kb cov5
GCA 017574845v1
GCA 011137475v1
GCA 010682225v1
GCA 010645605v1
GCA 011009325v1
GCA 007892605v1
GCA 005817505v1
GCA 007461475v1
GCA 006765885v1
SRR12711700 contigs 1kb cov5
GCA 010561105v1
GCA 006501355v1
GCA 005743395v1
GCA 008440425v1
GCA 006241775v1
SRR8532412 contigs 1kb cov5
SRR7039804 contigs 1kb cov5
SRR6879887 contigs 1kb cov5
SRR5865325 contigs 1kb cov5
SRR5584630 contigs 1kb cov5
GCA 009082865v1
SRR8757280 contigs 1kb cov5
SRR5336243 contigs 1kb cov5
GCA 010568845v1
GCA 010797255v1
GCA 011219145v1
SRR5116957 contigs 1kb cov5
SRR5116959 contigs 1kb cov5
SRR5221610 contigs 1kb cov5
SRR5253550 contigs 1kb cov5
SRR5486731 contigs 1kb cov5
SRR5929647 contigs 1kb cov5
SRR5890587 contigs 1kb cov5
SRR7747040 contigs 1kb cov5
SRR7348364 contigs 1kb cov5
SRR8266172 contigs 1kb cov5
SRR7105217 contigs 1kb cov5
SRR7348393 contigs 1kb cov5
SRR7747034 contigs 1kb cov5
SRR7769456 contigs 1kb cov5
SRR8109983 contigs 1kb cov5
SRR8198657 contigs 1kb cov5
SRR8110495 contigs 1kb cov5
SRR8170239 contigs 1kb cov5
SRR7442292 contigs 1kb cov5
SRR8109982 contigs 1kb cov5
GCA 003738665v1
SRR8757386 contigs 1kb cov5
SRR9071907 contigs 1kb cov5
SRR8926775 contigs 1kb cov5
SRR10003427 contigs 1kb cov5
GCA 006536955v1
GCA 009441705v1
SRR10133050 contigs 1kb cov5
SRR10342427 contigs 1kb cov5
SRR10962394 contigs 1kb cov5
SRR11193970 contigs 1kb cov5
SRR9686909 contigs 1kb cov5
SRR9694184 contigs 1kb cov5
SRR9832134 contigs 1kb cov5
SRR9724315 contigs 1kb cov5
SRR5821014 contigs 1kb cov5
GCA 006602885v1

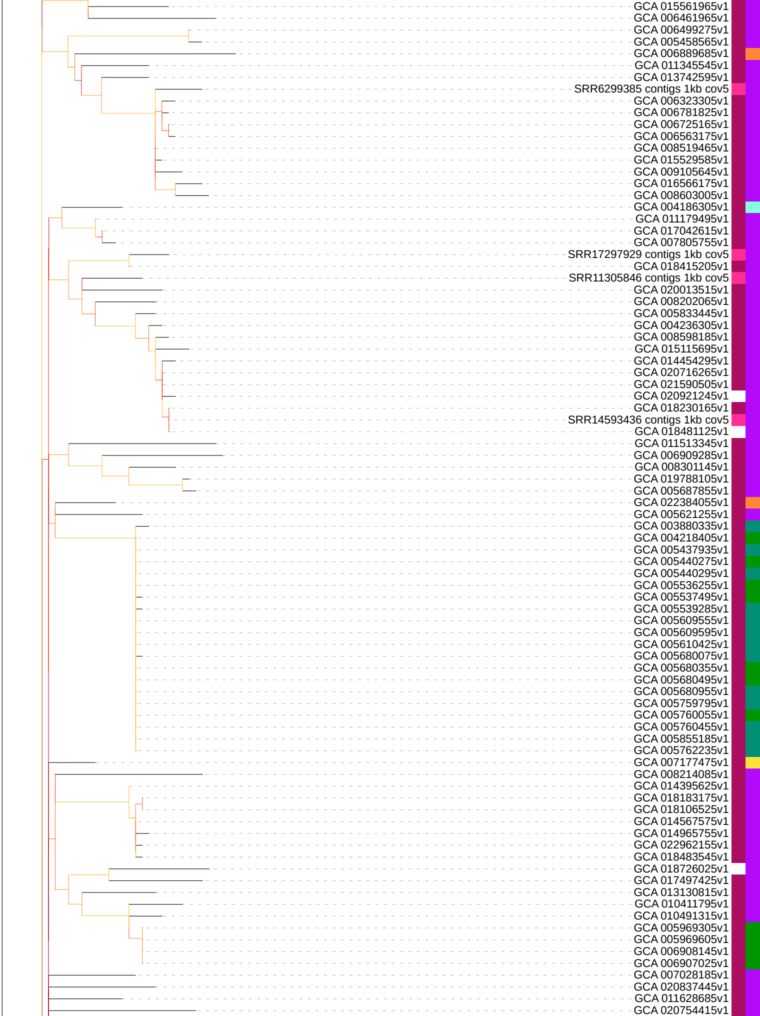
GCA 015561965v1
GCA 006461965v1
GCA 006499275v1
GCA 005458565v1
GCA 006889685v1
GCA 011345545v1
GCA 013742595v1
SRR6299385 contigs 1kb cov5
GCA 006323305v1
GCA 006781825v1
GCA 006725165v1
GCA 006563175v1
GCA 008519465v1
GCA 015529585v1
GCA 009105645v1
GCA 016566175v1
GCA 008603005v1
GCA 004186305v1
GCA 011179495v1
GCA 017042615v1
GCA 007805755v1
SRR17297929 contigs 1kb cov5
GCA 018415205v1
SRR11305846 contigs 1kb cov5
GCA 020013515v1
GCA 008202065v1
GCA 005833445v1
GCA 004236305v1
GCA 008598185v1
GCA 015115695v1
GCA 014454295v1
GCA 020716265v1
GCA 021590505v1
GCA 020921245v1
GCA 018230165v1
SRR14593436 contigs 1kb cov5
GCA 018481125v1
GCA 011513345v1
GCA 006909285v1
GCA 008301145v1
GCA 019788105v1
GCA 005687855v1
GCA 022384055v1
GCA 005621255v1
GCA 003880335v1
GCA 004218405v1
GCA 005437935v1
GCA 005440275v1
GCA 005440295v1
GCA 005536255v1
GCA 005537495v1
GCA 005539285v1
GCA 005609555v1
GCA 005609595v1
GCA 005610425v1
GCA 005680075v1
GCA 005680355v1
GCA 005680495v1
GCA 005680955v1
GCA 005759795v1
GCA 005760055v1
GCA 005760455v1
GCA 005855185v1
GCA 005762235v1
GCA 007177475v1
GCA 008214085v1
GCA 014395625v1
GCA 018183175v1
GCA 018106525v1
GCA 014567575v1
GCA 014965755v1
GCA 022962155v1
GCA 018483545v1
GCA 018726025v1
GCA 017497425v1
GCA 013130815v1
GCA 010411795v1
GCA 010491315v1
GCA 005969305v1
GCA 005969605v1
GCA 006908145v1
GCA 006907025v1
GCA 007028185v1
GCA 020837445v1
GCA 011628685v1
GCA 020754415v1

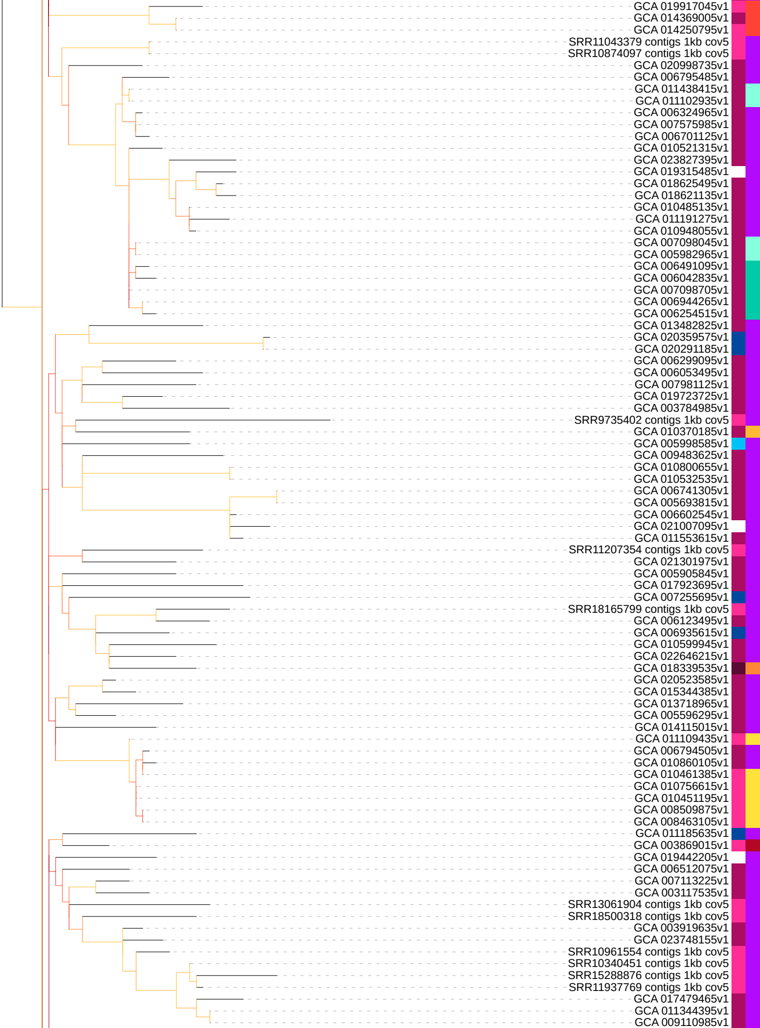
GCA 019917045v1
GCA 014369005v1
GCA 014250795v1
SRR11043379 contigs 1kb cov5
SRR10874097 contigs 1kb cov5
GCA 020998735v1
GCA 006795485v1
GCA 011438415v1
GCA 011102935v1
GCA 006324965v1
GCA 007575985v1
GCA 006701125v1
GCA 010521315v1
GCA 023827395v1
GCA 019315485v1
GCA 018625495v1
GCA 018621135v1
GCA 010485135v1
GCA 011191275v1
GCA 010948055v1
GCA 007098045v1
GCA 005982965v1
GCA 006491095v1
GCA 006042835v1
GCA 007098705v1
GCA 006944265v1
GCA 006254515v1
GCA 013482825v1
GCA 020359575v1
GCA 020291185v1
GCA 006299095v1
GCA 006053495v1
GCA 007981125v1
GCA 019723725v1
GCA 003784985v1
SRR9735402 contigs 1kb cov5
GCA 010370185v1
GCA 005998585v1
GCA 009483625v1
GCA 010800655v1
GCA 010532535v1
GCA 006741305v1
GCA 005693815v1
GCA 006602545v1
GCA 021007095v1
GCA 011553615v1
SRR11207354 contigs 1kb cov5
GCA 021301975v1
GCA 005905845v1
GCA 017923695v1
GCA 007255695v1
SRR18165799 contigs 1kb cov5
GCA 006123495v1
GCA 006935615v1
GCA 010599945v1
GCA 022646215v1
GCA 018339535v1
GCA 020523585v1
GCA 015344385v1
GCA 013718965v1
GCA 005596295v1
GCA 014115015v1
GCA 011109435v1
GCA 006794505v1
GCA 010860105v1
GCA 010461385v1
GCA 010756615v1
GCA 010451195v1
GCA 008509875v1
GCA 008463105v1
GCA 011185635v1
GCA 003869015v1
GCA 019442205v1
GCA 006512075v1
GCA 007113225v1
GCA 003117535v1
SRR13061904 contigs 1kb cov5
SRR18500318 contigs 1kb cov5
GCA 003919635v1
GCA 023748155v1
SRR10961554 contigs 1kb cov5
SRR10340451 contigs 1kb cov5
SRR15288876 contigs 1kb cov5
SRR11937769 contigs 1kb cov5
GCA 017479465v1
GCA 011344395v1
GCA 009110985v1

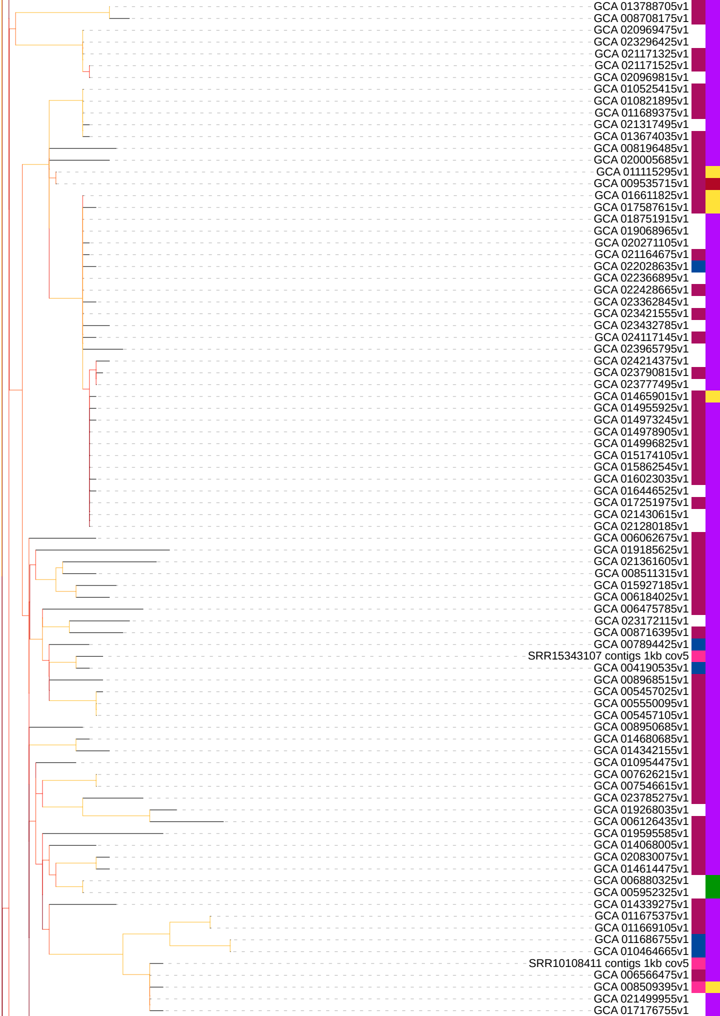
GCA 013788705v1
GCA 008708175v1
GCA 020969475v1
GCA 023296425v1
GCA 021171325v1
GCA 021171525v1
GCA 020969815v1
GCA 010525415v1
GCA 010821895v1
GCA 011689375v1
GCA 021317495v1
GCA 013674035v1
GCA 008196485v1
GCA 020005685v1
GCA 011115295v1
GCA 009535715v1
GCA 016611825v1
GCA 017587615v1
GCA 018751915v1
GCA 019068965v1
GCA 020271105v1
GCA 021164675v1
GCA 022028635v1
GCA 022366895v1
GCA 022428665v1
GCA 023362845v1
GCA 023421555v1
GCA 023432785v1
GCA 024117145v1
GCA 023965795v1
GCA 024214375v1
GCA 023790815v1
GCA 023777495v1
GCA 014659015v1
GCA 014955925v1
GCA 014973245v1
GCA 014978905v1
GCA 014996825v1
GCA 015174105v1
GCA 015862545v1
GCA 016023035v1
GCA 016446525v1
GCA 017251975v1
GCA 021430615v1
GCA 021280185v1
GCA 006062675v1
GCA 019185625v1
GCA 021361605v1
GCA 008511315v1
GCA 015927185v1
GCA 006184025v1
GCA 006475785v1
GCA 023172115v1
GCA 008716395v1
GCA 007894425v1
SRR15343107 contigs 1kb cov5
GCA 004190535v1
GCA 008968515v1
GCA 005457025v1
GCA 005550095v1
GCA 005457105v1
GCA 008950685v1
GCA 014680685v1
GCA 014342155v1
GCA 010954475v1
GCA 007626215v1
GCA 007546615v1
GCA 023785275v1
GCA 019268035v1
GCA 006126435v1
GCA 019595585v1
GCA 014068005v1
GCA 020830075v1
GCA 014614475v1
GCA 006880325v1
GCA 005952325v1
GCA 014339275v1
GCA 011675375v1
GCA 011669105v1
GCA 011686755v1
GCA 010464665v1
SRR10108411 contigs 1kb cov5
GCA 006566475v1
GCA 008509395v1
GCA 021499955v1
GCA 017176755v1

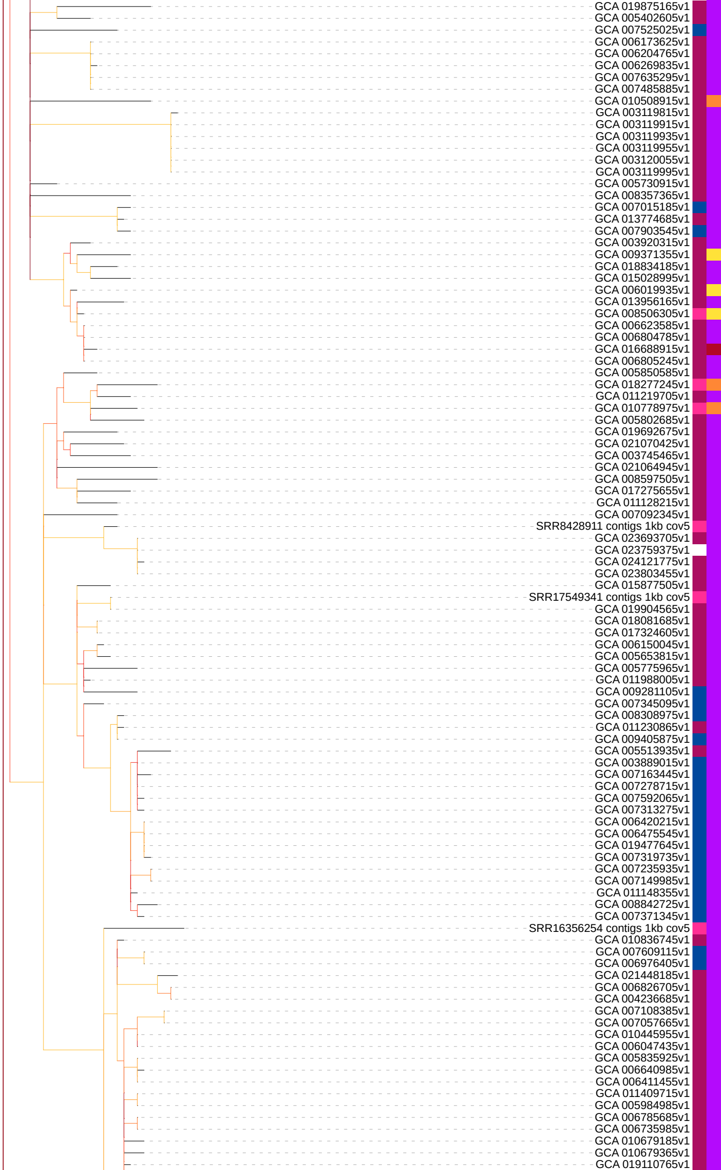

GCA 019875165v1
GCA 005402605v1
GCA 007525025v1
GCA 006173625v1
GCA 006204765v1
GCA 006269835v1
GCA 007635295v1
GCA 007485885v1
GCA 010508915v1
GCA 003119815v1
GCA 003119915v1
GCA 003119935v1
GCA 003119955v1
GCA 003120055v1
GCA 003119995v1
GCA 005730915v1
GCA 008357365v1
GCA 007015185v1
GCA 013774685v1
GCA 007903545v1
GCA 003920315v1
GCA 009371355v1
GCA 018834185v1
GCA 015028995v1
GCA 006019935v1
GCA 013956165v1
GCA 008506305v1
GCA 006623585v1
GCA 006804785v1
GCA 016688915v1
GCA 006805245v1
GCA 005850585v1
GCA 018277245v1
GCA 011219705v1
GCA 010778975v1
GCA 005802685v1
GCA 019692675v1
GCA 021070425v1
GCA 003745465v1
GCA 021064945v1
GCA 008597505v1
GCA 017275655v1
GCA 011128215v1
GCA 007092345v1
SRR8428911 contigs 1kb cov5
GCA 023693705v1
GCA 023759375v1
GCA 024121775v1
GCA 023803455v1
GCA 015877505v1
SRR17549341 contigs 1kb cov5
GCA 019904565v1
GCA 018081685v1
GCA 017324605v1
GCA 006150045v1
GCA 005653815v1
GCA 005775965v1
GCA 011988005v1
GCA 009281105v1
GCA 007345095v1
GCA 008308975v1
GCA 011230865v1
GCA 009405875v1
GCA 005513935v1
GCA 003889015v1
GCA 007163445v1
GCA 007278715v1
GCA 007592065v1
GCA 007313275v1
GCA 006420215v1
GCA 006475545v1
GCA 019477645v1
GCA 007319735v1
GCA 007235935v1
GCA 007149985v1
GCA 011148355v1
GCA 008842725v1
GCA 007371345v1
SRR16356254 contigs 1kb cov5
GCA 010836745v1
GCA 007609115v1
GCA 006976405v1
GCA 021448185v1
GCA 006826705v1
GCA 004236685v1
GCA 007108385v1
GCA 007057665v1
GCA 010445955v1
GCA 006047435v1
GCA 005835925v1
GCA 006640985v1
GCA 006411455v1
GCA 011409715v1
GCA 005984985v1
GCA 006785685v1
GCA 006735985v1
GCA 010679185v1
GCA 010679365v1
GCA 019110765v1

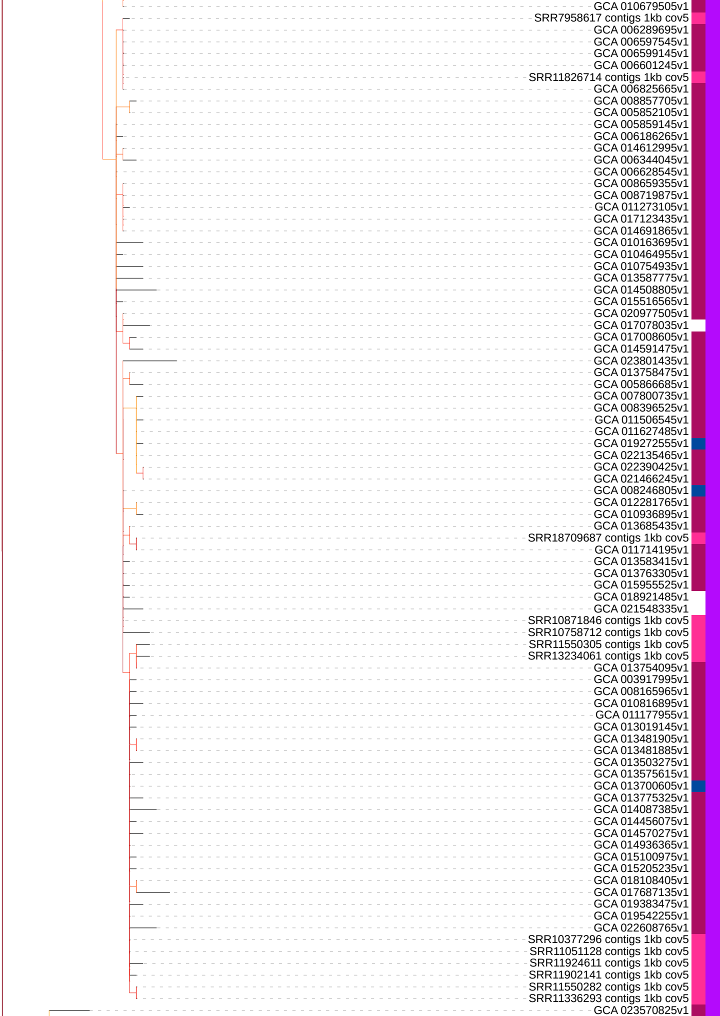
GCA 010679505v1
SRR7958617 contigs 1kb cov5
GCA 006289695v1
GCA 006597545v1
GCA 006599145v1
GCA 006601245v1
SRR11826714 contigs 1kb cov5
GCA 006825665v1
GCA 008857705v1
GCA 005852105v1
GCA 005859145v1
GCA 006186265v1
GCA 014612995v1
GCA 006344045v1
GCA 006628545v1
GCA 008659355v1
GCA 008719875v1
GCA 011273105v1
GCA 017123435v1
GCA 014691865v1
GCA 010163695v1
GCA 010464955v1
GCA 010754935v1
GCA 013587775v1
GCA 014508805v1
GCA 015516565v1
GCA 020977505v1
GCA 017078035v1
GCA 017008605v1
GCA 014591475v1
GCA 023801435v1
GCA 013758475v1
GCA 005866685v1
GCA 007800735v1
GCA 008396525v1
GCA 011506545v1
GCA 011627485v1
GCA 019272555v1
GCA 022135465v1
GCA 022390425v1
GCA 021466245v1
GCA 008246805v1
GCA 012281765v1
GCA 010936895v1
GCA 013685435v1
SRR18709687 contigs 1kb cov5
GCA 011714195v1
GCA 013583415v1
GCA 013763305v1
GCA 015955525v1
GCA 018921485v1
GCA 021548335v1
SRR10871846 contigs 1kb cov5
SRR10758712 contigs 1kb cov5
SRR11550305 contigs 1kb cov5
SRR13234061 contigs 1kb cov5
GCA 013754095v1
GCA 003917995v1
GCA 008165965v1
GCA 010816895v1
GCA 011177955v1
GCA 013019145v1
GCA 013481905v1
GCA 013481885v1
GCA 013503275v1
GCA 013575615v1
GCA 013700605v1
GCA 013775325v1
GCA 014087385v1
GCA 014456075v1
GCA 014570275v1
GCA 014936365v1
GCA 015100975v1
GCA 015205235v1
GCA 018108405v1
GCA 017687135v1
GCA 019383475v1
GCA 019542255v1
GCA 022608765v1
SRR10377296 contigs 1kb cov5
SRR11051128 contigs 1kb cov5
SRR11924611 contigs 1kb cov5
SRR11902141 contigs 1kb cov5
SRR11550282 contigs 1kb cov5
SRR11336293 contigs 1kb cov5
GCA 023570825v1

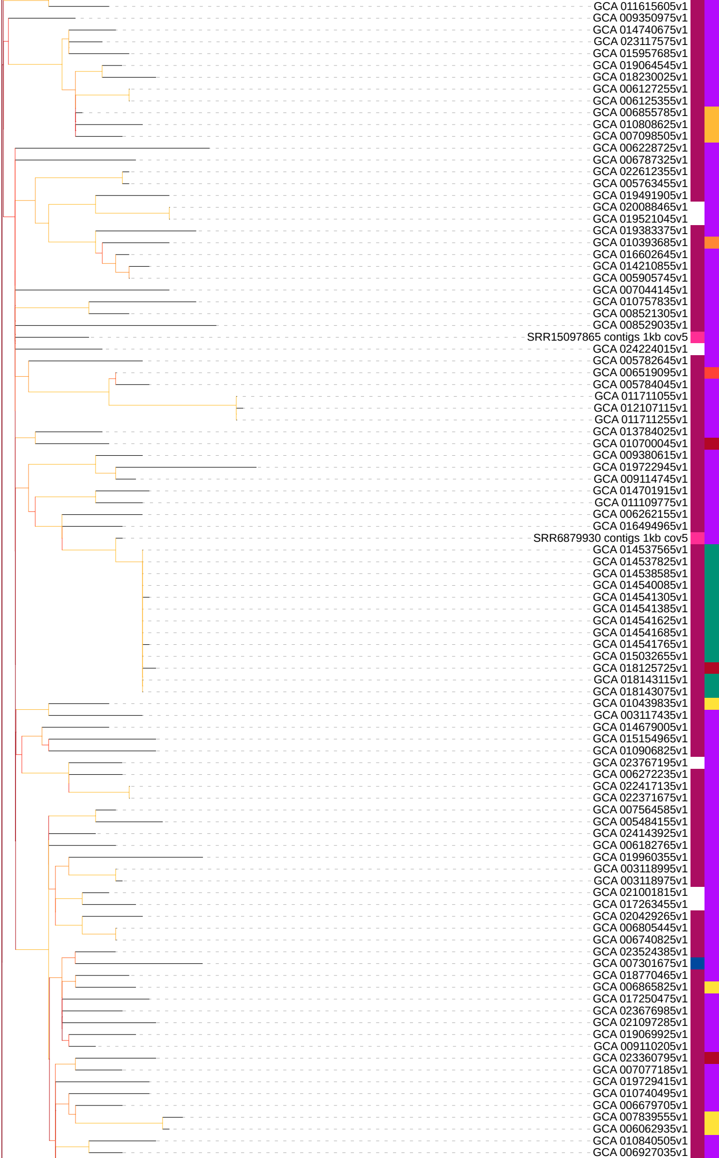
GCA 011615605v1
GCA 009350975v1
GCA 014740675v1
GCA 023117575v1
GCA 015957685v1
GCA 019064545v1
GCA 018230025v1
GCA 006127255v1
GCA 006125355v1
GCA 006855785v1
GCA 010808625v1
GCA 007098505v1
GCA 006228725v1
GCA 006787325v1
GCA 022612355v1
GCA 005763455v1
GCA 019491905v1
GCA 020088465v1
GCA 019521045v1
GCA 019383375v1
GCA 010393685v1
GCA 016602645v1
GCA 014210855v1
GCA 005905745v1
GCA 007044145v1
GCA 010757835v1
GCA 008521305v1
GCA 008529035v1
SRR15097865 contigs 1kb cov5
GCA 024224015v1
GCA 005782645v1
GCA 006519095v1
GCA 005784045v1
GCA 011711055v1
GCA 012107115v1
GCA 011711255v1
GCA 013784025v1
GCA 010700045v1
GCA 009380615v1
GCA 019722945v1
GCA 009114745v1
GCA 014701915v1
GCA 011109775v1
GCA 006262155v1
GCA 016494965v1
SRR6879930 contigs 1kb cov5
GCA 014537565v1
GCA 014537825v1
GCA 014538585v1
GCA 014540085v1
GCA 014541305v1
GCA 014541385v1
GCA 014541625v1
GCA 014541685v1
GCA 014541765v1
GCA 015032655v1
GCA 018125725v1
GCA 018143115v1
GCA 018143075v1
GCA 010439835v1
GCA 003117435v1
GCA 014679005v1
GCA 015154965v1
GCA 010906825v1
GCA 023767195v1
GCA 006272235v1
GCA 022417135v1
GCA 022371675v1
GCA 007564585v1
GCA 005484155v1
GCA 024143925v1
GCA 006182765v1
GCA 019960355v1
GCA 003118995v1
GCA 003118975v1
GCA 021001815v1
GCA 017263455v1
GCA 020429265v1
GCA 006805445v1
GCA 006740825v1
GCA 023524385v1
GCA 007301675v1
GCA 018770465v1
GCA 006865825v1
GCA 017250475v1
GCA 023676985v1
GCA 021097285v1
GCA 019069925v1
GCA 009110205v1
GCA 023360795v1
GCA 007077185v1
GCA 019729415v1
GCA 010740495v1
GCA 006679705v1
GCA 007839555v1
GCA 006062935v1
GCA 010840505v1
GCA 006927035v1

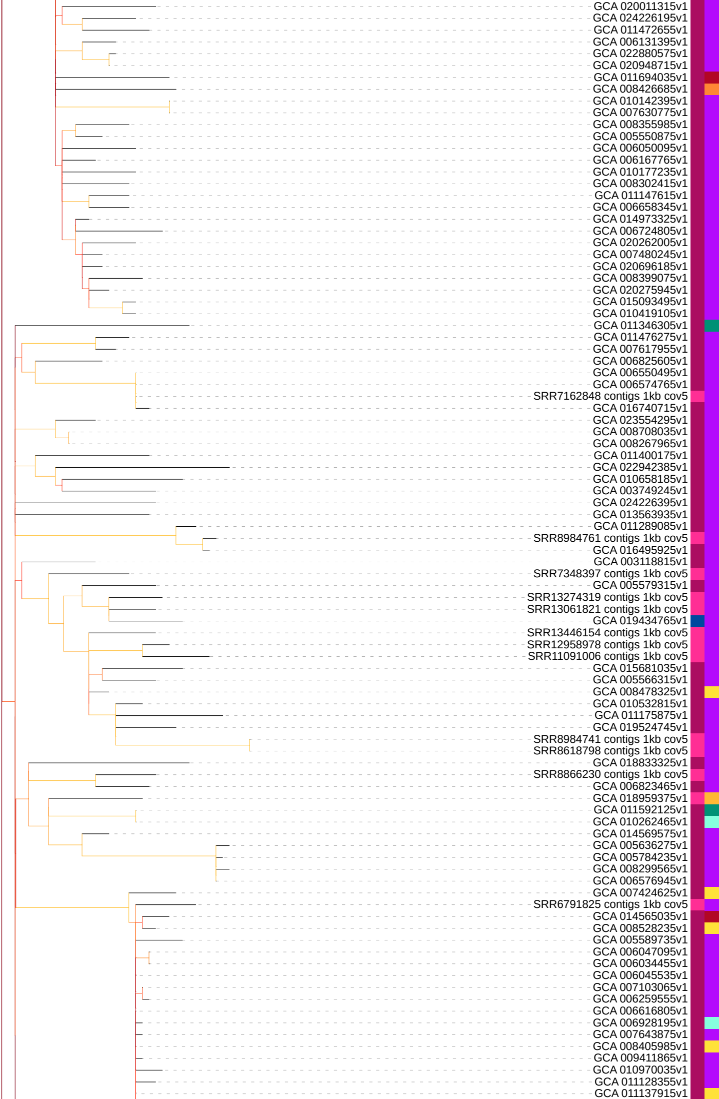
GCA 020011315v1
GCA 024226195v1
GCA 011472655v1
GCA 006131395v1
GCA 022880575v1
GCA 020948715v1
GCA 011694035v1
GCA 008426685v1
GCA 010142395v1
GCA 007630775v1
GCA 008355985v1
GCA 005550875v1
GCA 006050095v1
GCA 006167765v1
GCA 010177235v1
GCA 008302415v1
GCA 011147615v1
GCA 006658345v1
GCA 014973325v1
GCA 006724805v1
GCA 020262005v1
GCA 007480245v1
GCA 020696185v1
GCA 008399075v1
GCA 020275945v1
GCA 015093495v1
GCA 010419105v1
GCA 011346305v1
GCA 011476275v1
GCA 007617955v1
GCA 006825605v1
GCA 006550495v1
GCA 006574765v1
SRR7162848 contigs 1kb cov5
GCA 016740715v1
GCA 023554295v1
GCA 008708035v1
GCA 008267965v1
GCA 011400175v1
GCA 022942385v1
GCA 010658185v1
GCA 003749245v1
GCA 024226395v1
GCA 013563935v1
GCA 011289085v1
SRR8984761 contigs 1kb cov5
GCA 016495925v1
GCA 003118815v1
SRR7348397 contigs 1kb cov5
GCA 005579315v1
SRR13274319 contigs 1kb cov5
SRR13061821 contigs 1kb cov5
GCA 019434765v1
SRR13446154 contigs 1kb cov5
SRR12958978 contigs 1kb cov5
SRR11091006 contigs 1kb cov5
GCA 015681035v1
GCA 005566315v1
GCA 008478325v1
GCA 010532815v1
GCA 011175875v1
GCA 019524745v1
SRR8984741 contigs 1kb cov5
SRR8618798 contigs 1kb cov5
GCA 018833325v1
SRR8866230 contigs 1kb cov5
GCA 006823465v1
GCA 018959375v1
GCA 011592125v1
GCA 010262465v1
GCA 014569575v1
GCA 005636275v1
GCA 005784235v1
GCA 008299565v1
GCA 006576945v1
GCA 007424625v1
SRR6791825 contigs 1kb cov5
GCA 014565035v1
GCA 008528235v1
GCA 005589735v1
GCA 006047095v1
GCA 006034455v1
GCA 006045535v1
GCA 007103065v1
GCA 006259555v1
GCA 006616805v1
GCA 006928195v1
GCA 007643875v1
GCA 008405985v1
GCA 009411865v1
GCA 010970035v1
GCA 011128355v1
GCA 011137915v1

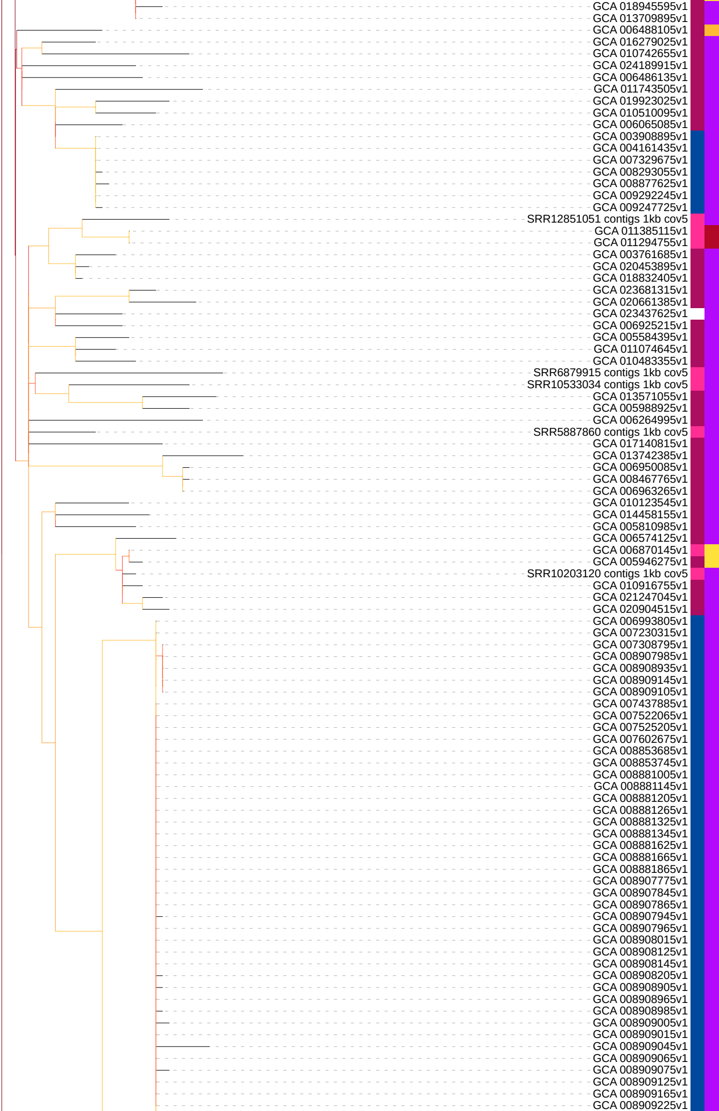
GCA 018945595v1
GCA 013709895v1
GCA 006488105v1
GCA 016279025v1
GCA 010742655v1
GCA 024189915v1
GCA 006486135v1
GCA 011743505v1
GCA 019923025v1
GCA 010510095v1
GCA 006065085v1
GCA 003908895v1
GCA 004161435v1
GCA 007329675v1
GCA 008293055v1
GCA 008877625v1
GCA 009292245v1
GCA 009247725v1
SRR12851051 contigs 1kb cov5
GCA 011385115v1
GCA 011294755v1
GCA 003761685v1
GCA 020453895v1
GCA 018832405v1
GCA 023681315v1
GCA 020661385v1
GCA 023437625v1
GCA 006925215v1
GCA 005584395v1
GCA 011074645v1
GCA 010483355v1
SRR6879915 contigs 1kb cov5
SRR10533034 contigs 1kb cov5
GCA 013571055v1
GCA 005988925v1
GCA 006264995v1
SRR5887860 contigs 1kb cov5
GCA 017140815v1
GCA 013742385v1
GCA 006950085v1
GCA 008467765v1
GCA 006963265v1
GCA 010123545v1
GCA 014458155v1
GCA 005810985v1
GCA 006574125v1
GCA 006870145v1
GCA 005946275v1
SRR10203120 contigs 1kb cov5
GCA 010916755v1
GCA 021247045v1
GCA 020904515v1
GCA 006993805v1
GCA 007230315v1
GCA 007308795v1
GCA 008907985v1
GCA 008908935v1
GCA 008909145v1
GCA 008909105v1
GCA 007437885v1
GCA 007522065v1
GCA 007525205v1
GCA 007602675v1
GCA 008853685v1
GCA 008853745v1
GCA 008881005v1
GCA 008881145v1
GCA 008881205v1
GCA 008881265v1
GCA 008881325v1
GCA 008881345v1
GCA 008881625v1
GCA 008881665v1
GCA 008881865v1
GCA 008907775v1
GCA 008907845v1
GCA 008907865v1
GCA 008907945v1
GCA 008907965v1
GCA 008908015v1
GCA 008908125v1
GCA 008908145v1
GCA 008908205v1
GCA 008908905v1
GCA 008908965v1
GCA 008908985v1
GCA 008909005v1
GCA 008909015v1
GCA 008909045v1
GCA 008909065v1
GCA 008909075v1
GCA 008909125v1
GCA 008909165v1
GCA 008909225v1

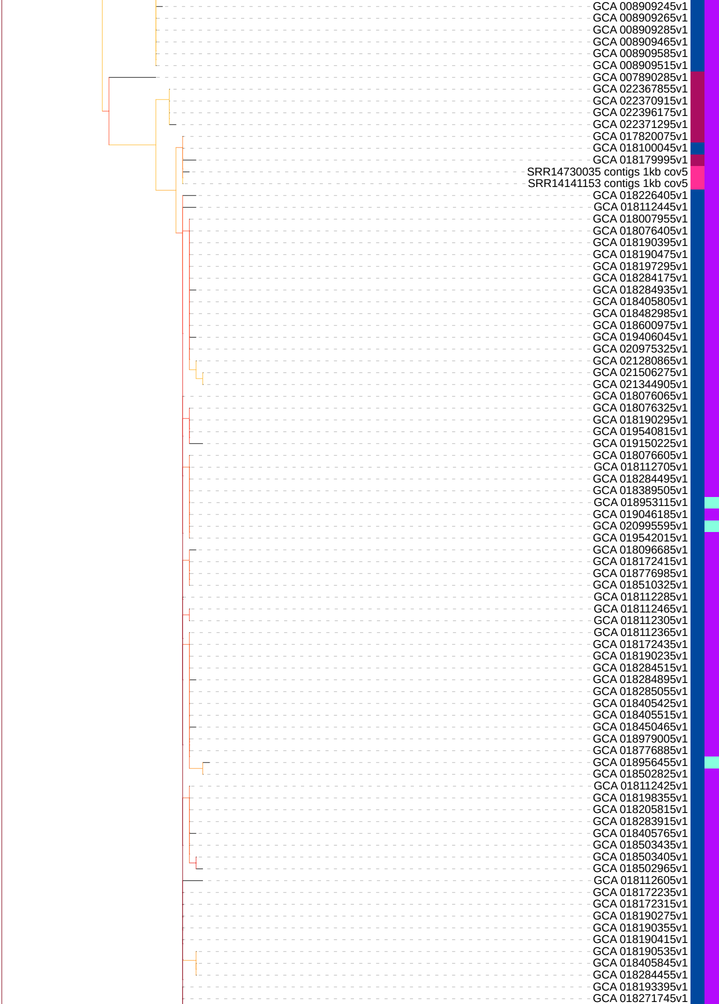
GCA 008909245v1
GCA 008909265v1
GCA 008909285v1
GCA 008909465v1
GCA 008909585v1
GCA 008909515v1
GCA 007890285v1
GCA 022367855v1
GCA 022370915v1
GCA 022396175v1
GCA 022371295v1
GCA 017820075v1
GCA 018100045v1
GCA 018179995v1
SRR14730035 contigs 1kb cov5
SRR14141153 contigs 1kb cov5
GCA 018226405v1
GCA 018112445v1
GCA 018007955v1
GCA 018076405v1
GCA 018190395v1
GCA 018190475v1
GCA 018197295v1
GCA 018284175v1
GCA 018284935v1
GCA 018405805v1
GCA 018482985v1
GCA 018600975v1
GCA 019406045v1
GCA 020975325v1
GCA 021280865v1
GCA 021506275v1
GCA 021344905v1
GCA 018076065v1
GCA 018076325v1
GCA 018190295v1
GCA 019540815v1
GCA 019150225v1
GCA 018076605v1
GCA 018112705v1
GCA 018284495v1
GCA 018389505v1
GCA 018953115v1
GCA 019046185v1
GCA 020995595v1
GCA 019542015v1
GCA 018096685v1
GCA 018172415v1
GCA 018776985v1
GCA 018510325v1
GCA 018112285v1
GCA 018112465v1
GCA 018112305v1
GCA 018112365v1
GCA 018172435v1
GCA 018190235v1
GCA 018284515v1
GCA 018284895v1
GCA 018285055v1
GCA 018405425v1
GCA 018405515v1
GCA 018450465v1
GCA 018979005v1
GCA 018776885v1
GCA 018956455v1
GCA 018502825v1
GCA 018112425v1
GCA 018198355v1
GCA 018205815v1
GCA 018283915v1
GCA 018405765v1
GCA 018503435v1
GCA 018503405v1
GCA 018502965v1
GCA 018112605v1
GCA 018172235v1
GCA 018172315v1
GCA 018190275v1
GCA 018190355v1
GCA 018190415v1
GCA 018190535v1
GCA 018405845v1
GCA 018284455v1
GCA 018193395v1
GCA 018271745v1

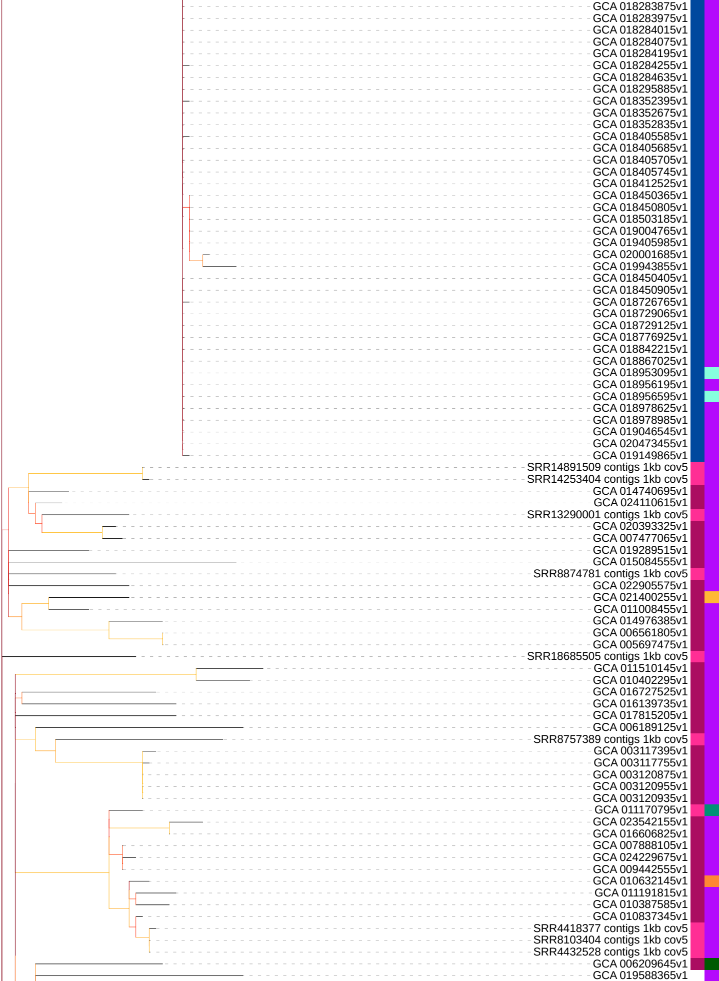
GCA 018283875v1
GCA 018283975v1
GCA 018284015v1
GCA 018284075v1
GCA 018284195v1
GCA 018284255v1
GCA 018284635v1
GCA 018295885v1
GCA 018352395v1
GCA 018352675v1
GCA 018352835v1
GCA 018405585v1
GCA 018405685v1
GCA 018405705v1
GCA 018405745v1
GCA 018412525v1
GCA 018450365v1
GCA 018450805v1
GCA 018503185v1
GCA 019004765v1
GCA 019405985v1
GCA 020001685v1
GCA 019943855v1
GCA 018450405v1
GCA 018450905v1
GCA 018726765v1
GCA 018729065v1
GCA 018729125v1
GCA 018776925v1
GCA 018842215v1
GCA 018867025v1
GCA 018953095v1
GCA 018956195v1
GCA 018956595v1
GCA 018978625v1
GCA 018978985v1
GCA 019046545v1
GCA 020473455v1
GCA 019149865v1
SRR14891509 contigs 1kb cov5
SRR14253404 contigs 1kb cov5
GCA 014740695v1
GCA 024110615v1
SRR13290001 contigs 1kb cov5
GCA 020393325v1
GCA 007477065v1
GCA 019289515v1
GCA 015084555v1
SRR8874781 contigs 1kb cov5
GCA 022905575v1
GCA 021400255v1
GCA 011008455v1
GCA 014976385v1
GCA 006561805v1
GCA 005697475v1
SRR18685505 contigs 1kb cov5
GCA 011510145v1
GCA 010402295v1
GCA 016727525v1
GCA 016139735v1
GCA 017815205v1
GCA 006189125v1
SRR8757389 contigs 1kb cov5
GCA 003117395v1
GCA 003117755v1
GCA 003120875v1
GCA 003120955v1
GCA 003120935v1
GCA 011170795v1
GCA 023542155v1
GCA 016606825v1
GCA 007888105v1
GCA 024229675v1
GCA 009442555v1
GCA 010632145v1
GCA 011191815v1
GCA 010387585v1
GCA 010837345v1
SRR4418377 contigs 1kb cov5
SRR8103404 contigs 1kb cov5
SRR4432528 contigs 1kb cov5
GCA 006209645v1
GCA 019588365v1

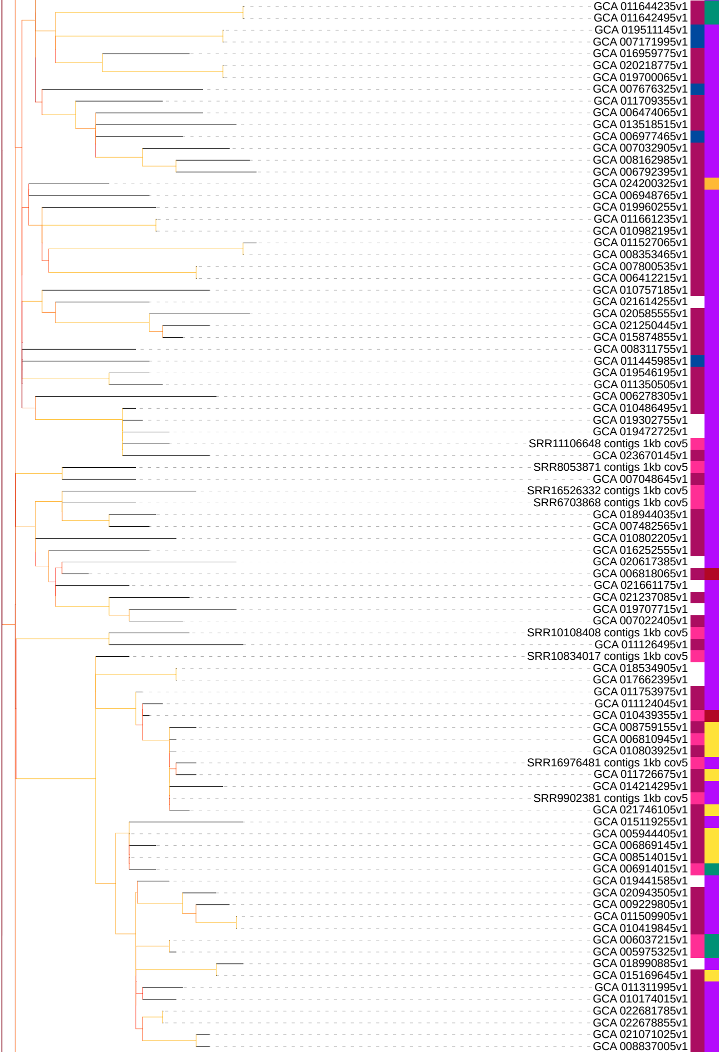
GCA 011644235v1
GCA 011642495v1
GCA 019511145v1
GCA 007171995v1
GCA 016959775v1
GCA 020218775v1
GCA 019700065v1
GCA 007676325v1
GCA 011709355v1
GCA 006474065v1
GCA 013518515v1
GCA 006977465v1
GCA 007032905v1
GCA 008162985v1
GCA 006792395v1
GCA 024200325v1
GCA 006948765v1
GCA 019960255v1
GCA 011661235v1
GCA 010982195v1
GCA 011527065v1
GCA 008353465v1
GCA 007800535v1
GCA 006412215v1
GCA 010757185v1
GCA 021614255v1
GCA 020585555v1
GCA 021250445v1
GCA 015874855v1
GCA 008311755v1
GCA 011445985v1
GCA 019546195v1
GCA 011350505v1
GCA 006278305v1
GCA 010486495v1
GCA 019302755v1
GCA 019472725v1
SRR11106648 contigs 1kb cov5
GCA 023670145v1
SRR8053871 contigs 1kb cov5
GCA 007048645v1
SRR16526332 contigs 1kb cov5
SRR6703868 contigs 1kb cov5
GCA 018944035v1
GCA 007482565v1
GCA 010802205v1
GCA 016252555v1
GCA 020617385v1
GCA 006818065v1
GCA 021661175v1
GCA 021237085v1
GCA 019707715v1
GCA 007022405v1
SRR10108408 contigs 1kb cov5
GCA 011126495v1
SRR10834017 contigs 1kb cov5
GCA 018534905v1
GCA 017662395v1
GCA 011753975v1
GCA 011124045v1
GCA 010439355v1
GCA 008759155v1
GCA 006810945v1
GCA 010803925v1
SRR16976481 contigs 1kb cov5
GCA 011726675v1
GCA 014214295v1
SRR9902381 contigs 1kb cov5
GCA 021746105v1
GCA 015119255v1
GCA 005944405v1
GCA 006869145v1
GCA 008514015v1
GCA 006914015v1
GCA 019441585v1
GCA 020943505v1
GCA 009229805v1
GCA 011509905v1
GCA 010419845v1
GCA 006037215v1
GCA 005975325v1
GCA 018990885v1
GCA 015169645v1
GCA 011311995v1
GCA 010174015v1
GCA 022681785v1
GCA 022678855v1
GCA 021071025v1
GCA 008837005v1

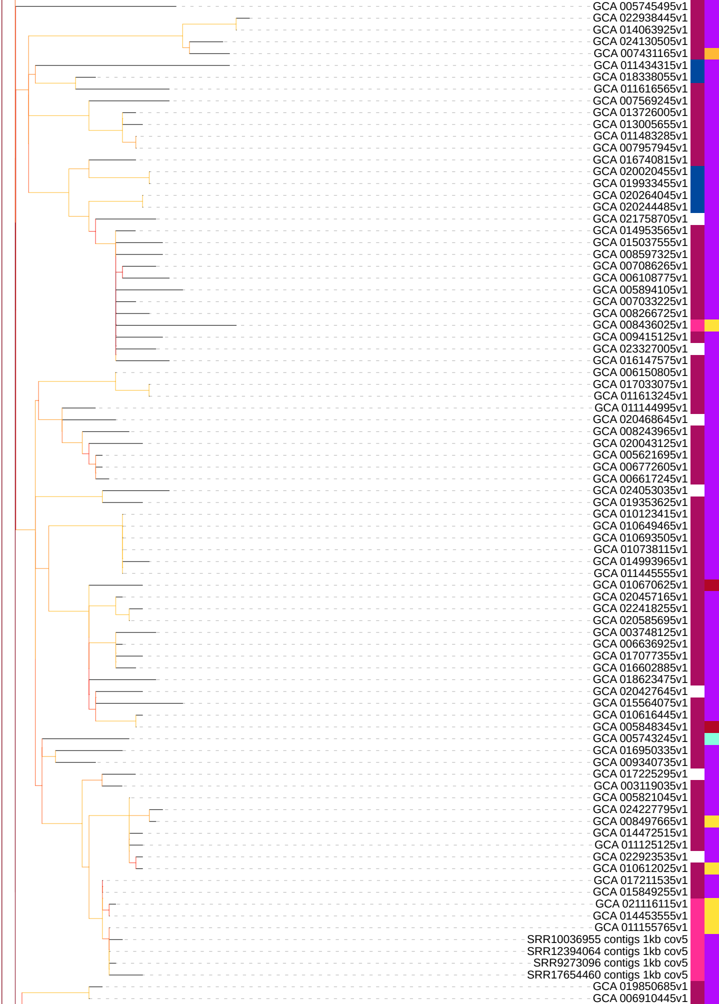
GCA 005745495v1
GCA 022938445v1
GCA 014063925v1
GCA 024130505v1
GCA 007431165v1
GCA 011434315v1
GCA 018338055v1
GCA 011616565v1
GCA 007569245v1
GCA 013726005v1
GCA 013005655v1
GCA 011483285v1
GCA 007957945v1
GCA 016740815v1
GCA 020020455v1
GCA 019933455v1
GCA 020264045v1
GCA 020244485v1
GCA 021758705v1
GCA 014953565v1
GCA 015037555v1
GCA 008597325v1
GCA 007086265v1
GCA 006108775v1
GCA 005894105v1
GCA 007033225v1
GCA 008266725v1
GCA 008436025v1
GCA 009415125v1
GCA 023327005v1
GCA 016147575v1
GCA 006150805v1
GCA 017033075v1
GCA 011613245v1
GCA 011144995v1
GCA 020468645v1
GCA 008243965v1
GCA 020043125v1
GCA 005621695v1
GCA 006772605v1
GCA 006617245v1
GCA 024053035v1
GCA 019353625v1
GCA 010123415v1
GCA 010649465v1
GCA 010693505v1
GCA 010738115v1
GCA 014993965v1
GCA 011445555v1
GCA 010670625v1
GCA 020457165v1
GCA 022418255v1
GCA 020585695v1
GCA 003748125v1
GCA 006636925v1
GCA 017077355v1
GCA 016602885v1
GCA 018623475v1
GCA 020427645v1
GCA 015564075v1
GCA 010616445v1
GCA 005848345v1
GCA 005743245v1
GCA 016950335v1
GCA 009340735v1
GCA 017225295v1
GCA 003119035v1
GCA 005821045v1
GCA 024227795v1
GCA 008497665v1
GCA 014472515v1
GCA 011125125v1
GCA 022923535v1
GCA 010612025v1
GCA 017211535v1
GCA 015849255v1
GCA 021116115v1
GCA 014453555v1
GCA 011155765v1
SRR10036955 contigs 1kb cov5
SRR12394064 contigs 1kb cov5
SRR9273096 contigs 1kb cov5
SRR17654460 contigs 1kb cov5
GCA 019850685v1
GCA 006910445v1

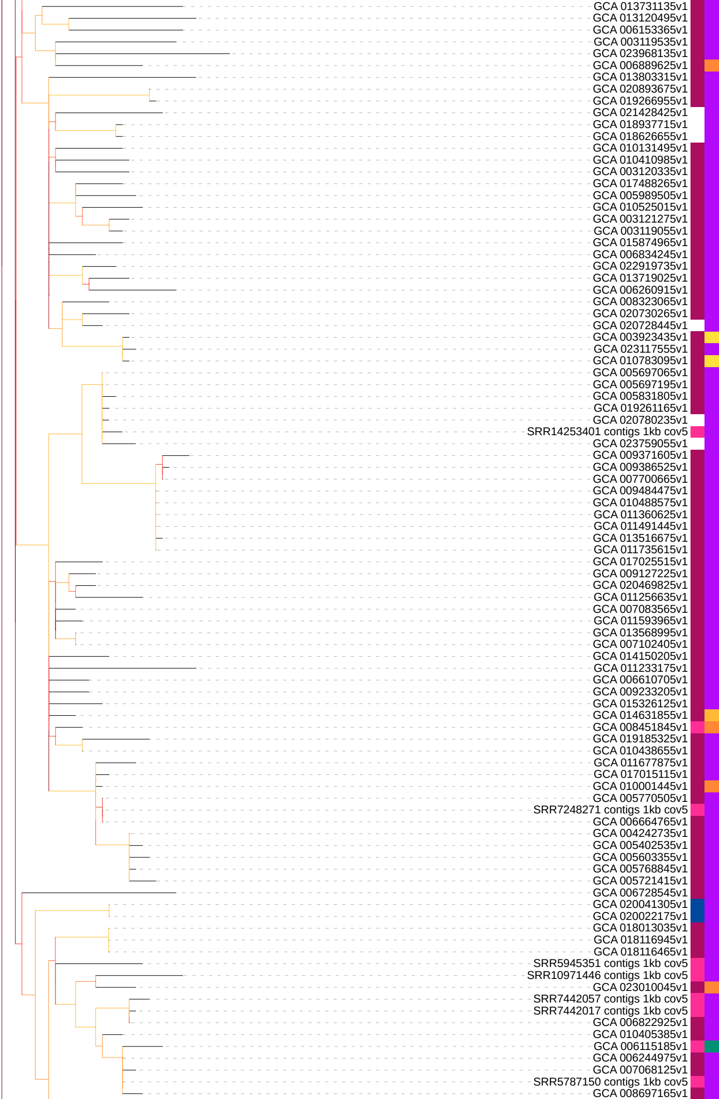
GCA 013731135v1
GCA 013120495v1
GCA 006153365v1
GCA 003119535v1
GCA 023968135v1
GCA 006889625v1
GCA 013803315v1
GCA 020893675v1
GCA 019266955v1
GCA 021428425v1
GCA 018937715v1
GCA 018626655v1
GCA 010131495v1
GCA 010410985v1
GCA 003120335v1
GCA 017488265v1
GCA 005989505v1
GCA 010525015v1
GCA 003121275v1
GCA 003119055v1
GCA 015874965v1
GCA 006834245v1
GCA 022919735v1
GCA 013719025v1
GCA 006260915v1
GCA 008323065v1
GCA 020730265v1
GCA 020728445v1
GCA 003923435v1
GCA 023117555v1
GCA 010783095v1
GCA 005697065v1
GCA 005697195v1
GCA 005831805v1
GCA 019261165v1
GCA 020780235v1
SRR14253401 contigs 1kb cov5
GCA 023759055v1
GCA 009371605v1
GCA 009386525v1
GCA 007700665v1
GCA 009484475v1
GCA 010488575v1
GCA 011360625v1
GCA 011491445v1
GCA 013516675v1
GCA 011735615v1
GCA 017025515v1
GCA 009127225v1
GCA 020469825v1
GCA 011256635v1
GCA 007083565v1
GCA 011593965v1
GCA 013568995v1
GCA 007102405v1
GCA 014150205v1
GCA 011233175v1
GCA 006610705v1
GCA 009233205v1
GCA 015326125v1
GCA 014631855v1
GCA 008451845v1
GCA 019185325v1
GCA 010438655v1
GCA 011677875v1
GCA 017015115v1
GCA 010001445v1
GCA 005770505v1
SRR7248271 contigs 1kb cov5
GCA 006664765v1
GCA 004242735v1
GCA 005402535v1
GCA 005603355v1
GCA 005768845v1
GCA 005721415v1
GCA 006728545v1
GCA 020041305v1
GCA 020022175v1
GCA 018013035v1
GCA 018116945v1
GCA 018116465v1
SRR5945351 contigs 1kb cov5
SRR10971446 contigs 1kb cov5
GCA 023010045v1
SRR7442057 contigs 1kb cov5
SRR7442017 contigs 1kb cov5
GCA 006822925v1
GCA 010405385v1
GCA 006115185v1
GCA 006244975v1
GCA 007068125v1
SRR5787150 contigs 1kb cov5
GCA 008697165v1

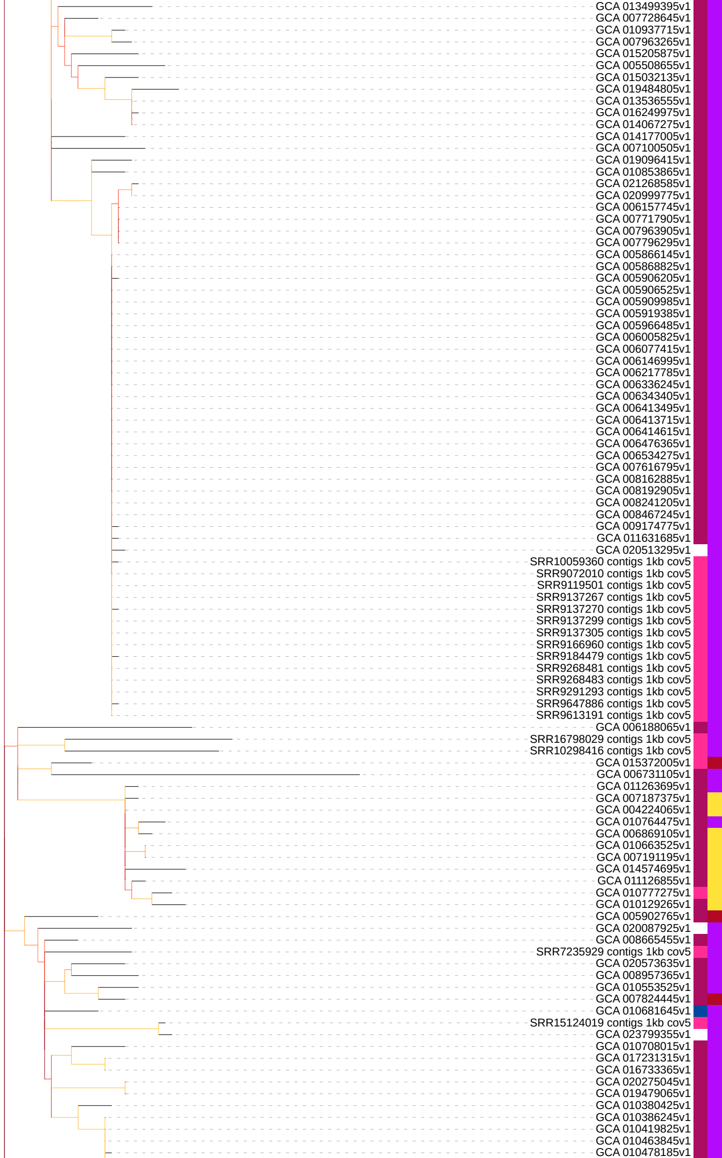
GCA 013499395v1
GCA 007728645v1
GCA 010937715v1
GCA 007963265v1
GCA 015205875v1
GCA 005508655v1
GCA 015032135v1
GCA 019484805v1
GCA 013536555v1
GCA 016249975v1
GCA 014067275v1
GCA 014177005v1
GCA 007100505v1
GCA 019096415v1
GCA 010853865v1
GCA 021268585v1
GCA 020999775v1
GCA 006157745v1
GCA 007717905v1
GCA 007963905v1
GCA 007796295v1
GCA 005866145v1
GCA 005868825v1
GCA 005906205v1
GCA 005906525v1
GCA 005909985v1
GCA 005919385v1
GCA 005966485v1
GCA 006005825v1
GCA 006077415v1
GCA 006146995v1
GCA 006217785v1
GCA 006336245v1
GCA 006343405v1
GCA 006413495v1
GCA 006413715v1
GCA 006414615v1
GCA 006476365v1
GCA 006534275v1
GCA 007616795v1
GCA 008162885v1
GCA 008192905v1
GCA 008241205v1
GCA 008467245v1
GCA 009174775v1
GCA 011631685v1
GCA 020513295v1
SRR10059360 contigs 1kb cov5
SRR9072010 contigs 1kb cov5
SRR9119501 contigs 1kb cov5
SRR9137267 contigs 1kb cov5
SRR9137270 contigs 1kb cov5
SRR9137299 contigs 1kb cov5
SRR9137305 contigs 1kb cov5
SRR9166960 contigs 1kb cov5
SRR9184479 contigs 1kb cov5
SRR9268481 contigs 1kb cov5
SRR9268483 contigs 1kb cov5
SRR9291293 contigs 1kb cov5
SRR9647886 contigs 1kb cov5
SRR9613191 contigs 1kb cov5
GCA 006188065v1
SRR16798029 contigs 1kb cov5
SRR10298416 contigs 1kb cov5
GCA 015372005v1
GCA 006731105v1
GCA 011263695v1
GCA 007187375v1
GCA 004224065v1
GCA 010764475v1
GCA 006869105v1
GCA 010663525v1
GCA 007191195v1
GCA 014574695v1
GCA 011126855v1
GCA 010777275v1
GCA 010129265v1
GCA 005902765v1
GCA 020087925v1
GCA 008665455v1
SRR7235929 contigs 1kb cov5
GCA 020573635v1
GCA 008957365v1
GCA 010553525v1
GCA 007824445v1
GCA 010681645v1
SRR15124019 contigs 1kb cov5
GCA 023799355v1
GCA 010708015v1
GCA 017231315v1
GCA 016733365v1
GCA 020275045v1
GCA 019479065v1
GCA 010380425v1
GCA 010386245v1
GCA 010419825v1
GCA 010463845v1
GCA 010478185v1

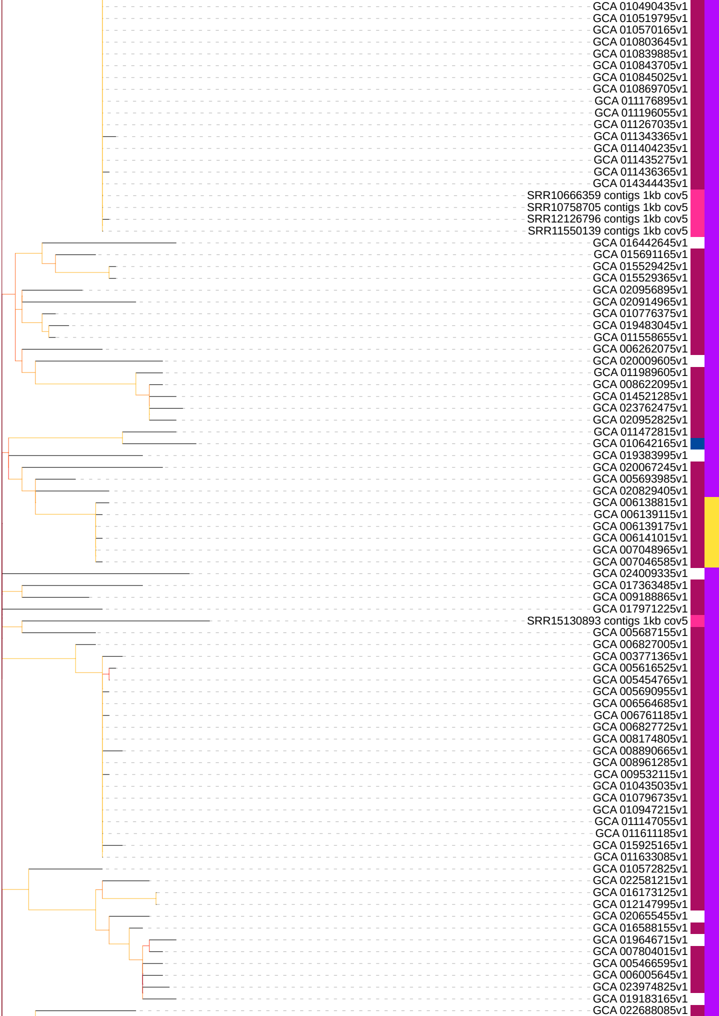
GCA 010490435v1
GCA 010519795v1
GCA 010570165v1
GCA 010803645v1
GCA 010839885v1
GCA 010843705v1
GCA 010845025v1
GCA 010869705v1
GCA 011176895v1
GCA 011196055v1
GCA 011267035v1
GCA 011343365v1
GCA 011404235v1
GCA 011435275v1
GCA 011436365v1
GCA 014344435v1
SRR10666359 contigs 1kb cov5
SRR10758705 contigs 1kb cov5
SRR12126796 contigs 1kb cov5
SRR11550139 contigs 1kb cov5
GCA 016442645v1
GCA 015691165v1
GCA 015529425v1
GCA 015529365v1
GCA 020956895v1
GCA 020914965v1
GCA 010776375v1
GCA 019483045v1
GCA 011558655v1
GCA 006262075v1
GCA 020009605v1
GCA 011989605v1
GCA 008622095v1
GCA 014521285v1
GCA 023762475v1
GCA 020952825v1
GCA 011472815v1
GCA 010642165v1
GCA 019383995v1
GCA 020067245v1
GCA 005693985v1
GCA 020829405v1
GCA 006138815v1
GCA 006139115v1
GCA 006139175v1
GCA 006141015v1
GCA 007048965v1
GCA 007046585v1
GCA 024009335v1
GCA 017363485v1
GCA 009188865v1
GCA 017971225v1
SRR15130893 contigs 1kb cov5
GCA 005687155v1
GCA 006827005v1
GCA 003771365v1
GCA 005616525v1
GCA 005454765v1
GCA 005690955v1
GCA 006564685v1
GCA 006761185v1
GCA 006827725v1
GCA 008174805v1
GCA 008890665v1
GCA 008961285v1
GCA 009532115v1
GCA 010435035v1
GCA 010796735v1
GCA 010947215v1
GCA 011147055v1
GCA 011611185v1
GCA 015925165v1
GCA 011633085v1
GCA 010572825v1
GCA 022581215v1
GCA 016173125v1
GCA 012147995v1
GCA 020655455v1
GCA 016588155v1
GCA 019646715v1
GCA 007804015v1
GCA 005466595v1
GCA 006005645v1
GCA 023974825v1
GCA 019183165v1
GCA 022688085v1

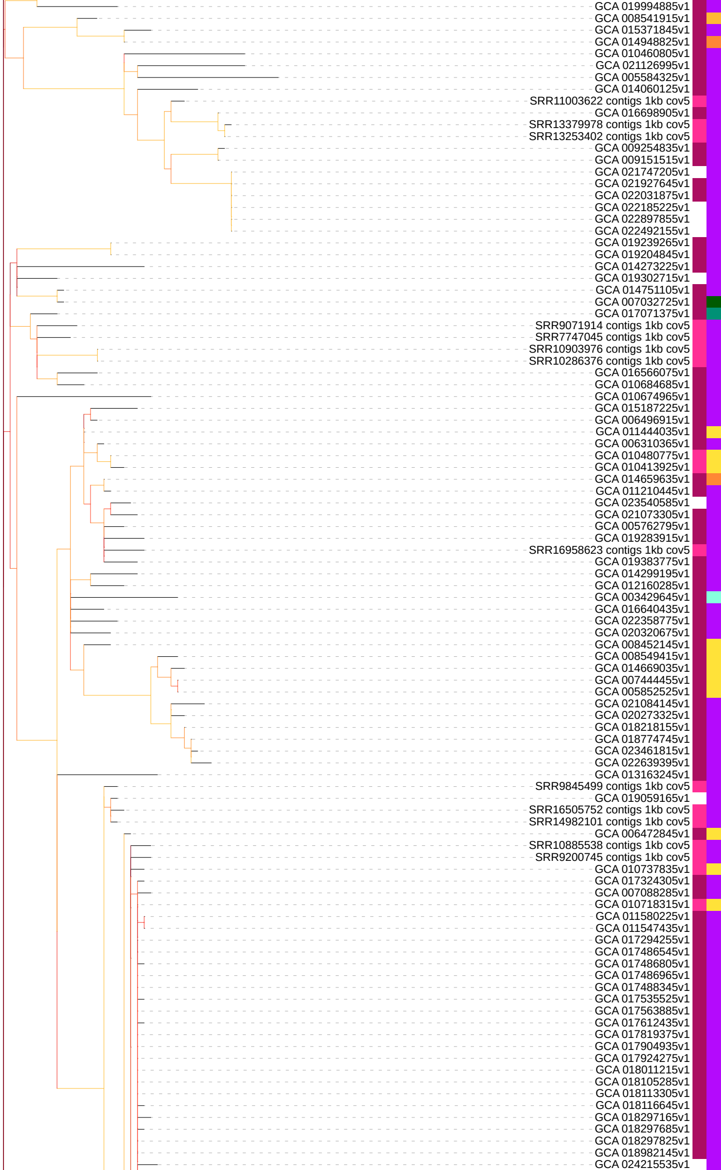
GCA 019994885v1
GCA 008541915v1
GCA 015371845v1
GCA 014948825v1
GCA 010460805v1
GCA 021126995v1
GCA 005584325v1
GCA 014060125v1
SRR11003622 contigs 1kb cov5
GCA 016698905v1
SRR13379978 contigs 1kb cov5
SRR13253402 contigs 1kb cov5
GCA 009254835v1
GCA 009151515v1
GCA 021747205v1
GCA 021927645v1
GCA 022031875v1
GCA 022185225v1
GCA 022897855v1
GCA 022492155v1
GCA 019239265v1
GCA 019204845v1
GCA 014273225v1
GCA 019302715v1
GCA 014751105v1
GCA 007032725v1
GCA 017071375v1
SRR9071914 contigs 1kb cov5
SRR7747045 contigs 1kb cov5
SRR10903976 contigs 1kb cov5
SRR10286376 contigs 1kb cov5
GCA 016566075v1
GCA 010684685v1
GCA 010674965v1
GCA 015187225v1
GCA 006496915v1
GCA 011444035v1
GCA 006310365v1
GCA 010480775v1
GCA 010413925v1
GCA 014659635v1
GCA 011210445v1
GCA 023540585v1
GCA 021073305v1
GCA 005762795v1
GCA 019283915v1
SRR16958623 contigs 1kb cov5
GCA 019383775v1
GCA 014299195v1
GCA 012160285v1
GCA 003429645v1
GCA 016640435v1
GCA 022358775v1
GCA 020320675v1
GCA 008452145v1
GCA 008549415v1
GCA 014669035v1
GCA 007444455v1
GCA 005852525v1
GCA 021084145v1
GCA 020273325v1
GCA 018218155v1
GCA 018774745v1
GCA 023461815v1
GCA 022639395v1
GCA 013163245v1
SRR9845499 contigs 1kb cov5
GCA 019059165v1
SRR16505752 contigs 1kb cov5
SRR14982101 contigs 1kb cov5
GCA 006472845v1
SRR10885538 contigs 1kb cov5
SRR9200745 contigs 1kb cov5
GCA 010737835v1
GCA 017324305v1
GCA 007088285v1
GCA 010718315v1
GCA 011580225v1
GCA 011547435v1
GCA 017294255v1
GCA 017486545v1
GCA 017486805v1
GCA 017486965v1
GCA 017488345v1
GCA 017535525v1
GCA 017563885v1
GCA 017612435v1
GCA 017819375v1
GCA 017904935v1
GCA 017924275v1
GCA 018011215v1
GCA 018105285v1
GCA 018113305v1
GCA 018116645v1
GCA 018297165v1
GCA 018297685v1
GCA 018297825v1
GCA 018982145v1
GCA 024215535v1

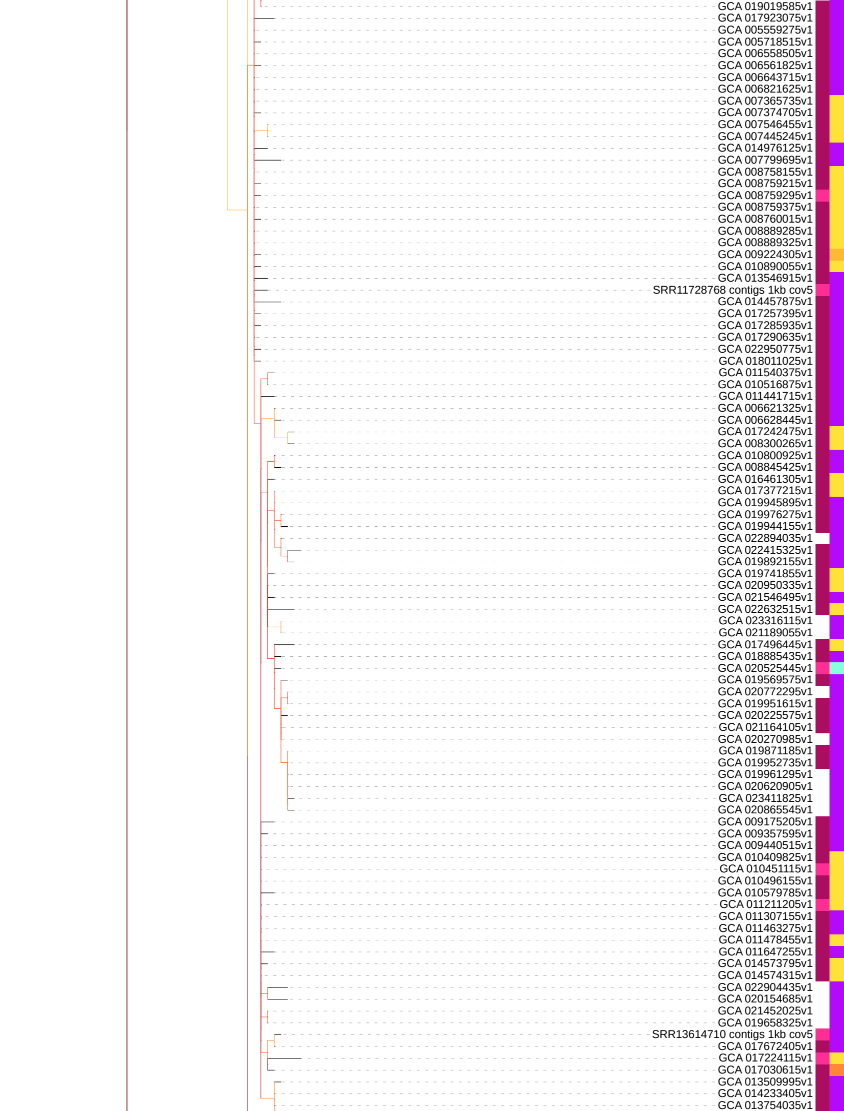
GCA 019019585v1
GCA 017923075v1
GCA 005559275v1
GCA 005718515v1
GCA 006558505v1
GCA 006561825v1
GCA 006643715v1
GCA 006821625v1
GCA 007365735v1
GCA 007374705v1
GCA 007546455v1
GCA 007445245v1
GCA 014976125v1
GCA 007799695v1
GCA 008758155v1
GCA 008759215v1
GCA 008759295v1
GCA 008759375v1
GCA 008760015v1
GCA 008889285v1
GCA 008889325v1
GCA 009224305v1
GCA 010890055v1
GCA 013546915v1
SRR11728768 contigs 1kb cov5
GCA 014457875v1
GCA 017257395v1
GCA 017285935v1
GCA 017290635v1
GCA 022950775v1
GCA 018011025v1
GCA 011540375v1
GCA 010516875v1
GCA 011441715v1
GCA 006621325v1
GCA 006628445v1
GCA 017242475v1
GCA 008300265v1
GCA 010800925v1
GCA 008845425v1
GCA 016461305v1
GCA 017377215v1
GCA 019945895v1
GCA 019976275v1
GCA 019944155v1
GCA 022894035v1
GCA 022415325v1
GCA 019892155v1
GCA 019741855v1
GCA 020950335v1
GCA 021546495v1
GCA 022632515v1
GCA 023316115v1
GCA 021189055v1
GCA 017496445v1
GCA 018885435v1
GCA 020525445v1
GCA 019569575v1
GCA 020772295v1
GCA 019951615v1
GCA 020225575v1
GCA 021164105v1
GCA 020270985v1
GCA 019871185v1
GCA 019952735v1
GCA 019961295v1
GCA 020620905v1
GCA 023411825v1
GCA 020865545v1
GCA 009175205v1
GCA 009357595v1
GCA 009440515v1
GCA 010409825v1
GCA 010451115v1
GCA 010496155v1
GCA 010579785v1
GCA 011211205v1
GCA 011307155v1
GCA 011463275v1
GCA 011478455v1
GCA 011647255v1
GCA 014573795v1
GCA 014574315v1
GCA 022904435v1
GCA 020154685v1
GCA 021452025v1
GCA 019658325v1
SRR13614710 contigs 1kb cov5
GCA 017672405v1
GCA 017224115v1
GCA 017030615v1
GCA 013509995v1
GCA 014233405v1
GCA 013754035v1

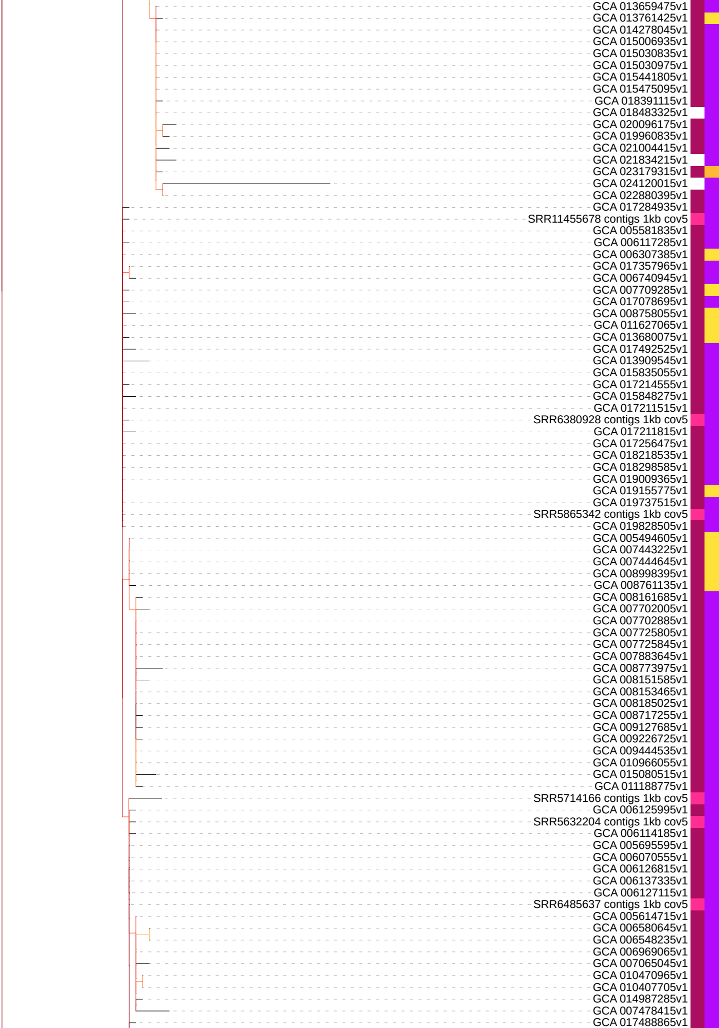
GCA 013659475v1
GCA 013761425v1
GCA 014278045v1
GCA 015006935v1
GCA 015030835v1
GCA 015030975v1
GCA 015441805v1
GCA 015475095v1
GCA 018391115v1
GCA 018483325v1
GCA 020096175v1
GCA 019960835v1
GCA 021004415v1
GCA 021834215v1
GCA 023179315v1
GCA 024120015v1
GCA 022880395v1
GCA 017284935v1
SRR11455678 contigs 1kb cov5
GCA 005581835v1
GCA 006117285v1
GCA 006307385v1
GCA 017357965v1
GCA 006740945v1
GCA 007709285v1
GCA 017078695v1
GCA 008758055v1
GCA 011627065v1
GCA 013680075v1
GCA 017492525v1
GCA 013909545v1
GCA 015835055v1
GCA 017214555v1
GCA 015848275v1
GCA 017211515v1
SRR6380928 contigs 1kb cov5
GCA 017211815v1
GCA 017256475v1
GCA 018218535v1
GCA 018298585v1
GCA 019009365v1
GCA 019155775v1
GCA 019737515v1
SRR5865342 contigs 1kb cov5
GCA 019828505v1
GCA 005494605v1
GCA 007443225v1
GCA 007444645v1
GCA 008998395v1
GCA 008761135v1
GCA 008161685v1
GCA 007702005v1
GCA 007702885v1
GCA 007725805v1
GCA 007725845v1
GCA 007883645v1
GCA 008773975v1
GCA 008151585v1
GCA 008153465v1
GCA 008185025v1
GCA 008717255v1
GCA 009127685v1
GCA 009226725v1
GCA 009444535v1
GCA 010966055v1
GCA 015080515v1
GCA 011188775v1
SRR5714166 contigs 1kb cov5
GCA 006125995v1
SRR5632204 contigs 1kb cov5
GCA 006114185v1
GCA 005695595v1
GCA 006070555v1
GCA 006126815v1
GCA 006137335v1
GCA 006127115v1
SRR6485637 contigs 1kb cov5
GCA 005614715v1
GCA 006580645v1
GCA 006548235v1
GCA 006969065v1
GCA 007065045v1
GCA 010470965v1
GCA 010407705v1
GCA 014987285v1
GCA 007478415v1
GCA 017488865v1

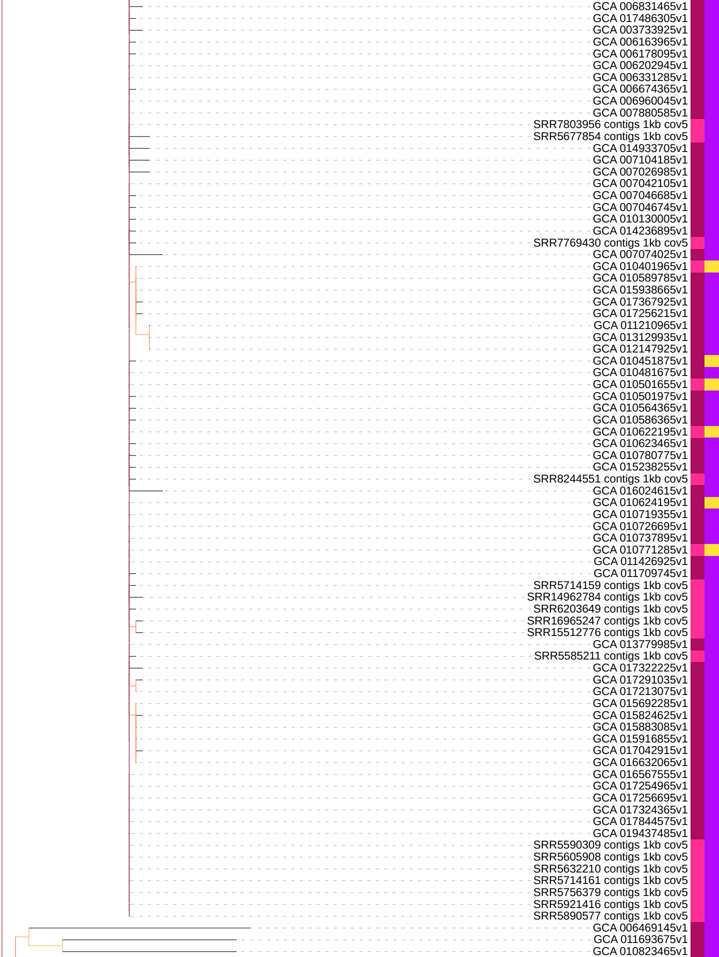
GCA 006831465v1
GCA 017486305v1
GCA 003733925v1
GCA 006163965v1
GCA 006178095v1
GCA 006202945v1
GCA 006331285v1
GCA 006674365v1
GCA 006960045v1
GCA 007880585v1
SRR7803956 contigs 1kb cov5
SRR5677854 contigs 1kb cov5
GCA 014933705v1
GCA 007104185v1
GCA 007026985v1
GCA 007042105v1
GCA 007046685v1
GCA 007046745v1
GCA 010130005v1
GCA 014236895v1
SRR7769430 contigs 1kb cov5
GCA 007074025v1
GCA 010401965v1
GCA 010589785v1
GCA 015938665v1
GCA 017367925v1
GCA 017256215v1
GCA 011210965v1
GCA 013129935v1
GCA 012147925v1
GCA 010451875v1
GCA 010481675v1
GCA 010501655v1
GCA 010501975v1
GCA 010564365v1
GCA 010586365v1
GCA 010622195v1
GCA 010623465v1
GCA 010780775v1
GCA 015238255v1
SRR8244551 contigs 1kb cov5
GCA 016024615v1
GCA 010624195v1
GCA 010719355v1
GCA 010726695v1
GCA 010737895v1
GCA 010771285v1
GCA 011426925v1
GCA 011709745v1
SRR5714159 contigs 1kb cov5
SRR14962784 contigs 1kb cov5
SRR6203649 contigs 1kb cov5
SRR16965247 contigs 1kb cov5
SRR15512776 contigs 1kb cov5
GCA 013779985v1
SRR5585211 contigs 1kb cov5
GCA 017322225v1
GCA 017291035v1
GCA 017213075v1
GCA 015692285v1
GCA 015824625v1
GCA 015883085v1
GCA 015916855v1
GCA 017042915v1
GCA 016632065v1
GCA 016567555v1
GCA 017254965v1
GCA 017256695v1
GCA 017324365v1
GCA 017844575v1
GCA 019437485v1
SRR5590309 contigs 1kb cov5
SRR5605908 contigs 1kb cov5
SRR5632210 contigs 1kb cov5
SRR5714161 contigs 1kb cov5
SRR5756379 contigs 1kb cov5
SRR5921416 contigs 1kb cov5
SRR5890577 contigs 1kb cov5
GCA 006469145v1
GCA 011693675v1
GCA 010823465v1

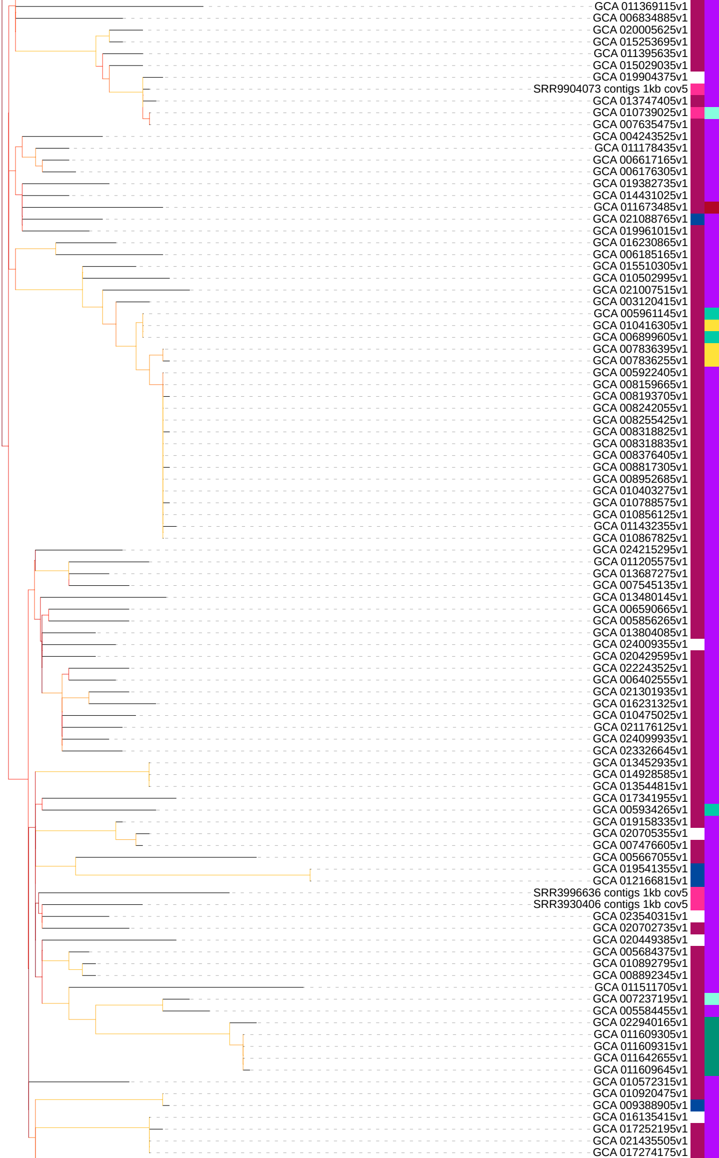
GCA 011369115v1
GCA 006834885v1
GCA 020005625v1
GCA 015253695v1
GCA 011395635v1
GCA 015029035v1
GCA 019904375v1
SRR9904073 contigs 1kb cov5
GCA 013747405v1
GCA 010739025v1
GCA 007635475v1
GCA 004243525v1
GCA 011178435v1
GCA 006617165v1
GCA 006176305v1
GCA 019382735v1
GCA 014431025v1
GCA 011673485v1
GCA 021088765v1
GCA 019961015v1
GCA 016230865v1
GCA 006185165v1
GCA 015510305v1
GCA 010502995v1
GCA 021007515v1
GCA 003120415v1
GCA 005961145v1
GCA 010416305v1
GCA 006899605v1
GCA 007836395v1
GCA 007836255v1
GCA 005922405v1
GCA 008159665v1
GCA 008193705v1
GCA 008242055v1
GCA 008255425v1
GCA 008318825v1
GCA 008318835v1
GCA 008376405v1
GCA 008817305v1
GCA 008952685v1
GCA 010403275v1
GCA 010788575v1
GCA 010856125v1
GCA 011432355v1
GCA 010867825v1
GCA 024215295v1
GCA 011205575v1
GCA 013687275v1
GCA 007545135v1
GCA 013480145v1
GCA 006590665v1
GCA 005856265v1
GCA 013804085v1
GCA 024009355v1
GCA 020429595v1
GCA 022243525v1
GCA 006402555v1
GCA 021301935v1
GCA 016231325v1
GCA 010475025v1
GCA 021176125v1
GCA 024099935v1
GCA 023326645v1
GCA 013452935v1
GCA 014928585v1
GCA 013544815v1
GCA 017341955v1
GCA 005934265v1
GCA 019158335v1
GCA 020705355v1
GCA 007476605v1
GCA 005667055v1
GCA 019541355v1
GCA 012166815v1
SRR3996636 contigs 1kb cov5
SRR3930406 contigs 1kb cov5
GCA 023540315v1
GCA 020702735v1
GCA 020449385v1
GCA 005684375v1
GCA 010892795v1
GCA 008892345v1
GCA 011511705v1
GCA 007237195v1
GCA 005584455v1
GCA 022940165v1
GCA 011609305v1
GCA 011609315v1
GCA 011642655v1
GCA 011609645v1
GCA 010572315v1
GCA 010920475v1
GCA 009388905v1
GCA 016135415v1
GCA 017252195v1
GCA 021435505v1
GCA 017274175v1

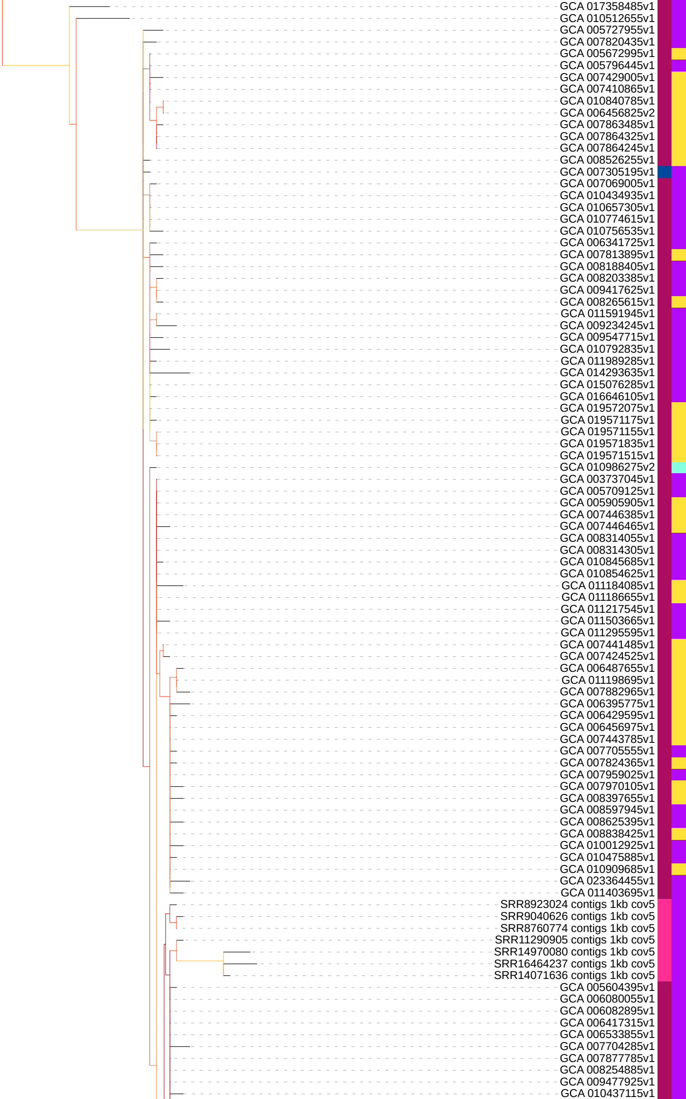
GCA 017358485v1
GCA 010512655v1
GCA 005727955v1
GCA 007820435v1
GCA 005672995v1
GCA 005796445v1
GCA 007429005v1
GCA 007410865v1
GCA 010840785v1
GCA 006456825v2
GCA 007863485v1
GCA 007864325v1
GCA 007864245v1
GCA 008526255v1
GCA 007305195v1
GCA 007069005v1
GCA 010434935v1
GCA 010657305v1
GCA 010774615v1
GCA 010756535v1
GCA 006341725v1
GCA 007813895v1
GCA 008188405v1
GCA 008203385v1
GCA 009417625v1
GCA 008265615v1
GCA 011591945v1
GCA 009234245v1
GCA 009547715v1
GCA 010792835v1
GCA 011989285v1
GCA 014293635v1
GCA 015076285v1
GCA 016646105v1
GCA 019572075v1
GCA 019571175v1
GCA 019571155v1
GCA 019571835v1
GCA 019571515v1
GCA 010986275v2
GCA 003737045v1
GCA 005709125v1
GCA 005905905v1
GCA 007446385v1
GCA 007446465v1
GCA 008314055v1
GCA 008314305v1
GCA 010845685v1
GCA 010854625v1
GCA 011184085v1
GCA 011186655v1
GCA 011217545v1
GCA 011503665v1
GCA 011295595v1
GCA 007441485v1
GCA 007424525v1
GCA 006487655v1
GCA 011198695v1
GCA 007882965v1
GCA 006395775v1
GCA 006429595v1
GCA 006456975v1
GCA 007443785v1
GCA 007705555v1
GCA 007824365v1
GCA 007959025v1
GCA 007970105v1
GCA 008397655v1
GCA 008597945v1
GCA 008625395v1
GCA 008838425v1
GCA 010012925v1
GCA 010475885v1
GCA 010909685v1
GCA 023364455v1
GCA 011403695v1
SRR8923024 contigs 1kb cov5
SRR9040626 contigs 1kb cov5
SRR8760774 contigs 1kb cov5
SRR11290905 contigs 1kb cov5
SRR14970080 contigs 1kb cov5
SRR16464237 contigs 1kb cov5
SRR14071636 contigs 1kb cov5
GCA 005604395v1
GCA 006080055v1
GCA 006082895v1
GCA 006417315v1
GCA 006533855v1
GCA 007704285v1
GCA 007877785v1
GCA 008254885v1
GCA 009477925v1
GCA 010437115v1

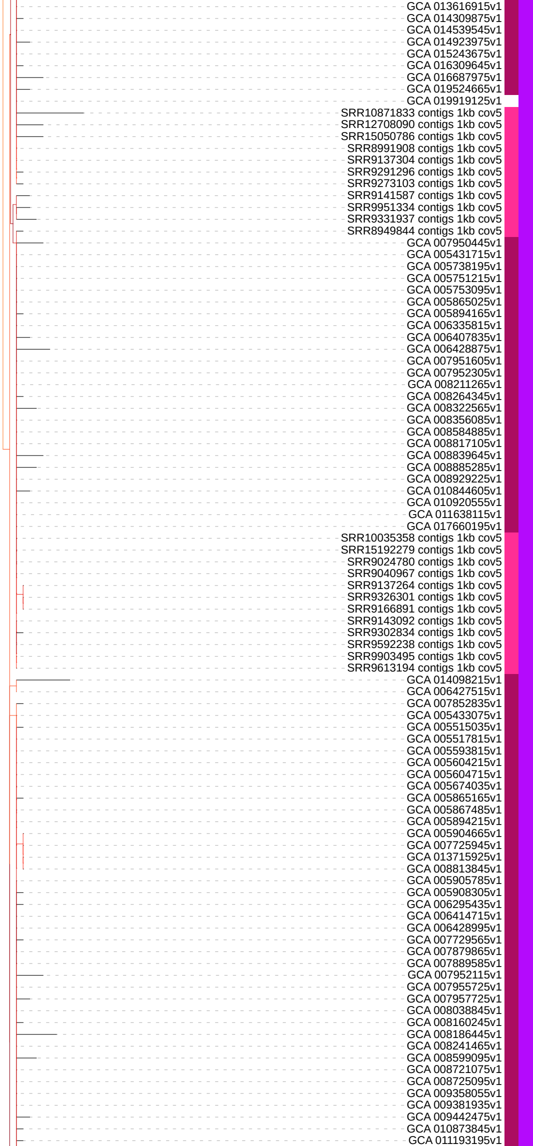
GCA 013616915v1
GCA 014309875v1
GCA 014539545v1
GCA 014923975v1
GCA 015243675v1
GCA 016309645v1
GCA 016687975v1
GCA 019524665v1
GCA 019919125v1
SRR10871833 contigs 1kb cov5
SRR12708090 contigs 1kb cov5
SRR15050786 contigs 1kb cov5
SRR8991908 contigs 1kb cov5
SRR9137304 contigs 1kb cov5
SRR9291296 contigs 1kb cov5
SRR9273103 contigs 1kb cov5
SRR9141587 contigs 1kb cov5
SRR9951334 contigs 1kb cov5
SRR9331937 contigs 1kb cov5
SRR8949844 contigs 1kb cov5
GCA 007950445v1
GCA 005431715v1
GCA 005738195v1
GCA 005751215v1
GCA 005753095v1
GCA 005865025v1
GCA 005894165v1
GCA 006335815v1
GCA 006407835v1
GCA 006428875v1
GCA 007951605v1
GCA 007952305v1
GCA 008211265v1
GCA 008264345v1
GCA 008322565v1
GCA 008356085v1
GCA 008584885v1
GCA 008817105v1
GCA 008839645v1
GCA 008885285v1
GCA 008929225v1
GCA 010844605v1
GCA 010920555v1
GCA 011638115v1
GCA 017660195v1
SRR10035358 contigs 1kb cov5
SRR15192279 contigs 1kb cov5
SRR9024780 contigs 1kb cov5
SRR9040967 contigs 1kb cov5
SRR9137264 contigs 1kb cov5
SRR9326301 contigs 1kb cov5
SRR9166891 contigs 1kb cov5
SRR9143092 contigs 1kb cov5
SRR9302834 contigs 1kb cov5
SRR9592238 contigs 1kb cov5
SRR9903495 contigs 1kb cov5
SRR9613194 contigs 1kb cov5
GCA 014098215v1
GCA 006427515v1
GCA 007852835v1
GCA 005433075v1
GCA 005515035v1
GCA 005517815v1
GCA 005593815v1
GCA 005604215v1
GCA 005604715v1
GCA 005674035v1
GCA 005865165v1
GCA 005867485v1
GCA 005894215v1
GCA 005904665v1
GCA 007725945v1
GCA 013715925v1
GCA 008813845v1
GCA 005905785v1
GCA 005908305v1
GCA 006295435v1
GCA 006414715v1
GCA 006428995v1
GCA 007729565v1
GCA 007879865v1
GCA 007889585v1
GCA 007952115v1
GCA 007955725v1
GCA 007957725v1
GCA 008038845v1
GCA 008160245v1
GCA 008186445v1
GCA 008241465v1
GCA 008599095v1
GCA 008721075v1
GCA 008725095v1
GCA 009358055v1
GCA 009381935v1
GCA 009442475v1
GCA 010873845v1
GCA 011193195v1

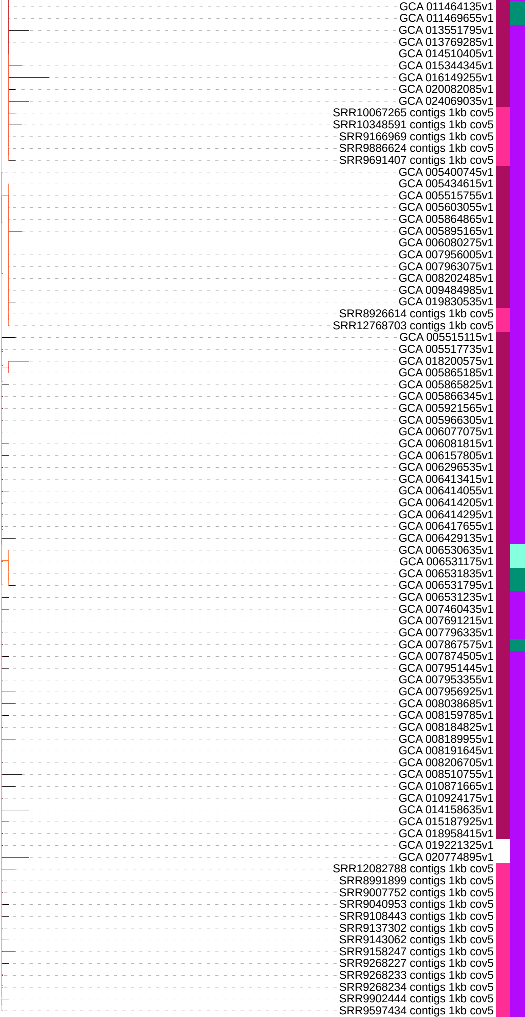

Fig. S2. Phylogenetic tree of Clade II isolates (n=2,261) with labeled isolate IDs, bootstrap values (0-1) and branch lengths.
